# Metabolites associated with abnormal glucose metabolism responding to primary care lifestyle intervention

**DOI:** 10.1038/s41598-025-25749-z

**Published:** 2025-11-07

**Authors:** Ville M. Koistinen, Suvi Manninen, Marjo Tuomainen, Kirsikka Aittola, Elina Järvelä-Reijonen, Tanja Tilles-Tirkkonen, Reija Männikkö, Niina Lintu, Leila Karhunen, Marjukka Kolehmainen, Santtu Mikkonen, Marko Lehtonen, Janne Martikainen, Kaisa Poutanen, Ursula Schwab, Pilvikki Absetz, Jaana Lindström, Timo A. Lakka, Kati Hanhineva, Jussi Pihlajamäki

**Affiliations:** 1 Institute of Public Health and Clinical Nutrition, University of Eastern Finland, Kuopio Campus, Kuopio, Finland; 2 Food Sciences Unit, University of Turku, Turku, Finland; 3 Institute of Biomedicine, University of Eastern Finland, Kuopio Campus, Kuopio, Finland; 4 Department of Environmental and Biological Sciences, University of Eastern Finland, Kuopio Campus, Kuopio, Finland; 5 Department of Technical Physics, University of Eastern Finland, Kuopio Campus, Kuopio, Finland; 6 School of Pharmacy, University of Eastern Finland, Kuopio Campus, Kuopio, Finland; 7 VTT Technical Research Centre of Finland Ltd, Espoo, Finland; 8 Department of Medicine, Endocrinology and Clinical Nutrition, Kuopio University Hospital, Wellbeing Services County of North Savo, Kuopio, Finland; 9 Health Sciences Unit, Faculty of Social Sciences, Tampere University, Tampere, Finland; 10 Department of Public Health and Welfare, Finnish Institute for Health and Welfare, Helsinki, Finland; 11 Department of Clinical Physiology and Nuclear Medicine, Kuopio University Hospital, Kuopio, Finland; 12 Foundation for Research in Health Exercise and Nutrition, Kuopio Research Institute of Exercise Medicine, Kuopio, Finland

**Keywords:** Metabolomics, Impaired glucose metabolism, Personalized treatment, Amino acids, Acylcarnitines, Phospholipids, Fatty acid amides, Metabolomics, Predictive markers, Type 2 diabetes

## Abstract

Type 2 diabetes can be prevented by lifestyle intervention. We aimed to identify metabolites that associate with glucose metabolism and respond to lifestyle intervention with evidence-based targets for nutrition and physical activity in individuals at high risk of type 2 diabetes. Standard oral glucose tolerance test (OGTT) was used to categorize 624 participants into those having normal glucose tolerance (NGT), isolated impaired glucose tolerance (IGT), IGT with increased fasting glucose (IGT + IFG), and type 2 diabetes. Plasma LC-MS metabolomics was performed to reveal metabolic signatures. The baseline group differences were analysed with the Kruskal–Wallis test and the effect of intervention with a linear mixed-effects model. Significant differences in the metabolite signature were observed between the baseline groups, particularly in amino acids, acylcarnitines, and phospholipids. Fatty acid amides, phospholipids, amino acids, dimethylguanidinovaleric acid, and 5-aminovaleric acid betaine responded most to the lifestyle intervention. Lysophosphatidylcholines containing odd-chain fatty acids showed associations with improved glucose metabolism. Twenty-five metabolites differed between the baseline groups, responded to the intervention, and were associated with changes in glucose metabolism. The findings suggest a metabolite panel could be used in distinguishing individuals with varying degrees of glucose metabolism and in predicting response to lifestyle interventions.

**Supplementary Information:**

The online version contains supplementary material available at 10.1038/s41598-025-25749-z.

## Introduction

Type 2 diabetes is a complex disorder that is associated with multiple metabolic abnormalities and can be prevented by lifestyle changes^1^. Many circulating metabolites have been shown to predict the risk of type 2 diabetes and be affected by lifestyle interventions^2,3^. Lifestyle interventions, which include dietary modification, increased physical activity, and weight loss, are central in type 2 diabetes prevention by improving insulin sensitivity and glycemic control. Landmark studies, such as the Diabetes Prevention Program (DPP) in the United States and the Finnish Diabetes Prevention Study (DPS), have shown that lifestyle changes can reduce the risk of developing type 2 diabetes by nearly 60% in high-risk individuals^4,5^. Metabolomics offers an opportunity to improve the early identification of those at increased risk of type 2 diabetes and may help understand the pathways relevant to disease prevention in response to a healthy lifestyle.

The Human Metabolome Database currently lists 21 unique plasma metabolites associated with type 2 diabetes^6^. Because type 2 diabetes is a preventable disease, the past metabolomics efforts have mostly focused on studying the pathophysiological processes potentially leading to or associated with the disease, such as impaired fasting glucose (IFG) and impaired glucose tolerance (IGT), to allow an early detection before the onset of the disease itself^7^. Several metabolites, including different amino acids, lipids and sugar metabolites, have been recognized as markers of impaired glucose metabolism^8^. For example, increased circulating concentrations of branched-chain amino acids and aromatic amino acids have been consistently associated with an increased risk of type 2 diabetes, whereas increased circulating levels of glycine, indolepropionic acid and several phosphatidylcholines have been associated with a decreased risk of type 2 diabetes^3,8,9^. Some of these metabolites associated with increased type 2 diabetes risk have been shown to change in response to lifestyle interventions^10^. Research has also been conducted on the metabolic impact of intervention studies, where *N*-acetyl-d-galactosamine, glycine betaine, methionine sulfoxide, 7-methylguanine, propionylcarnitine, putrescine, and serine have been associated with disease regression^11,12^.

Randomized controlled efficacy trials have been the main approach at investigating the effects of lifestyle interventions on the risk of developing type 2 diabetes or its risk factors. However, the implementation of the models developed in these studies for the prevention of type 2 diabetes in real-world and primary health care settings has been challenging. To this aim, we performed the Stop Diabetes (StopDia) study and developed a model for the prevention of type 2 diabetes as part of the Finnish healthcare system^13^. We were able to demonstrate the beneficial effects of combined digital intervention and group counselling on diet quality, waist circumference and fasting plasma insulin in individuals at increased risk for type 2 diabetes^14^, although there were no improvements in the blood glucose and weight.

In this study, we aimed to identify a metabolite pattern both associated with markers of glucose metabolism and impacted by a lifestyle intervention to identify metabolic pathways metabolic pathways that could be potential targets in personalized treatment of type 2 diabetes. First, we explored whether fasting plasma non-targeted metabolomics analysis could identify metabolites that advance the detection of abnormal glucose metabolism in individuals known to be at increased risk for type 2 diabetes based on the Finnish Diabetes Risk Score (FINDRISC) questionnaire^15^ by dividing the cohort into four baseline groups – normal glucose tolerance (NGT), isolated impaired glucose tolerance (IGT), impaired glucose tolerance with increased fasting glucose (IGT + IFG), and type 2 diabetes – based on their glucose metabolism markers according to the American Diabetes Association (ADA) criteria for the diagnosis of diabetes^16^. Second, we aimed to determine plasma metabolites that respond to a lifestyle intervention in a real-world setting in order to assess potential individual responses that could affect the potential impact of such an intervention.

## Methods

### Study design and participants

The StopDia study is a one-year, parallel-group, unblinded, multiple-setting randomized controlled trial (RCT) (ClinicalTrials, NCT03156478; 17/05/2017), which was carried out in the primary health care system as part of the routine actions within three regions in Finland, including North Savo, South Karelia, and Päijät-Häme. The study protocol, clinical measurements, interventions and recruitment process of this RCT have been described in detail previously^13,14,17^. The StopDia study was approved by the Research Ethics Committee of the Hospital District of Northern Savo (statement number: 467/2016). Written informed consent to participate in the study was obtained from all participants. All experiments were performed in accordance with relevant guidelines and regulations.

The participants were recruited using the Stop Diabetes Digital Screening Tool, which included a validated Finnish Diabetes Risk Score (FINDRISC)^15^. Several communication channels were used for recruitment that was implemented between March 2017 and February 2018^17^. The inclusion criteria for the study were (1) age of 18–70 years; (2) 12 points or more in the FINDRISC or previous gestational diabetes or repeated impaired fasting glucose or impaired glucose tolerance; (3) living in one of the three regions (North Savo, South Karelia, and Päijät-Häme); (4) possibility to use computer, smartphone or tablet with internet connection; (5) having a phone number of own; and (6) adequate Finnish language skill. Exclusion criteria were (1) type 1 or 2 diabetes (if type 2 diabetes was diagnosed during the study, a person was included in the baseline analyses of the present study); (2) pregnancy or breastfeeding; and (3) current cancer or less than 6 months from the end of cancer treatment.

Eligible individuals were invited to participate in the study and book an appointment with a study nurse at their local primary healthcare centre. At the first study visit, the participants signed a written informed consent form, and the nurse performed clinical measurements and referred the participants to laboratory measurements. After the first study visit, the participants received a personal link by email to the StopDia Digital Questionnaire and were instructed to complete the questionnaire within two weeks. Physical activity was assessed with questions of conditioning and everyday physical activity as well as physical activity during work trips. Adherence to a recommended diet and diet preventing type 2 diabetes were assessed using the Healthy Diet Index^18^. The participants who met the inclusion criteria, had filled out the StopDia Digital Questionnaire, had given blood samples in a local laboratory, and had no diabetes according to the results of the 2-hour oral glucose tolerance test (OGTT) were randomly assigned to a digital intervention group (DIGI), a combined digital and group-based face-to-face intervention group (DIGI + F2F), or a control group (CONTROL) with 1:1:1 allocation. The control group was not included in this metabolomics study. The participants for the metabolomics study (*N* = 624) were randomly chosen except for the following conditions: all participants classified into T2D group were chosen; the participant was excluded if the plasma sample or clinical data entry was missing from either baseline or 1-year time point.

The digital intervention involved the use of an application available for portable devices, which allowed for user-chosen behavioral suggestions (out of 489 total suggestions), and support for habit formation with prompts for daily self-monitoring and provision of summary feedback^13^. The behavioural suggestions were related to improving meal frequency, the intake of various food groups, physical activity, sedentary behaviour, sleep, mood, and non-smoking. The face-to-face activities contained group coaching complementing the digital habit formation strategies with motivational, problem solving, and peer support components. Adherence to the lifestyle intervention was monitored by the application.

In total, a subset of 624 individuals from the StopDia study population^14^ was included in the metabolomics analyses. Out of them, 455 individuals meeting the criteria for prediabetes, participating in the lifestyle intervention, had their 1-year samples analysed, whereas 169 individuals diagnosed with type 2 diabetes only had their baseline measurements taken. Supplemental Figure S1 shows the baseline subgroups and the intervention groups of the StopDia study that were included in these analyses. For the one-year intervention analysis, the participants were categorised into three groups according to the American Diabetes Association criteria for the diagnosis of diabetes 14: normal glucose tolerance (NGT; *N* = 153), isolated impaired glucose tolerance (IGT; *N* = 87), and impaired glucose tolerance with increased fasting glucose (IGT + IFG; *N* = 216)^16^. Because the primary outcome of the StopDia study only showed an effect when the two intervention groups were treated as one, in this metabolomics study we were especially interested in the plasma metabolites that were altered during the intervention in those individuals at increased type 2 diabetes risk. Therefore, we focused on the two intervention groups and performed metabolomics only on the baseline and one-year samples of the two groups.

### LC-MS analysis

The plasma samples were prepared for the metabolomics analysis according to Klåvus et al.^19^. Briefly, cold acetonitrile was added in a ratio of 400 µL per 100 µL of plasma into filter plates (Captiva ND filter plate 0.2 μm) and mixed by pipetting. The samples were then centrifuged for 5 min at 700 × *g* at 4 °C and kept at 10 °C until analysis. The quality control (QC) samples were prepared by pooling aliquots from the experimental samples.

An ultra-high performance liquid chromatography (LC) system (Vanquish Flex UHPLC, Thermo Scientific, Bremen, Germany) was used for the analysis, which was coupled online to a high-resolution mass spectrometry (MS, Q Exactive Classic, Thermo Scientific). The samples were measured using two distinct chromatographic techniques: reversed phase (RP) and hydrophilic interaction chromatography (HILIC). The analyses in RP include the utilization of both electrospray ionization (ESI) polarities, which were ESI positive (ESI+) and ESI negative (ESI−). Only ESI + data was collected in HILIC. The centroid mode was used to obtain a full scan data. The data was collected over a mass to charge ratio (*m/z*) range of 120 to 1200 in the RP technique and 75 to 750 in HILIC technique. To identify metabolites, we acquired data-dependent product ion spectra (MS/MS) from pooled quality control (QC) samples at the beginning and end of the analysis for each mode. In addition, QC samples were incorporated in the analysis at the beginning and subsequently after every 12 samples. The configuration and specifications for the LC-MS instrument have been previously published^20^.

### Data analysis

The signal detection and alignment were performed in MS-DIAL version 4.48^21^ according to Klåvus et al.^19^. Briefly, the minimum peak height for the mass spectrometry data was set at 200 000 counts, and for the alignment, retention time tolerance was set at 0.6 min for HILIC data and 0.5 min for RP data. A sample-to-blank filter was used to remove the solvent background by requiring that the ratio of the maximum peak height in the samples and the average peak height in the solvent blank injections was more than 5. The *gap filling by compulsion* option was used to reduce missing values in the data by re-extracting signal intensities at expected *m/z* and retention time positions where a feature was detected in other samples. The data matrix containing the aligned peak areas across all samples was then pre-processed in *notame* R package^19^ to correct the drift in signal intensity across the QC samples and analytical batches and to filter out poor-quality molecular features. The number of molecular features after each data processing step is presented in Supplemental Table S1. The *k*-means clustering analysis was performed with Multiple Experiment Viewer 4.9.0.

### Statistical analyses

The differential molecular features between the NGT, isolated IGT, IGT + IFG, and T2D groups at baseline were determined with the Kruskal–Wallis test. Multivariable linear mixed-effects models (LME) were applied to determine the contribution of each molecular feature on the changes in clinical outcome variables (Supplemental Table S2) during the intervention in DIGI and DIGI + F2F. The covariate *p*-value from the model signifies whether there was a statistically significant association between the molecular feature and the clinical outcome variable, whereas the time * group interaction *p*-value indicates how the intervention modified the association between the molecular features and the clinical outcomes. Both models were adjusted for age and sex and repeated-measures structure was taken into account with the mixed model using the participant as subject for random effects. Benjamini–Hochberg false discovery rate correction was performed on the *p-*values (reported as *q*-values) to account for the potential false positive results caused by multiple measurements. The principal component analysis (PCA) was generated with the *prcomp* function in R version 4.3.3., with variables centered and scaled to unit variance. Features with zero variance were excluded prior to analysis. The resulting PCA scores were visualized using the *ggbiplot* package and the density curves with the *cowplot* package. The PERMANOVA analysis was performed on the good-quality molecular features using *vegan* package in R.

### Metabolite identification

The differential metabolites between the baseline groups and altered by the intervention were identified with MS-DIAL by comparing the observed MS/MS spectra with our in-house spectral database (level 1), publicly available spectral databases, such as MassBank, ReSpect, GNPS, RIKEN, and HMDB (level 2), and MS/MS spectra generated in silico in MS-FINDER 3.50^22^ (level 3). The level of the annotation reliability is based on the Metabolomics Standards Initiative (MSI) recommendations^23^.

## Results

### Differences among baseline groups

Table 1 shows the baseline characteristics of the participants included in this metabolomics study. Of the 624 participants including those with type 2 diabetes, 72% were women, 29% were people with overweight (BMI 25–30), and 64% were people with obesity BMI > 30. The mean age (SD) was 57.5 (8.8) years and the mean BMI (SD) was 32.3 (5.5) kg/m^2^. At baseline, 153 participants (25%) were classified as having normal glucose tolerance (NGT), whereas 87 (14%) had isolated impaired glucose tolerance (IGT), 215 (34%) had impaired glucose tolerance with increased fasting glucose (IGT + IFG), and 169 (27%) had type 2 diabetes.

**Table 1:** Baseline characteristics of the participants included in the metabolomics analyses: all participants with baseline measurements and participants in the intervention group (all participants except those in the T2D group).

	All participants	Intervention group	NGT	IGT	IGT + IFG	T2D
	(*N* = 624)	(*N* = 455)	(*N* = 153)	(*N* = 87)	(*N* = 215)	(*N* = 169)
Sex (female)	450 (72%)	342 (75%)	116 (76%)	71 (82%)	155 (72%)	108 (64%)
Age (years)	57.5 (8.8)	57.6 (9.0)	58.6 (8.8)	56.9 (9.0)	57.3 (9.0)	57.1 (8.1)
40–65 years	482 (77%)	344 (76%)	103 (67%)	70 (80%)	171 (80%)	138 (82%)
Study region						
North Savo	146 (23%)	105 (23%)	28 (18%)	15 (17%)	62 (29%)	41 (24%)
South Karelia	195 (31%)	136 (30%)	28 (18%)	23 (26%)	85 (40%)	59 (35%)
Päijät-Häme	283 (45%)	214 (47%)	97 (63%)	49 (56%)	68 (32%)	69 (41%)
Body mass index (kg/m^2^)	32.3 (5.5)	31.5 (5.3)	30.1 (4.8)	31.6 (5.7)	32.5 (5.2)	34.2 (5.7)
< 25.0	42 (6.7%)	38 (8.4%)	18 (12%)	10 (11%)	10 (4.7%)	4 (2.4%)
25.0–29.9	183 (29%)	147 (32%)	62 (41%)	25 (29%)	60 (28%)	36 (21%)
≥ 30.0	399 (64%)	270 (59%)	73 (48%)	52 (60%)	145 (67%)	129 (76%)
Glucose metabolism markers						
Fasting plasma glucose (mmol/L)	6.0 (1.2)	5.6 (0.6)	5.1 (0.3)	5.2 (0.3)	6.1 (0.4)	7.1 (1.7)
OGTT plasma glucose 120 min (mmol/L)	9.0 (3.2)	7.8 (1.9)	5.5 (1.0)	8.7 (0.7)	9.1 (1.0)	12.3 (3.7)
Blood HbA1c (mmol/mol)	38.8 (6.7)	36.9 (4.3)	34.6 (3.4)	36.2 (3.4)	38.9 (4.2)	43.7 (9.1)
FINDRISC total score (range 0–26)	17.0 (3.6)	16.4 (3.5)	14.9 (3.2)	16.0 (3.2)	17.5 (3.5)	18.9 (3.1)

First, we studied the differences in the metabolic profiles among the four baseline groups with variable glucose metabolism (NGT, isolated IGT, IGT + IFG, and T2D). The differences in the overall molecular features were visible already in the principal component analysis (PCA) (Supplemental Figure S2), where a gradual shift from the NGT group to T2D group via the IGT and IGT + IFG groups can be observed from the 95% confidence intervals; however, these differences in the molecular features representing the whole metabolome were not statistically significant in the PERMANOVA analysis (*p* = 0.085). When the metabolic profiles of those individuals that underwent the DIGI or DIGI + F2F intervention were compared between the baseline and one-year time points, a negligible difference was observed based on the confidence intervals and the density plots (Supplemental Figure S2).

**Fig. 1:**
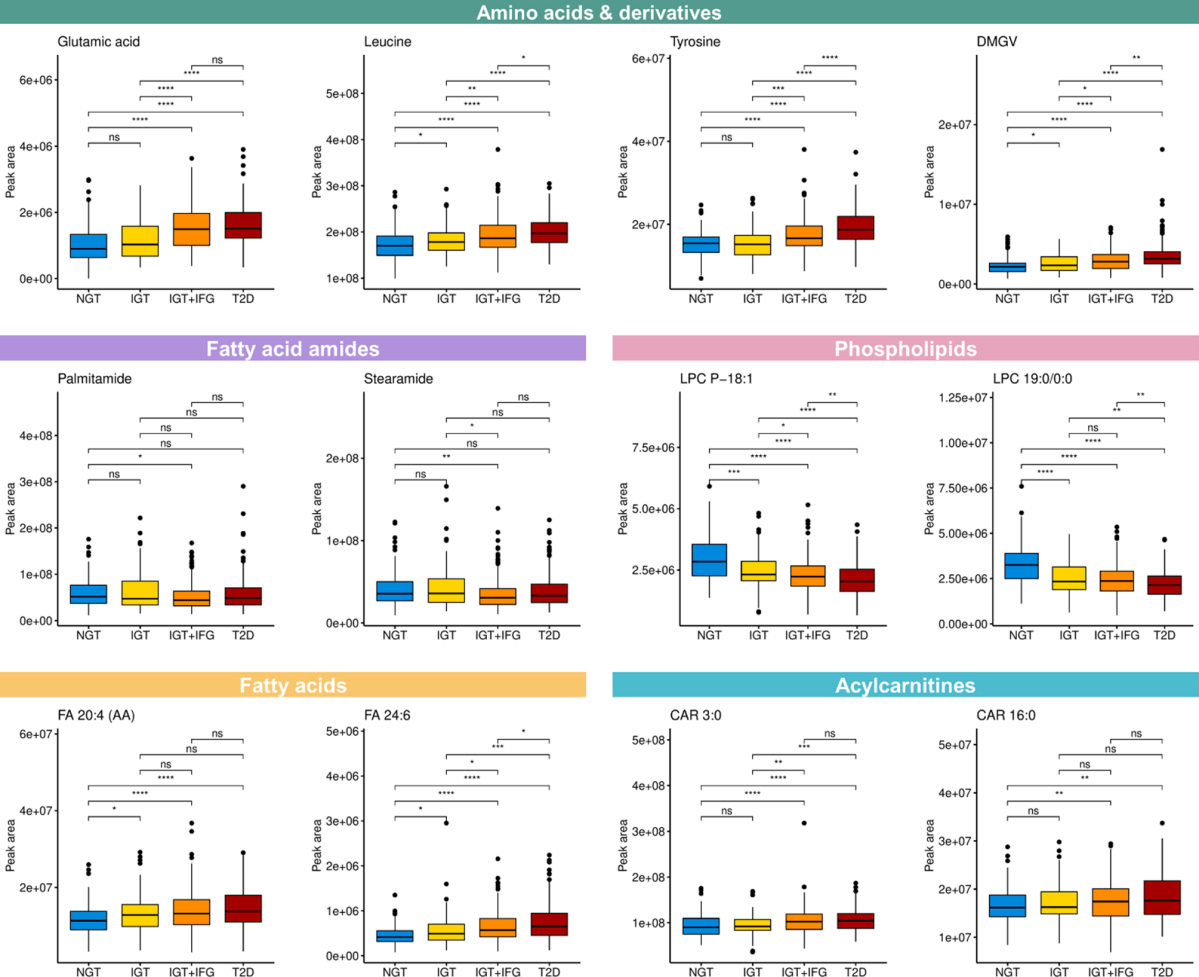
Levels of selected metabolites, representing various metabolite classes, in the baseline groups at baseline. Wilcoxon tests were performed between the group pairs; ns: not significant; *: *p* < 0.05; **: *p* < 0.01; ***: *p* < 0.001; ****: *p* < 0.0001. Metabolite abbreviations: DMGV: dimethylguanidinovaleric acid; LPC: lysophosphatidylcholine; P: plasmalogen; FA: fatty acid; AA: arachidonic acid; CAR: acylcarnitine. NGT: normal glucose tolerance, IGT: isolated impaired glucose tolerance, IGT + IFG: impaired glucose tolerance with increased fasting glucose, T2D: type 2 diabetes.

We then identified metabolites that were different among the baseline groups. Out of the 149 metabolites identified in this study, 113 were statistically significantly different (*q* < 0.05) across the NGT, isolated IGT, IGT + IFG, and T2D groups at baseline (Supplemental Table S3). Many of these metabolites exhibit associations among the baseline groups ranging from participants with normal glucose metabolism to those with type 2 diabetes, the metabolites having gradually higher or lower levels in groups with a metabolic status closer to type 2 diabetes (Fig. 1). Differential metabolites with lower levels in the T2D group compared to NGT include lysophosphatidylcholines (LPCs) with odd-chain fatty acid chains (e.g., LPC 17:0 and LPC 19:0), plasmalogens (e.g., LPC P-16:0), alkyl ether phosphatidylcholines (e.g., LPC O-18:1), glycerophosphocholine (GPC), *alpha*-tocopherol, glutamine, trigonelline, hippuric acid, pyrocatechol sulfate, and fatty acid amides (stearamide and palmitamide) (Supplemental Table S3). We detected four isomers of LPC 17:0, all behaving similarly across the four baseline groups; the isomers were determined using our in-house database of reference standards to differentiate based on the location and chain type (straight or branched-chain) of the fatty acid moiety (Supplemental Figure S3). Metabolites with higher levels in the T2D group compared to the NGT group include amino acids and their derivatives (e.g., glutamic acid, isoleucine, leucine, phenylalanine, tyrosine, and valine), polyunsaturated fatty acids (PUFAs; e.g., FA 24:6 and FA 22:4), dimethylguanidinovaleric acid (DMGV), phosphatidylcholines, acylcarnitines (short-, medium- and long-chain), and ursodeoxycholic acid. Figure 1 shows the levels of metabolites representing different metabolite classes that had differences between the baseline groups.

### Metabolic response to the intervention

**Fig. 2:**
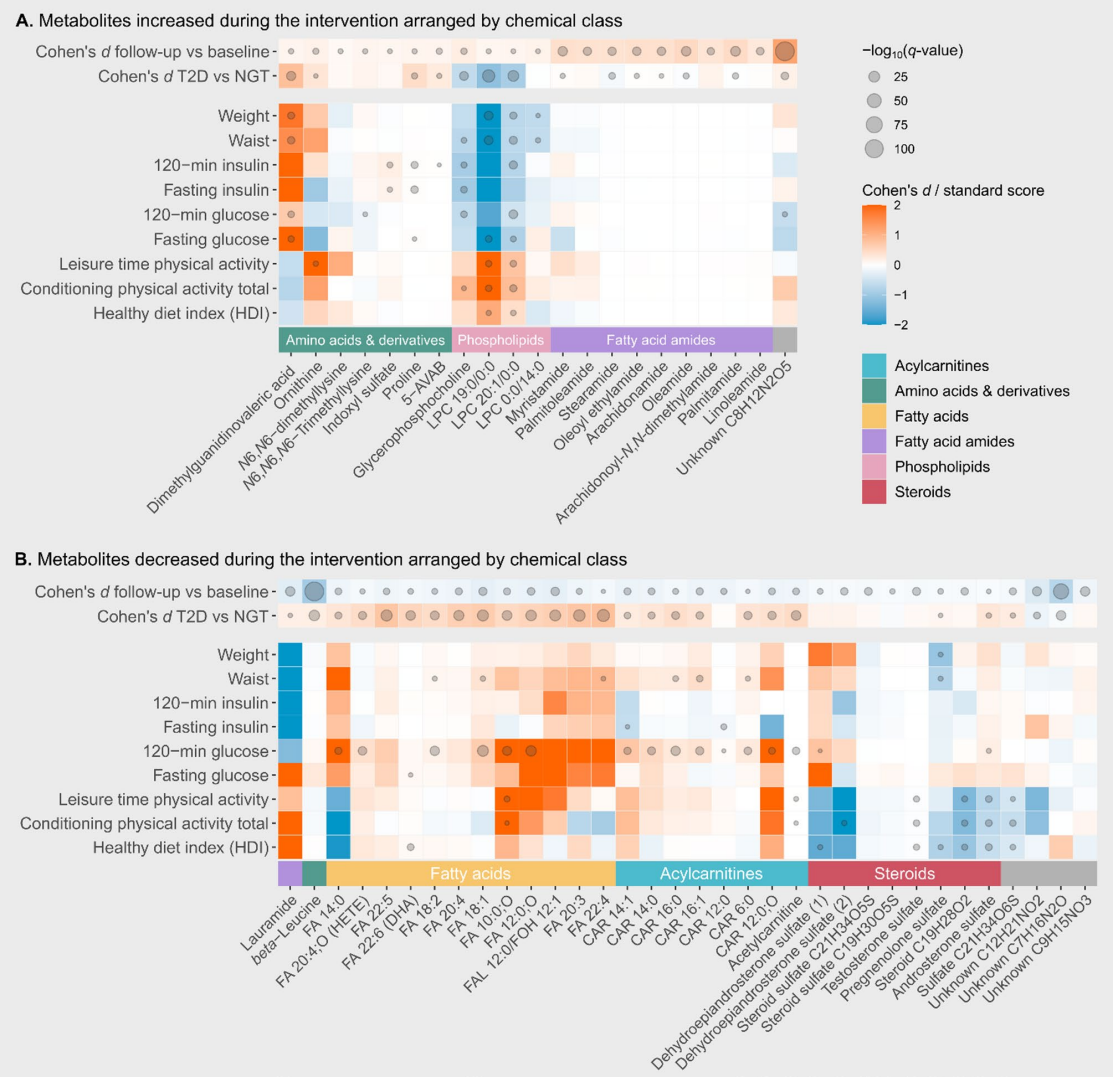
Heatmaps of the metabolites that increased (**A**) or decreased (**B**) during the intervention and their associations with clinical biomarkers. The Cohen’s *d* values are calculated for the difference between baseline and 1-year timepoint (all groups that underwent the intervention; a positive value indicates an increase during the intervention) and for the difference between the NGT and T2D groups at baseline, where a positive value indicates a higher metabolite level in the T2D group. The associations between the metabolites and the clinical biomarkers are represented as normalized standard scores of the covariates from the linear mixed model: a positive value indicates that the metabolite and the clinical biomarker changed in the same direction during the intervention. A cut-off value − 2…+2 was applied to all the values for the colour scale, and the metabolites were arranged based on their chemical class and hierarchical clustering.

Because StopDia was a trial on type 2 diabetes risk factors, our main interest was on the metabolites that in addition to their associations with baseline glucose metabolism could be modified by a lifestyle intervention. We performed multivariable linear mixed-effects models (LME) to study the impact of the intervention on the metabolite levels. Since the two intervention groups (digital application with and without face-to-face group counselling) performed very similarly regarding the change in clinical markers^14^, they were treated as one intervention group in these analyses. We annotated 55 metabolites that were differential between baseline and one-year timepoint (Fig. 2; Table 2). The 21 metabolites that increased during the intervention belong to three main metabolite classes: amino acids and derivatives, phospholipids, and fatty acid amides. The amino acid derivatives include DMGV, two *N*-methylated lysines, indoxyl sulfate, and 5-aminovaleric acid betaine (5-AVAB). The phospholipids include GPC and three lysophosphatidylcholines, whereas the nine fatty acid amides include oleamide, oleoyl ethylamide, and palmitamide.

**Table 2:** Metabolites impacted by the intervention based on the linear mixed model assessing the metabolite change over time (all groups merged; *n* = 55; *q* < 0.05).

Metabolite	Compound class	Cohen’s d	Linear mixed model; intervention effect	
		Baseline vs. 1-year	Regression coefficient	q-value
Unknown C_8_H_12_N_2_O_5_*		1.207	1,020,502	5.25E−111
*beta*-Leucine	Amino acid	−0.952	−3,257,685	7.30E−57
Unknown C_7_H_16_N_2_O		−0.739	−658,786	1.01E−33
Palmitamide	Fatty amide	0.465	18,189,250	1.02E−18
Oleamide	Fatty amide	0.488	47,466,726	2.59E−17
Myristamide	Fatty amide	0.410	393,114	9.08E−17
Arachidonamide	Fatty amide	0.404	29,133,680	4.35E−12
Oleoyl ethylamide	Fatty amide	0.417	16,543,994	5.99E−12
Stearamide	Fatty amide	0.376	9,402,731	2.23E−11
Palmitoleamide	Fatty amide	0.375	740,744	3.35E−11
Lauramide	Fatty amide	−0.319	−16,908	1.92E−09
Unknown C_9_H_15_NO_3_		−0.175	−346,013	2.25E−09
Linoleamide	Fatty amide	0.296	8,036,828	8.83E−09
Unknown C_12_H_21_NO_2_		−0.364	−240,132	1.43E−08
Arachidonoyl-*N*,*N*-dimethyl amide	Fatty amide	0.350	9,605,931	8.03E−08
FAL 12:0/FOH 12:1*	Fatty alcohol	−0.270	−102,189	3.13E−07
FA 18:1*	Fatty acid	−0.266	−564,006	2.52E−06
Testosterone sulfate	Steroid	−0.103	−5,354,664	3.59E−06
Steroid sulfate C_21_H_34_O_5_S	Steroid	−0.106	−625,771	1.20E−05
FA 12:0;O*	Fatty acid	−0.233	−133,066	2.24E−05
Steroid C_19_H_28_O_2_	Steroid	−0.102	−146,681	4.37E−05
Glycerophosphocholine*	Phosphocholine	0.227	290,604	4.39E−05
Dehydroepiandrosterone sulfate (isomer 2)	Steroid	−0.101	−60,813	5.07E−05
CAR 16:1*	Acylcarnitine	−0.199	−563,227	1.07E−04
5-AVAB	Betaine	0.142	10,722,019	1.33E−04
Sulfate C_21_H_34_O_6_S	Sulfate	−0.106	−347,109	1.45E−04
FA 20:4*	Fatty acid	−0.194	−948,185	1.47E−04
CAR 14:1*	Acylcarnitine	−0.202	−216,866	1.72E−04
Ornithine	Amino acid	0.172	63,345	2.14E−04
FA 10:0;O*	Fatty acid	−0.208	−99,095	2.20E−04
CAR 14:0*	Acylcarnitine	−0.193	−306,991	3.10E−04
Acetylcarnitine*	Acylcarnitine	−0.180	−51,331,026	4.02E−04
CAR 12:0;O*	Acylcarnitine	−0.191	−61,604	4.20E−04
FA 14:0*	Fatty acid	−0.179	−56,753	4.77E−04
FA 22:5*	Fatty acid	−0.168	−503,739	8.47E−04
CAR 12:0	Acylcarnitine	−0.195	−1,477,969	0.0020
FA 20:3*	Fatty acid	−0.170	−145,984	0.0034
CAR 16:0*	Acylcarnitine	−0.135	−543,029	0.0050
FA 18:2*	Fatty acid	−0.161	−1,212,779	0.0051
Dimethylguanidinovaleric acid (DMGV)*	Short-chain keto acid	0.127	188,625	0.0053
Dehydroepiandrosterone sulfate (isomer 1)	Steroid	−0.113	−61,122	0.0061
Proline	Amino acid	0.137	8,859,074	0.0076
*N*6,*N*6,*N*6-Trimethyllysine	Betaine	0.135	426,997	0.0078
FA 22:6 (DHA)*	Fatty acid	−0.132	−2,478,497	0.0082
Pregnenolone sulfate	Steroid	−0.098	−128,320	0.0083
*N*6,*N*6-dimethyllysine	Amino acid derivative	0.078	36,616	0.0106
PC 19:0/0:0 (LPC 19:0)*	Lysophosphatidylcholine	0.126	77,771	0.0120
Steroid sulfate C_19_H_30_O_5_S	Steroid	−0.089	−474,042	0.0137
FA 20:4;O (HETE)*	Oxylipin	−0.112	−346,223	0.0170
PC 20:1/0:0 (LPC 20:1)*	Lysophosphatidylcholine	0.127	279,954	0.0187
Indoxyl sulfate	Indole	0.125	1,034,015	0.0200
PC 0:0/14:0 (LPC 14:0)	Lysophosphatidylcholine	0.164	242,657	0.0211
Androsterone sulfate*	Steroid sulfate	−0.080	−264,051	0.0320
CAR 6:0*	Acylcarnitine	−0.120	−410,441	0.0409
FA 22:4*	Fatty acid	−0.126	−120,325	0.0450

The effect size is given as Cohen’s *d* value and regression coefficient of the linear mixed model, which corresponds with the absolute change in the signal abundance of the metabolite. Metabolites written in bold (*n* = 40) also had a difference (Kruskal–Wallis; *q* < 0.05) between the baseline groups at baseline. Metabolites marked with an asterisk (*n* = 25) were associated (linear mixed model; *q* < 0.05) with the change in 120-min glucose level.

We annotated 34 metabolites that decreased during the intervention. Apart from one fatty acid amide and one amino acid, these metabolites belong to other metabolite classes than those that increased during the intervention: free fatty acids, acylcarnitines, and steroids. In fatty acids, we found decreases mostly in PUFAs, such as FA 18:2 (linoleic acid), FA 20:4 (arachidonic acid), FA 22:4 (adrenic acid) and FA 22:6 (DHA). Acylcarnitines that decreased were mostly medium- and long-chain acylcarnitines, such as C12, C14 and C16. Among the decreased steroids in our study were dehydroepiandrosterone sulfate (DHEAS), testosterone sulfate, and androsterone sulfate. Out of the metabolites affected by the intervention, the levels of 40 metabolites were also different between the baseline groups at baseline (Fig. 2); out of these, acetylcarnitine, *beta*-leucine, CAR 12:0;O, DMGV, GPC, FA 22:6 (DHA), stearamide, and two unknown molecular features with formulas C_8_H_12_N_2_O_5_ and C_7_H_16_N_2_O were among the most significant annotated metabolites in both the Kruskal–Wallis test between baseline groups and the LME on the intervention effect.

### Associations with clinical markers

**Fig. 3:**
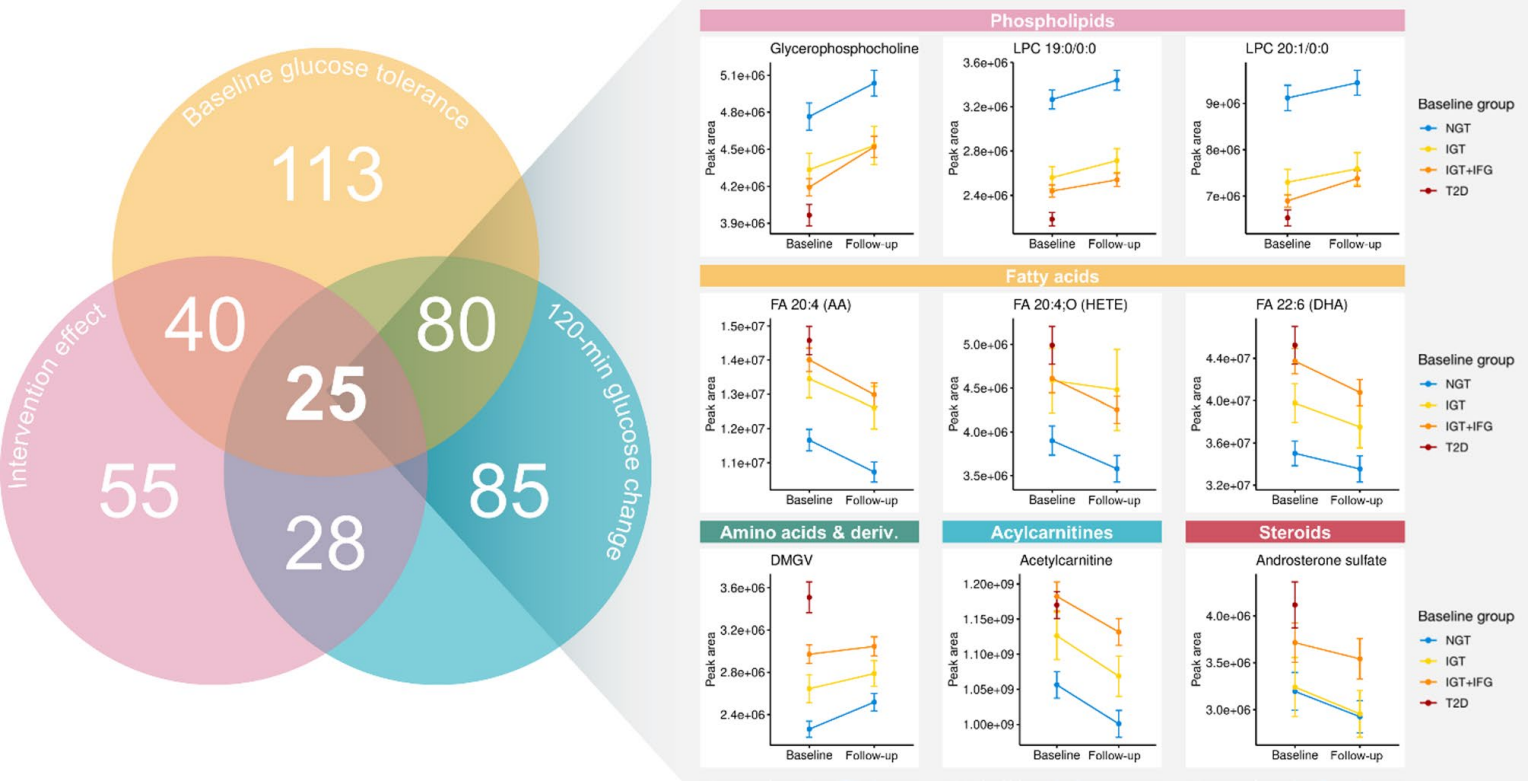
Venn diagram showing the numbers of annotated metabolites that differed across the four groups of glucose metabolism at baseline, that were changed during the one-year intervention period, and/or whose one-year changes correlated with a one-year change in 120-min glucose. Box plots show the baseline and one-year plasma levels of selected metabolites that were statistically significant in all these three statistical analyses in the four groups of glucose metabolism at baseline. Box plots of all the 55 metabolites impacted by the intervention are presented in Supplemental Figure S6. NGT: normal glucose tolerance, IGT: isolated impaired glucose tolerance, IGT + IFG: impaired glucose tolerance with increased fasting glucose, T2D: type 2 diabetes.

Finally, we explored whether the identified metabolites, both associating with baseline glucose metabolism and responsive to the lifestyle intervention, differed in their correlations with the change in weight, waist circumference, glucose or insulin metabolism, the Healthy Diet Index (HDI), and physical activity. These results are summarized in Fig. 2. Out of the 40 metabolites that were different between baseline groups and were altered by the intervention, 25 also were associated (*q* < 0.05) with the change in 120-min glucose (Fig. 3). Of the metabolites that increased during the intervention, DMGV positively correlated with weight, waist circumference, and fasting glucose, whereas LPC 19:0, a lysophosphatidylcholine containing an odd-chain fatty acid, correlated negatively with weight, waist circumference, and fasting glucose and positively with physical activity and HDI. We did not observe a significant impact of the intervention on LPC 17:0. Of the metabolites that decreased during the intervention, several fatty acids and all acylcarnitines correlated positively with the 120-min glucose, and the steroids tended to correlate negatively with physical activity and HDI.

DMGV exhibited an unexpected behaviour during the intervention: it increased in response to the intervention and positively correlated with weight change even though weight did not change significantly during the intervention. Therefore, to investigate the potential differences in individual responses to the intervention, we divided the individuals based on the increase/decrease of DMGV levels and plotted them based on weight change (Supplemental Figure S4). Compared to LPC 19:0, which responded to the intervention as expected, individuals with increased DMGV levels had a high number of those whose weight decreased but also several cases with a high weight increase. We further analyzed the two metabolites and two endpoint markers (weight and 120-min glucose, which correlated with the metabolites) in more detail using *k*-means clustering. This resulted in a heatmap showing several subgroups of individuals with unique responses in the metabolite levels and endpoint markers unrelated to the baseline group (Supplemental Figure S5).

## Discussion

This study showed that a metabolite signature that differs according to glucose metabolism can be used to advance the detection of abnormal glucose metabolism in individuals considered to be at increased risk for type 2 diabetes in a primary care setting even before traditional markers show adverse changes. Second, we found that some of these metabolites respond to a lifestyle intervention in a real-world setting that could be used in stratifying subgroups (“metabotypes”) that benefit from the intervention, as demonstrated by the individual responses of certain metabolites on the intervention, such as DMGV. Some of these metabolites are known to be related to the risk of type 2 diabetes, such as fatty acids, acylcarnitines, and lysophosphatidylcholines, whereas others are novel candidates to be explored further, such as fatty acid amides. On the other hand, several metabolites previously associated with the risk of type 2 diabetes, such as BCAAs and branched-chain fatty acids (BCFAs), did not respond to the intervention in an expected way. This suggests that either the amino acid metabolic pathways related to type 2 diabetes development were not affected by the lifestyle intervention or the effect was hindered by individual variation in the responses, as shown for DMGV.

Our first main finding was that certain BCAAs, aromatic amino acids, acylcarnitines, GPC, lysophosphatidylcholines containing odd-chain fatty acids and DMGV differed across the groups of glucose metabolism at baseline. Our results agree with the results of previous studies which have shown the associations of BCAAs (such as isoleucine and leucine), aromatic amino acids (such as tyrosine and phenylalanine), and short-chain acylcarnitines (such as C2, C3 and C6), with increased risk of type 2 diabetes^3,8^. GPC is an intermediate of glycerophospholipid metabolism whose metabolite linoleoylglycerophosphocholine (LGPC) has been found to be associated with decreased risk of type 2 diabetes^24^. It is also an osmolyte; lower plasma GPC levels in the T2D group can be a sign of disturbed cellular osmoregulation caused by high extracellular glucose levels, and the increase in plasma GPC levels during the intervention may indicate an improvement in the body fluid balance and osmolality^25,26^. Furthermore, several phospholipid species containing odd-chain fatty acids 15:0, 15:1, 17:0 and 19:0 have been inversely associated with the development of type 2 diabetes in previous studies^9,27^. It remains unclear how lipids containing odd-chain fatty acids are involved in the pathways of type 2 diabetes development. One hypothesis is that odd-chain fatty acids could improve mitochondrial function by providing intermediates for the tricarboxylic acid cycle^28^.

It is important to note that most of the metabolites that are associated with abnormal glucose metabolism are potentially regulated by lifestyle. We found the most significant increases in response to the lifestyle intervention in fatty acid amides, phospholipids, and amino acids and their derivatives. Fatty acid amides are a group of lipids that have been increasingly studied in recent years for their physiological and pharmacological effects, including regulation of the sleep/wake cycle and cell signalling via multiple receptors, such as the cannabinoid receptors^29,30^, which in turn are involved in the development of diabetes^31^. Recently, several fatty acid amides were identified as mediators of better exercise performance within the gut–brain axis, promoting dopamine levels in brain areas related to reward and pleasure processing^32^. Among phospholipids, GPC, LPC 19:0, and LPC 20:1 were found to increase most significantly and they were also associated with better glucose metabolism, which is parallel with the findings from the Australian prospective study on the risk and prevalence of type 2 diabetes and cardiovascular disease^33^ and another Finnish study on the prevention of type 2 diabetes^9^. Interestingly, LPC 19:0 and 20:1 were also positively associated with the HDI^18^ as well as leisure time and time spent on physical exercise. Eicosenoic acid (FA 20:1) is a monounsaturated fatty acid found in several vegetable oils^34^, which could explain its association with HDI. Furthermore, habitual physical activity could induce adipose tissue lipolysis which increases the levels of circulating free fatty acids. Moderate-to-vigorous physical activity has been positively associated with eicosenoic acid in plasma phospholipids in pregnant women^35^. Furthermore, eicosenoic acid is part of a fatty acid signature associated with decreased risk of type 2 diabetes^36^ and lysophosphatidylethanolamine (20:1) has been observed to decrease during gestational diabetes and be associated with 120-min glucose^37^; thus, the increase in LPC 20:1 observed in this study, linked to the metabolism of eicosenoic acid, could refer to improved glucose metabolism.

Among individual metabolites that increased after the intervention were 5-AVAB, DMGV, proline, and ornithine. 5-AVAB, which belongs to amino acid-derived quaternary ammonium compounds, has been suggested to have protective effects on heart function and type 2 diabetes^38,39^. Plasma DMGV differed across the groups of glucose metabolism, with the T2D group having the highest levels. DMGV has been identified as a predictive marker of type 2 diabetes up to 12 years before its onset^40^ and associated with increased risk of developing cardiovascular disease in the future^41^. Proline has been linked with an increased risk of type 2 diabetes as well^42^. The unexpected increase in DMGV and proline levels during the intervention may point towards individual factors in response to the intervention, as evidenced by subgroups with varying responses to the levels of DMGV and associated endpoint markers. This suggests that there are metabotypes associated with the efficiency of the intervention (Supplemental Figure S4 and S5). Ornithine is an amino acid in the urea cycle that is metabolized by ornithine decarboxylase (ODC) into putrescine. Early diabetes exhibits kidney growth and a higher glomerular filtration rate (GFR), which is linked to increased ODC activity^43^. We suggest that the increased ornithine levels observed in this intervention study could be a sign of lowered ODC activity and reduced hyperfiltration related to the onset of type 2 diabetes; however, more studies involving GFR measurements are needed to confirm this hypothesis.

Among the metabolites that decreased after the lifestyle intervention most significantly were free fatty acids (mostly PUFAs), acylcarnitines, and steroids. The effects of PUFAs, especially arachidonic acid and DHA, in eicosanoid production and inflammation have been extensively studied; however, both have also been associated with increased insulin sensitivity^44^, and therefore their decrease in this study may point out towards individual responses to the intervention. The decrease in acylcarnitines was associated with a decrease in 120-min glucose. The accumulation of long-chain acylcarnitines has been suggested to reflect incomplete fatty acid oxidation, and especially increased concentrations of acylcarnitines C10–C14 have been found in individuals with type 2 diabetes^45^. A decrease in the long-chain acylcarnitines in this study could indicate improved mitochondrial function and lipid metabolism as a result of the intervention. Many of the decreased steroids in our study were their sulfated form and also inversely associated with physical activity. Higher serum DHEAS levels have been associated with decreased risk of type 2 diabetes in older men but not in women^46^. Our results are in line with the previous findings which have shown that physical activity decreases free testosterone, androstenedione and DHEAS levels in women^47^. These findings could be explained by the conversion of sulfated steroids into their active forms in target tissues^48^.

This study has several strengths. The intervention has been carried out in a real-world primary care setting, which offers a novel approach to investigate metabolomic associations occurring without controlled experiments. The study setting also allows for combining associations with baseline glucose metabolism with the response to lifestyle intervention. The large number of studied samples combined with extensive characterization of the untargeted metabolomics data enabled us to determine several metabolites that differed statistically between all the baseline groups, some of which have not been described earlier. The study also has limitations in the study setting and analytical methodology. Due to our main study question, we did not perform metabolomics on the control group samples, which prevented us from verifying the intervention effect in a controlled setting. The study was conducted in a Finnish population, known to be genetically homogenous and relatively distant from other European ethnicities, which limits the generalizability of the results to other populations. Some of the interesting molecular features could not be identified because of the limited availability of reference spectra, an inherent issue in non-targeted metabolomics.

In this study, we report a panel of metabolites that differentiated four groups of individuals with varying glucose metabolism. These metabolites, which include both known markers of type 2 diabetes and novel candidates, could be utilised as a biomarker pattern to predict the stage of type 2 diabetes onset earlier than what is possible with traditional glucose tolerance tests. Identifying the status of glucose metabolism from a single blood sample with a sensitive method, such as LC-MS metabolomics, could allow for a cost-effective and individual treatment plan, and similar approaches have already been implemented commercially^49^. A significant portion of the metabolites reported in this study, including amino acids, phospholipids, fatty acid amides, free fatty acids, acylcarnitines, and steroids, responded to the lifestyle intervention in a real-world type 2 diabetes prevention program. They suggest various metabolic changes during the intervention, such as reduced production of inflammation markers, improved lipid metabolism via mithocondrial β-oxidation, and increased steroid sulfatase activity. Further research on the novel candidate markers, such as fatty acid amides, is warranted. Several key amino acid markers of active type 2 diabetes did not respond to the intervention, suggesting that individual means for type 2 diabetes prevention may be necessary and that single metabolite markers may not be specific enough to reflect the preventive effects. Our findings suggest that the metabolites reported in this study indicate the most responsive metabolic pathways to be targeted by personalized lifestyle interventions to mitigate the risk of type 2 diabetes.

## Supplementary Information

Below is the link to the electronic supplementary material.

Supplementary Material 1

## Data Availability

The dataset generated during and analyzed in the current study is available in the B2SHARE repository (10.23728/b2share.04de8e6f764a49baaca536d1ede1d3ae).

## Abbreviations

5-AVAB: 5-Aminovaleric acid betaine
BCAA: Branched-chain amino acid
BCFA: Branched-chain fatty acid
DHA: Docosahexaenoic acid
DHEAS: Dehydroepiandrosterone sulphate
DMGV: Dimethylguanidinovaleric acid
FINDRISC: Finnish diabetes risk score
GFR: Glomerular filtration rate
GPC: Glycerophosphocholine
HDI: Healthy diet index
IFG: Increased fasting glucose
IGT: Isolated impaired glucose tolerance
LGPC: Linoleylglycerophosphocholine
LME: Linear mixed-effects models
LPC: Lysophosphatidylcholine
MASH: Metabolic dysfunction-associated steatohepatitis
MASLD: Metabolic dysfunction-associated steatotic liver disease
MSI: Metabolomics Standards Initiative
NGT: Normal glucose tolerance
ODC: Ornithine decarboxylase
OGTT: 2-hour oral glucose tolerance test
PUFA: polyunsaturated fatty acid
QC: Quality control
RCT: Randomized controlled trial
