# Supplementary material for: Metabolites associated with abnormal glucose metabolism responding to primary care lifestyle intervention
Source: Sci Rep. 2025 Nov 7;15:39093. doi: 10.1038/s41598-025-25749-z (PMC12594967; doi:10.1038/s41598-025-25749-z)

Supplemental material

**Supplemental Table S1.** The number of aligned molecular features detected in the three different analytical modes after background removal and *notame* pre-processing as well as the number of features containing MS/MS spectra and a tentative match against in-house and publicly available databases.

|  | HILIC+ | RP+ | RP− |
| --- | --- | --- | --- |
| Aligned molecular features | 6 613 | 20 665 | 7 896 |
| of which remaining after background removal | 6 155 | 19 277 | 7 466 |
| of which passed the quality metrics in *notame* | 1 210 | 3 045 | 2 012 |
| of which contain MS/MS spectra | 501 | 1 052 | 563 |
| of which have a tentative match in a database entry | 244 | 399 | 148 |

**Supplemental Table S2.** List of the outcome variables included in the multivariable mixed models. The Healthy Diet Index (HDI) is based on Lindström et al. 2021.

| Variable | Description |
| --- | --- |
| Weight | Change in body weight, kg |
| Waist | Change in waist circumference, cm |
| Conditioning physical activity total | Change in time spent on physical exercise, hours/d |
| Leisure time physical activity | Change in time spent on overall physical activity, hours/d |
| Fasting glucose | Change in fasting glucose, mmol/L |
| 120-min glucose | Change in glucose 120 min after glucose tolerance test, mmol/L |
| Fasting insulin | Change in fasting insulin, IU/mL |
| 120-min insulin | Change in insulin 120 min after glucose tolerance test, IU/mL |
| Healthy diet index | Change in healthy diet index (HDI) |

**Supplemental Table S3.** Metabolites with differences between the baseline groups at baseline (*n* = 79, Kruskal–Wallis *q* < 0.0001). Metabolites written in bold changed during the intervention (all groups considered). A positive Cohen’s *d* value signifies higher metabolite level in the type 2 diabetes (T2D) group compared to the normal glucose tolerance (NGT) group.

| **Curated ID** | **Compound class** | **Cohen’s *d* T2D vs NGT** | **Kruskal–Wallis  *q*-value** |
| --- | --- | --- | --- |
| **PC 19:0/0:0 (LPC 19:0)** | Lysophosphatidylcholine | −1.157 | 3.91E-36 |
| PC 17:0/0:0 (LPC 17:0; isomer 4) | Lysophosphatidylcholine | −1.162 | 1.88E-30 |
| LPC 17:0 (isomer 1) | Lysophosphatidylcholine | −1.184 | 8.75E-30 |
| PC 0:0/17:0 (LPC 17:0; isomer 3) | Lysophosphatidylcholine | −1.175 | 8.81E-29 |
| LPC P-18:1 | Plasmalogen | −1.043 | 9.48E-29 |
| PC 20:0/0:0 (LPC 20:0) | Lysophosphatidylcholine | −0.977 | 6.37E-28 |
| PC P-16:0/0:0 (LPC P-16:0) | Plasmalogen | −1.093 | 5.08E-27 |
| LPC 17:0 (isomer 2) | Lysophosphatidylcholine | −1.091 | 2.21E-26 |
| LPC O-18:1 | Alkyl ether phosphatidylcholine | −1.014 | 3.43E-26 |
| PC P-18:0/0:0 (LPC P-18:0) | Plasmalogen | −1.047 | 3.82E-26 |
| **PC 20:1/0:0 (LPC 20:1)** | Lysophosphatidylcholine | −0.907 | 6.33E-25 |
| Glutamic acid | Amino acid | 0.933 | 1.97E-24 |
| PC O-34:3 | Alkyl ether phosphatidylcholine | −0.902 | 6.69E-23 |
| PC 0:0/18:2 (LPC 18:2) | Lysophosphatidylcholine | −0.770 | 2.34E-22 |
| Tyrosine | Amino acid | 1.022 | 5.05E-22 |
| LPC O-16:0 | Alkyl ether phosphatidylcholine | −0.883 | 6.71E-22 |
| FA 24:6 | Fatty acid | 0.945 | 2.06E-21 |
| LPC O-18:0 | Alkyl ether phosphatidylcholine | −0.949 | 4.61E-21 |
| PC O-36:3 | Alkyl ether phosphatidylcholine | −0.741 | 8.01E-21 |
| PC 0:0/15:0 (LPC 15:0) | Lysophosphatidylcholine | −0.900 | 1.44E-19 |
| PC 18:1/0:0 (LPC 18:1) | Lysophosphatidylcholine | −0.754 | 2.53E-19 |
| PC 15:0/0:0 (LPC 15:0) | Lysophosphatidylcholine | −0.906 | 7.93E-19 |
| **FA 22:4** | Fatty acid | 0.818 | 1.92E-18 |
| Isoleucine | Amino acid | 0.876 | 1.54E-17 |
| Leucine | Amino acid | 0.807 | 2.50E-16 |
| PC 20:2/0:0 (LPC 20:2) | Lysophosphatidylcholine | −0.606 | 2.81E-16 |
| PC 18:2/17:0 | Phosphatidylcholine | −0.737 | 2.94E-16 |
| **FA 20:3** | Fatty acid | 0.746 | 4.37E-16 |
| Acylcarnitine C_12_H_23_NO_4_ | Acylcarnitine | 0.623 | 5.11E-16 |
| LPC 18:0 | Phosphatidylcholine | −0.720 | 8.37E-16 |
| PC 37:6 | Phosphatidylcholine | 0.774 | 2.07E-15 |
| **Dimethylguanidinovaleric acid (DMGV)** | Short-chain keto acid | 0.833 | 2.41E-15 |
| PC 18:0/0:0 (LPC 18:0) | Lysophosphatidylcholine | −0.717 | 5.26E-15 |
| **FA 22:5** | Fatty acid | 0.750 | 1.26E-14 |
| **FA 18:1** | Fatty acid | 0.656 | 2.10E-14 |
| **FAL 12:0/FOH 12:1** | Fatty alcohol | 0.624 | 8.31E-14 |
| **Glycerophosphocholine** | Phosphocholine | −0.642 | 1.13E-13 |
| CAR 4:0 | Acylcarnitine | 0.369 | 1.37E-13 |
| PC 0:0/17:1 (LPC 17:1) | Lysophosphatidylcholine | −0.593 | 6.21E-13 |
| **FA 12:0;O** | Fatty acid | 0.563 | 7.02E-13 |
| ***beta*-Leucine** | Amino acid | 0.219 | 9.36E-13 |
| FA 18:1 | Fatty acid | 0.624 | 4.21E-12 |
| CAR 6:1 | Acylcarnitine | 0.620 | 5.66E-12 |
| PC 40:8 | Phosphatidylcholine | −0.546 | 5.73E-12 |
| Valine | Amino acid | 0.689 | 1.21E-11 |
| PC 17:1/0:0 (LPC 17:1) | Lysophosphatidylcholine | −0.631 | 1.31E-11 |
| **FA 20:4** | Fatty acid | 0.617 | 2.51E-11 |
| Cyclo(Leu-Pro) | Cyclic peptide | 0.571 | 5.48E-11 |
| PC O-40:8 | Alkyl ether phosphatidylcholine | −0.690 | 2.98E-10 |
| **FA 10:0;O** | Fatty acid | 0.479 | 3.07E-10 |
| PC 32:1 | Phosphatidylcholine | 0.664 | 4.26E-10 |
| **Acetylcarnitine** | Acylcarnitine | 0.473 | 6.39E-10 |
| **FA 22:6 (DHA)** | Fatty acid | 0.528 | 8.53E-10 |
| **Unknown C_7_H_16_N_2_O** |  | −0.073 | 9.21E-10 |
| Phenylalanine | Amino acid | 0.659 | 1.07E-09 |
| LPC 16:0 | Lysophosphatidylcholine | −0.541 | 1.33E-09 |
| LPE 18:0 | Lysophosphatidylethanolamine | −0.496 | 2.45E-09 |
| **Unknown C_8_H_12_N_2_O_5_** |  | 0.176 | 1.27E-08 |
| Alanine | Amino acid | 0.510 | 2.64E-08 |
| **FA 18:2** | Fatty acid | 0.454 | 4.07E-08 |
| CAR 3:0 | Acylcarnitine | 0.523 | 5.08E-08 |
| PC 18:1/22:6 | Phosphatidylcholine | −0.611 | 2.68E-07 |
| **CAR 12:0;O** | Acylcarnitine | 0.387 | 9.07E-07 |
| Trigonelline | Betaine | −0.295 | 1.68E-06 |
| **CAR 6:0** | Acylcarnitine | 0.341 | 2.06E-06 |
| **FA 20:4;O (HETE)** | Oxylipin | 0.441 | 2.24E-06 |
| Carnitine | Betaine | 0.402 | 3.34E-06 |
| Oleoylethanolamide | Fatty amide | 0.328 | 3.91E-06 |
| **CAR 16:0** | Acylcarnitine | 0.421 | 5.67E-06 |
| **Stearamide** | Fatty amide | −0.110 | 5.76E-06 |
| Pyrocatechol sulfate | Phenyl sulfate | −0.283 | 1.03E-05 |
| CAR 8:1 | Acylcarnitine | 0.240 | 1.14E-05 |
| *alpha*-Tocopherol | Vitamin E | −0.455 | 1.96E-05 |
| **CAR 16:1** | Acylcarnitine | 0.345 | 2.99E-05 |
| Ursodeoxycholic acid | Bile acid | 0.460 | 4.78E-05 |
| Hippuric acid | Phenolic metabolite | −0.285 | 5.97E-05 |
| **Palmitamide** | Fatty amide | −0.026 | 8.86E-05 |
| Glutamine | Amino acid | −0.422 | 9.46E-05 |


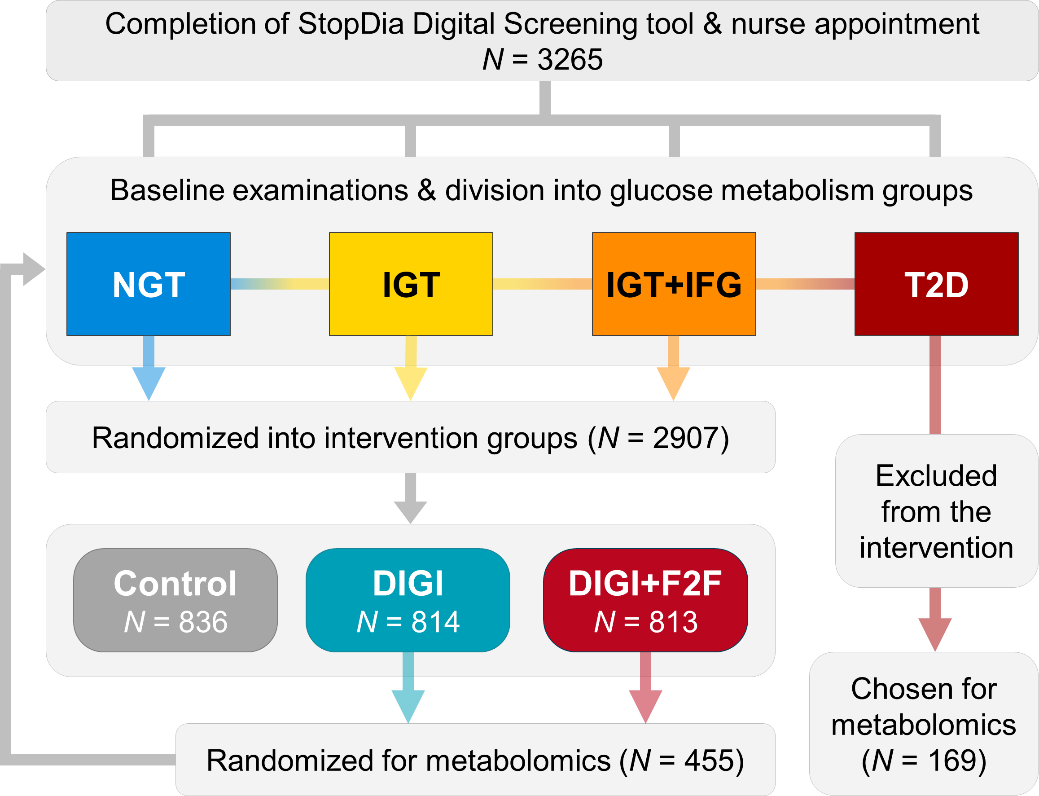


**Supplemental Figure S1.** The design of the StopDia metabolomics study. NGT: normal glucose tolerance, IGT: isolated impaired glucose tolerance, IGT+IFG: impaired glucose tolerance with increased fasting glucose, T2D: type 2 diabetes, DIGI: digital lifestyle intervention, DIGI+F2F: digital lifestyle intervention and face-to-face councelling. The complete flowchart of the StopDia study has been published previously (8).

| **A**  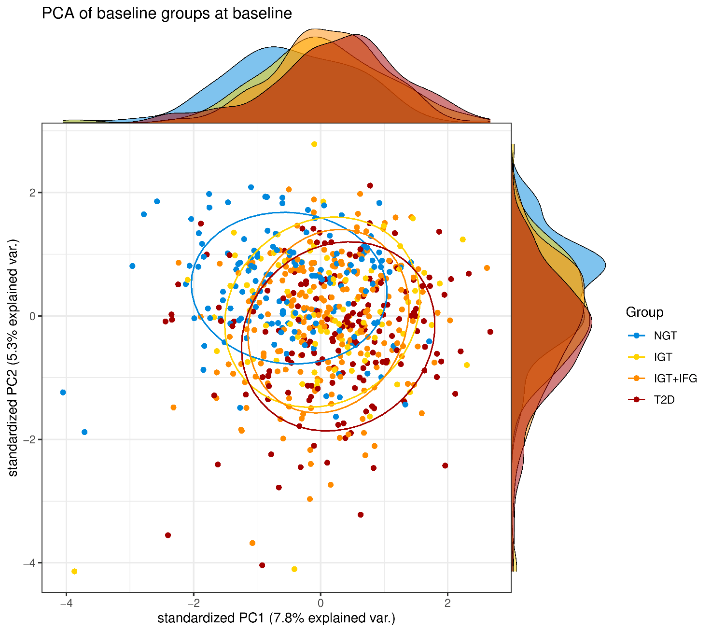 | **B**  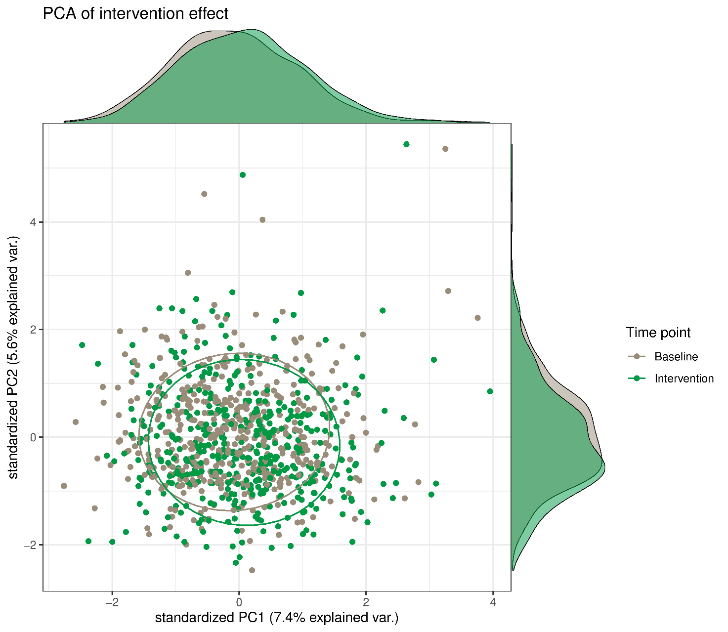 |
| --- | --- |

**Supplemental Figure S2.** Principal component analysis of A) the samples from the four baseline groups with variable glucose tolerance at baseline and B) the samples from the individuals participating in the intervention at baseline and after the intervention.


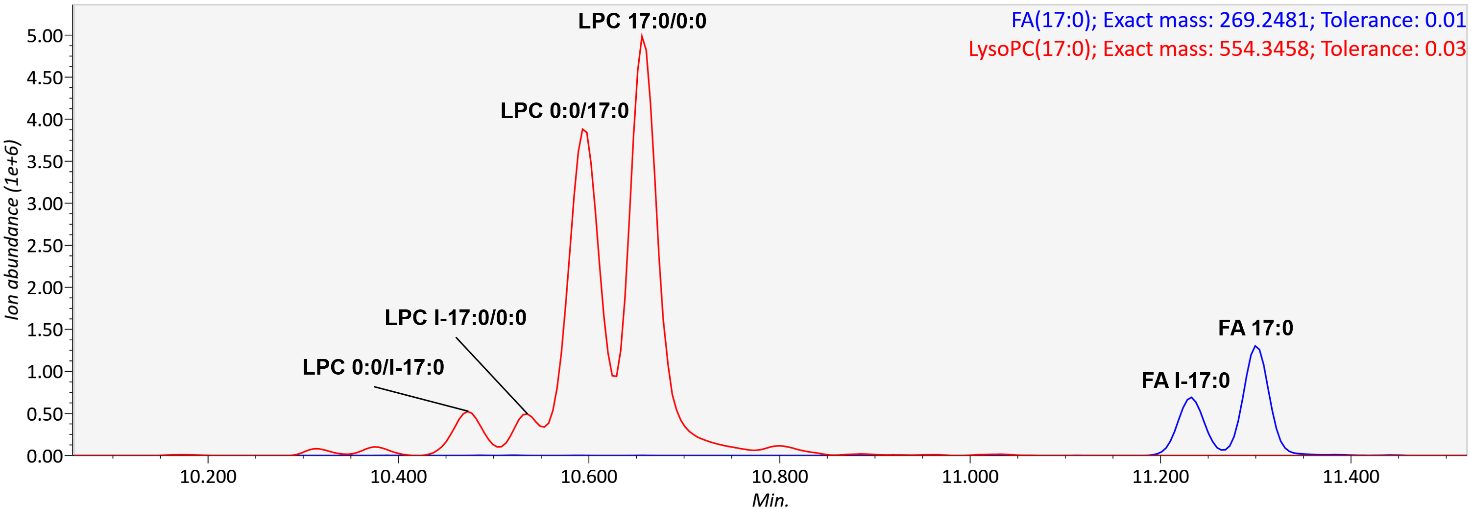


**Supplemental Figure S3.** An extracted ion chromatogram (EIC) of the isomeric peaks of LPC 17:0 and FA 17:0 in a pooled quality control (QC) sample. Four peaks of LPC 17:0 were characterized, the two eluting first containing a branched-chain FA 17:0 moiety in *sn*-2 and *sn*-1 position, followed by compounds containing the straight-chain FA 17:0 moiety in *sn*-2 and *sn*-1 position. The fatty acid FA 17:0 itself is also separated into two peaks according to the branching of the acyl chain. The exact location of the chain branching could not be determined in this analysis. Image: MS-DIAL ver 4.48.


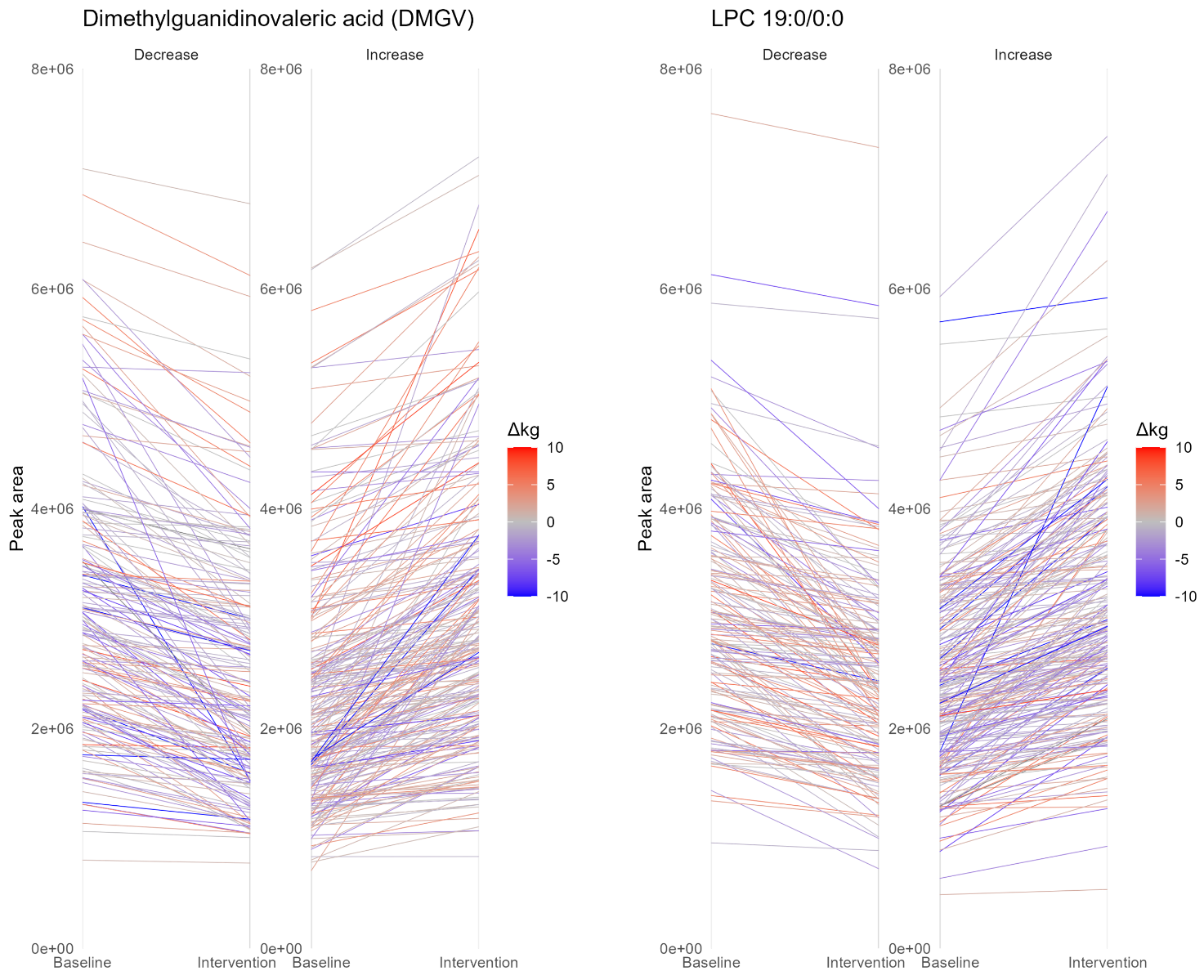


**Supplemental Figure S4.** Line plots of dimethylguanidinovaleric acid (DMGV) and LPC 19:0/0:0 divided into individuals with a decrease (left panel) and increase (right panel) in the metabolite levels. The lines are colour-coded based on the weight change during the intervention.

**
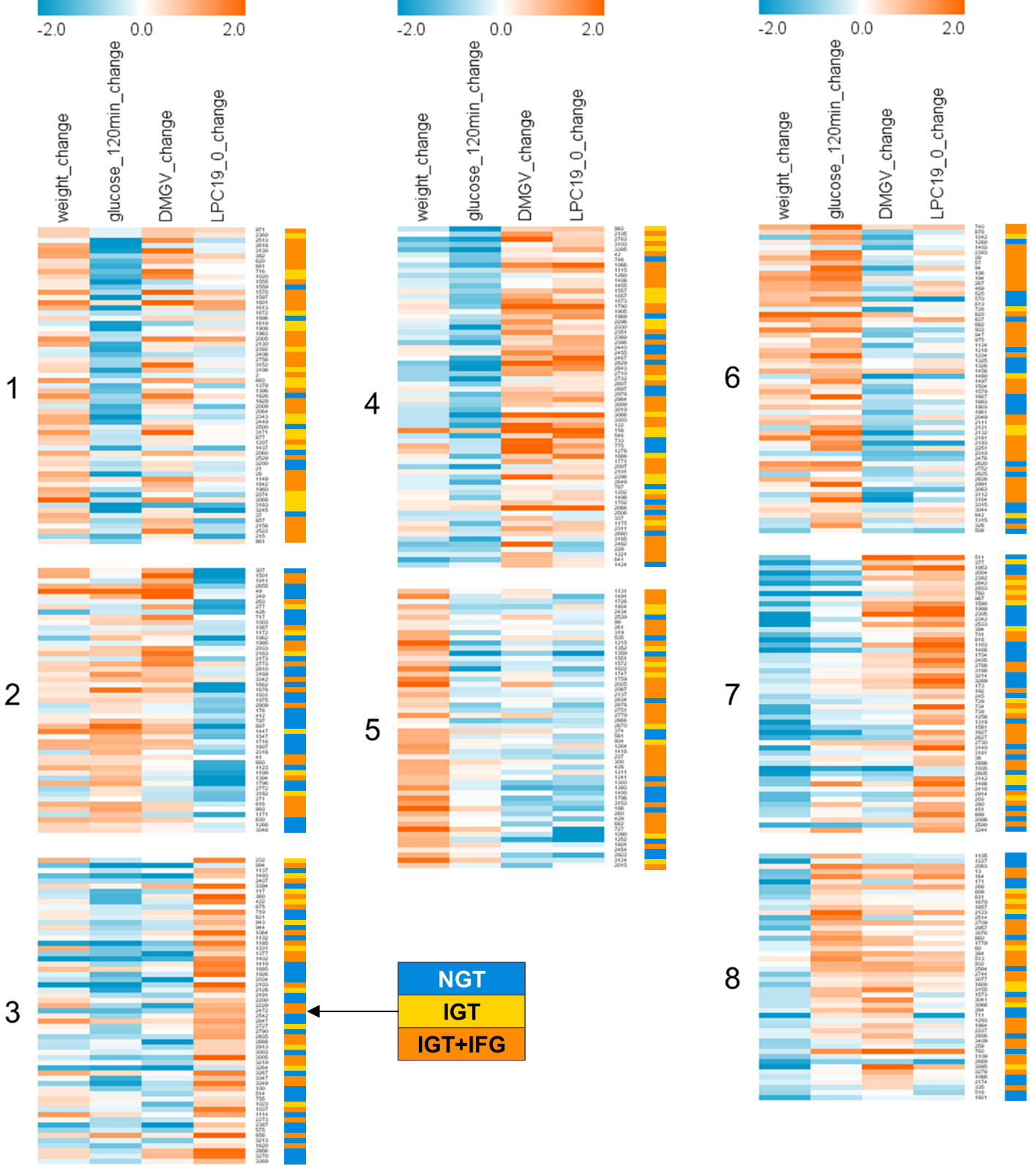
**

**Supplemental Figure S5.** Heatmap of the changes (intervention – baseline) in weight, 120-min glucose, DMGV and LPC 19:0/0:0. *k*-Means clustering was applied to the data and the number of clusters was adjusted to yield clusters with distinct patterns. Standardization was performed on each column to unify the ranges: each value was divided by the standard deviation of the column to keep positive values as positive and negative values as negative. The baseline group (NGT, IGT, or IGT+IFG) is shown in a separate panel.

**Supplemental Figure S6 (below).** Box plots of the 55 metabolites impacted by the intervention in the linear mixed-effects model.


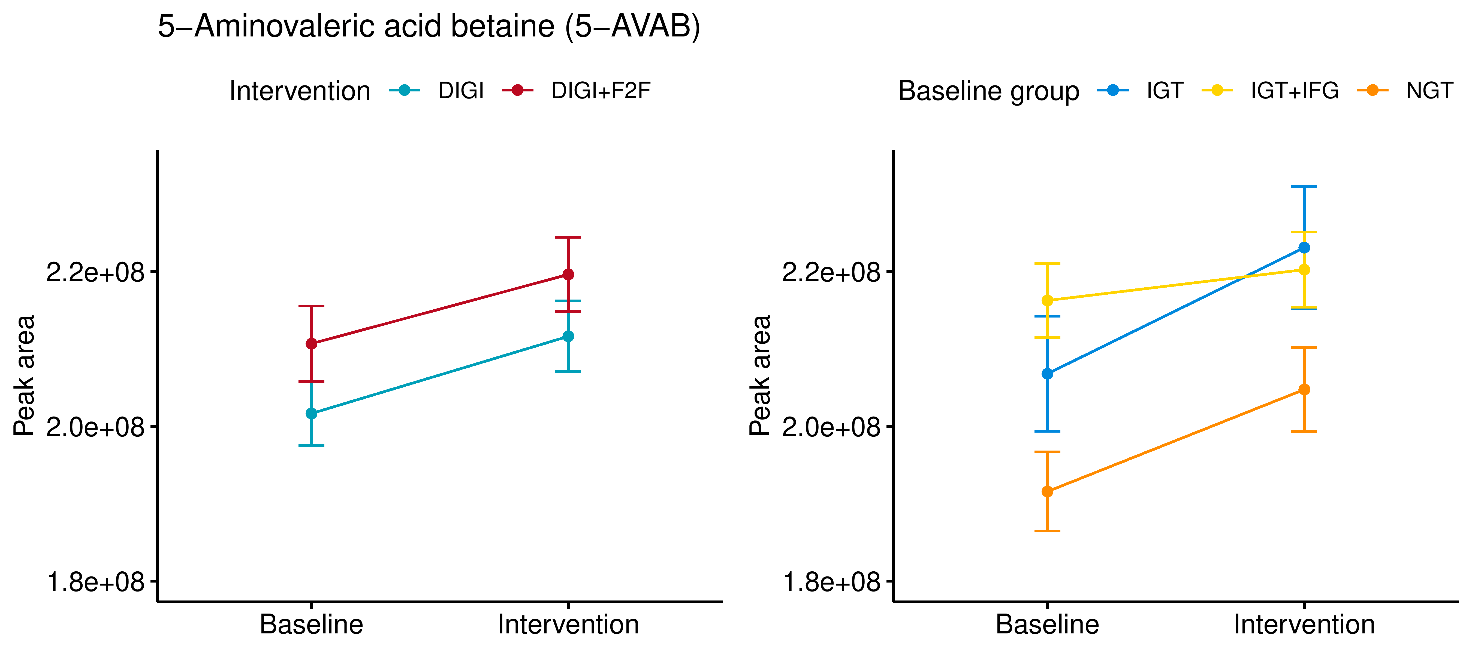

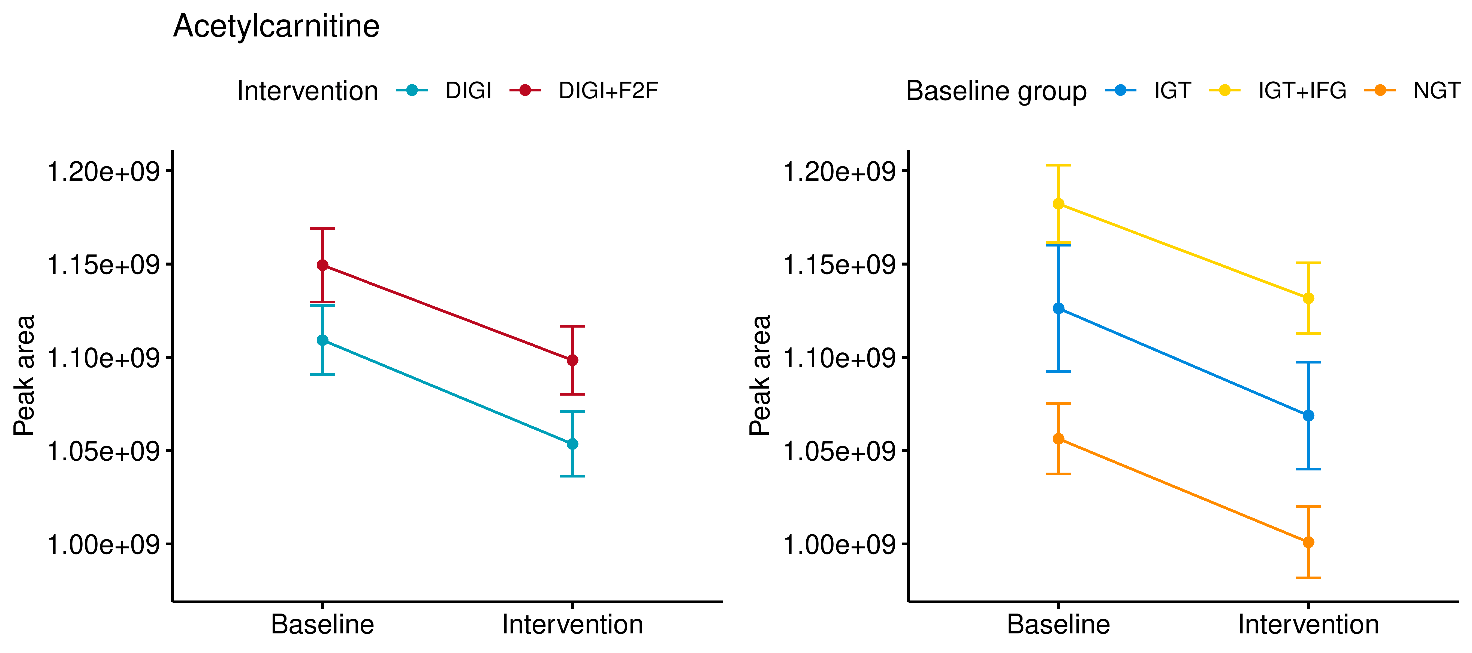

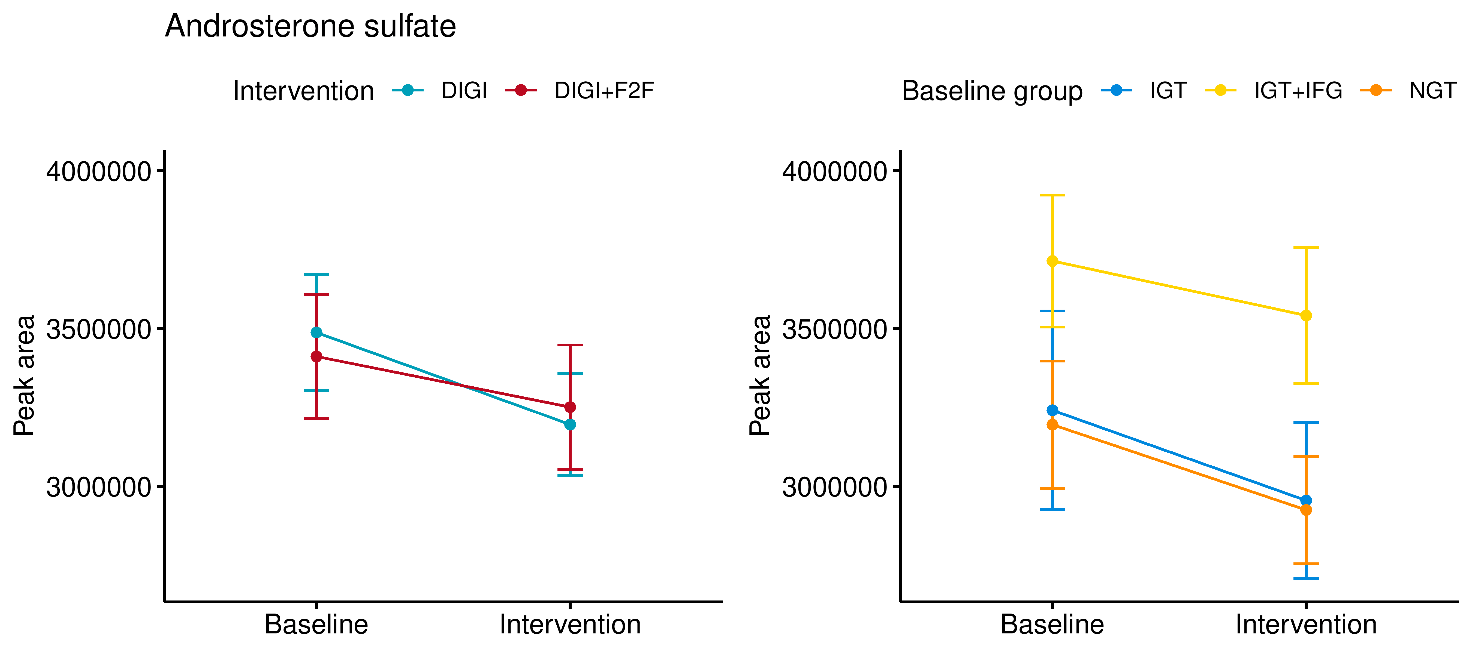

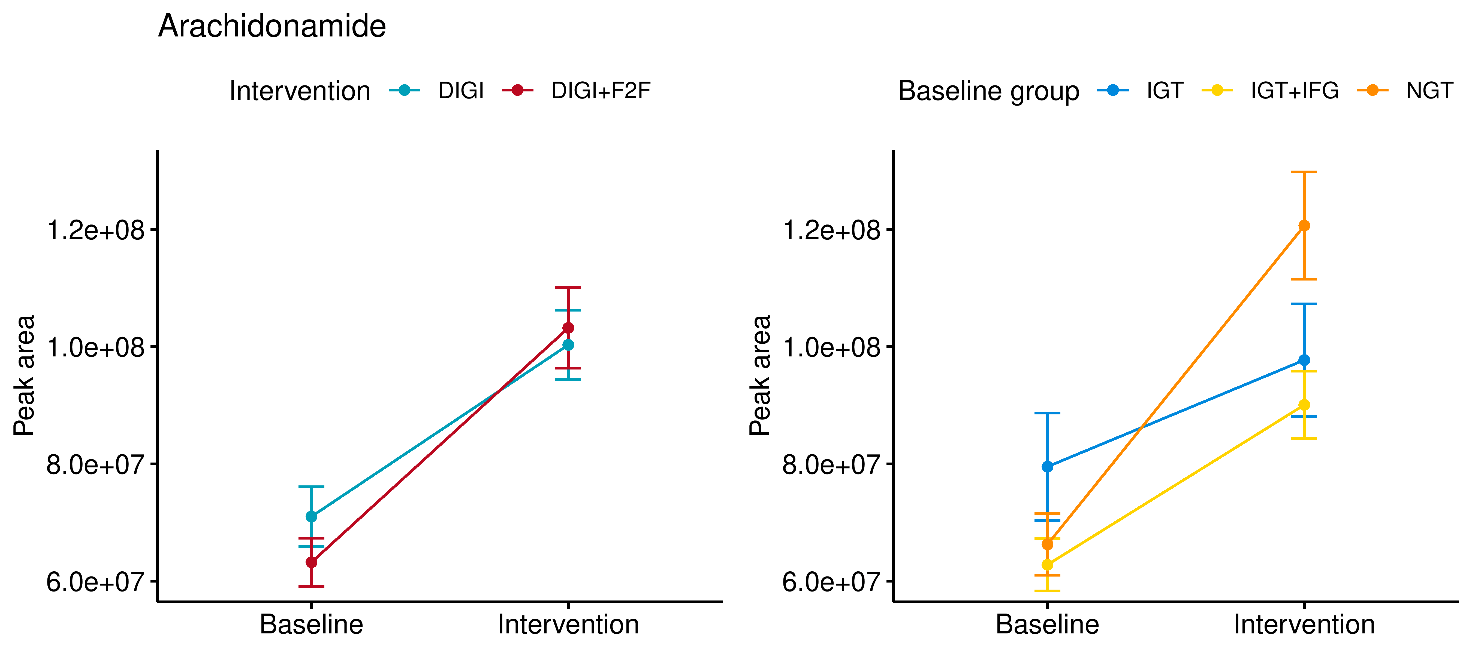

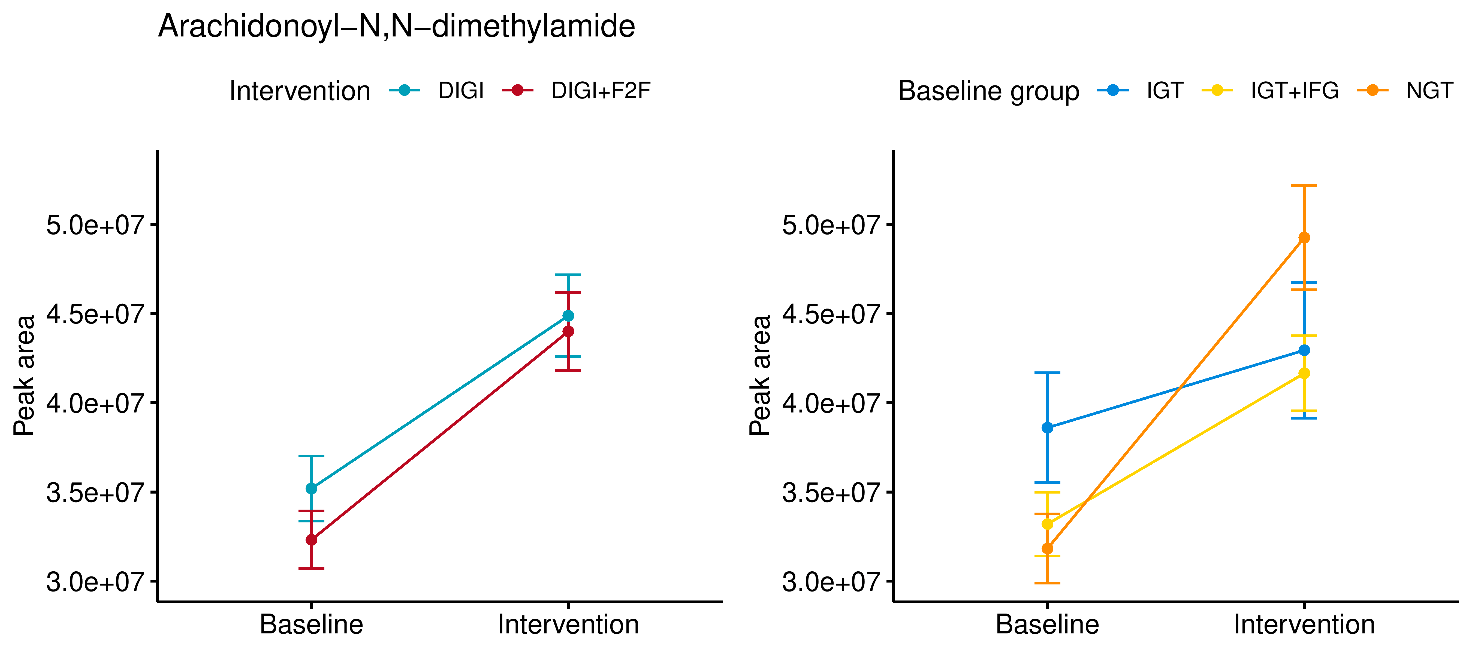

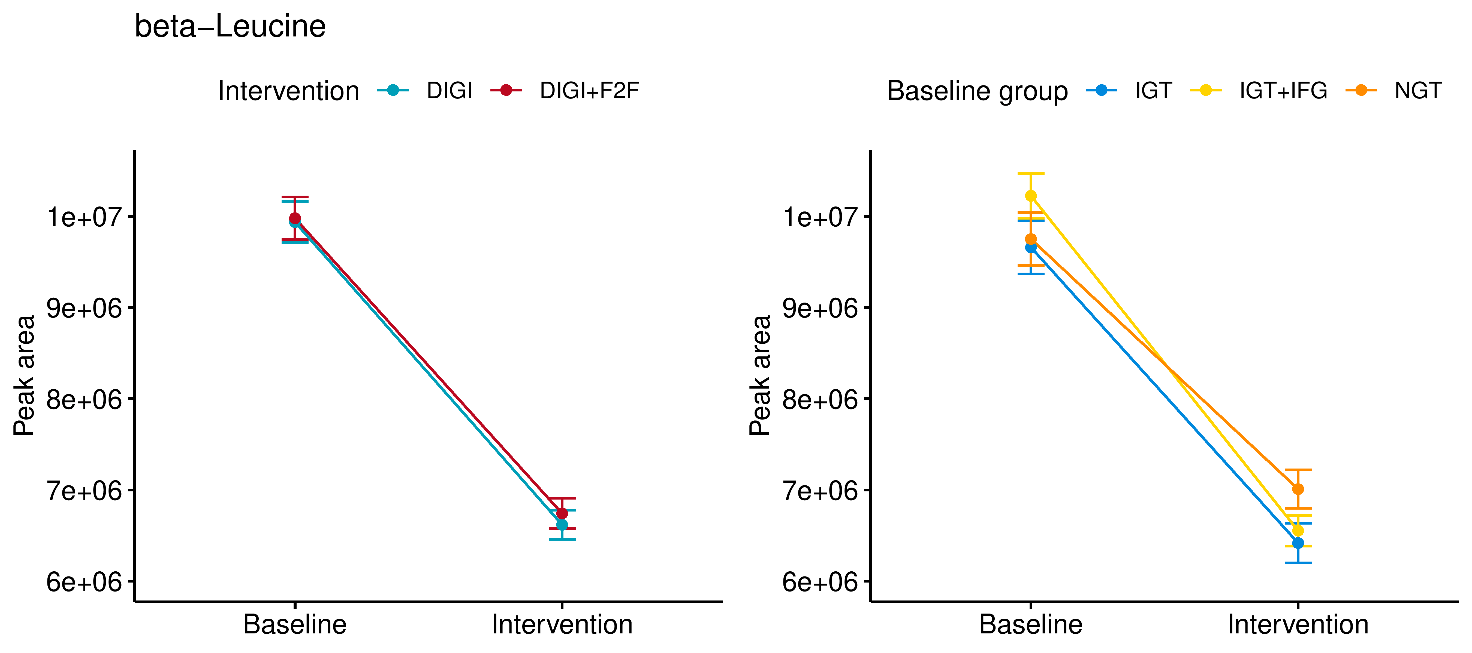

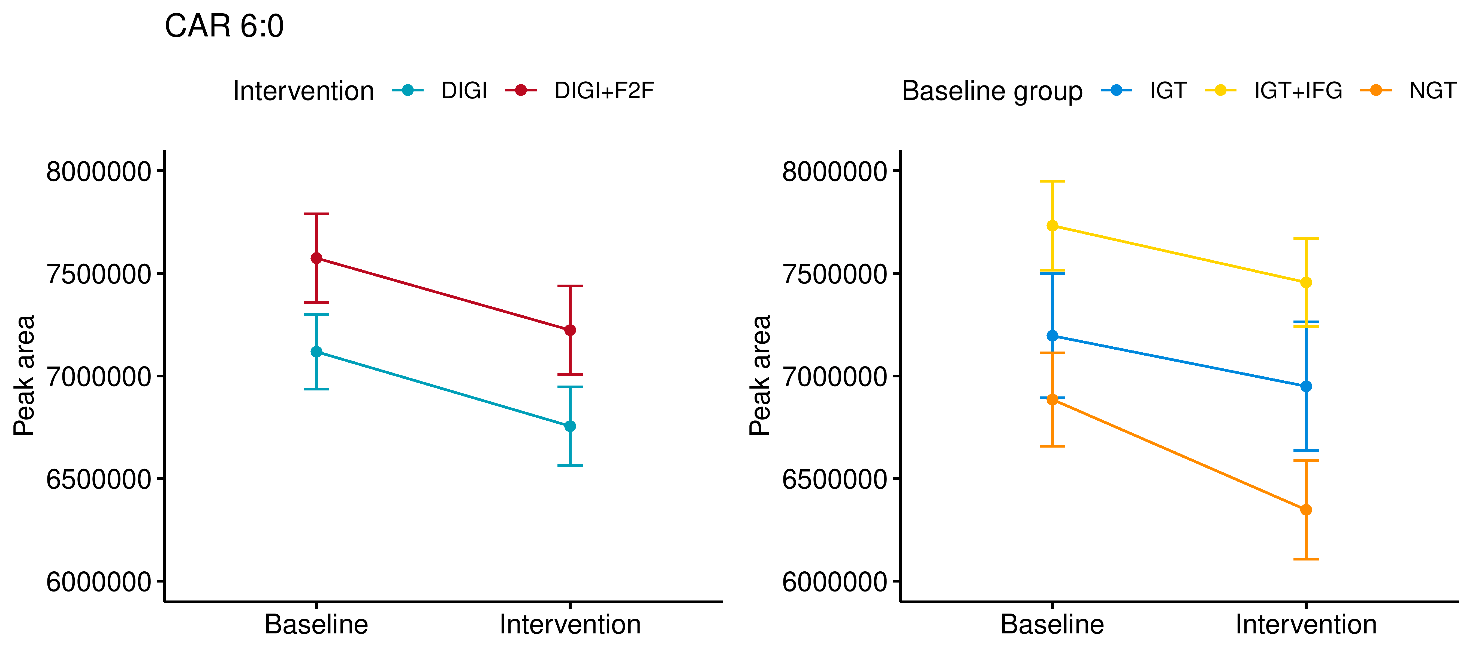

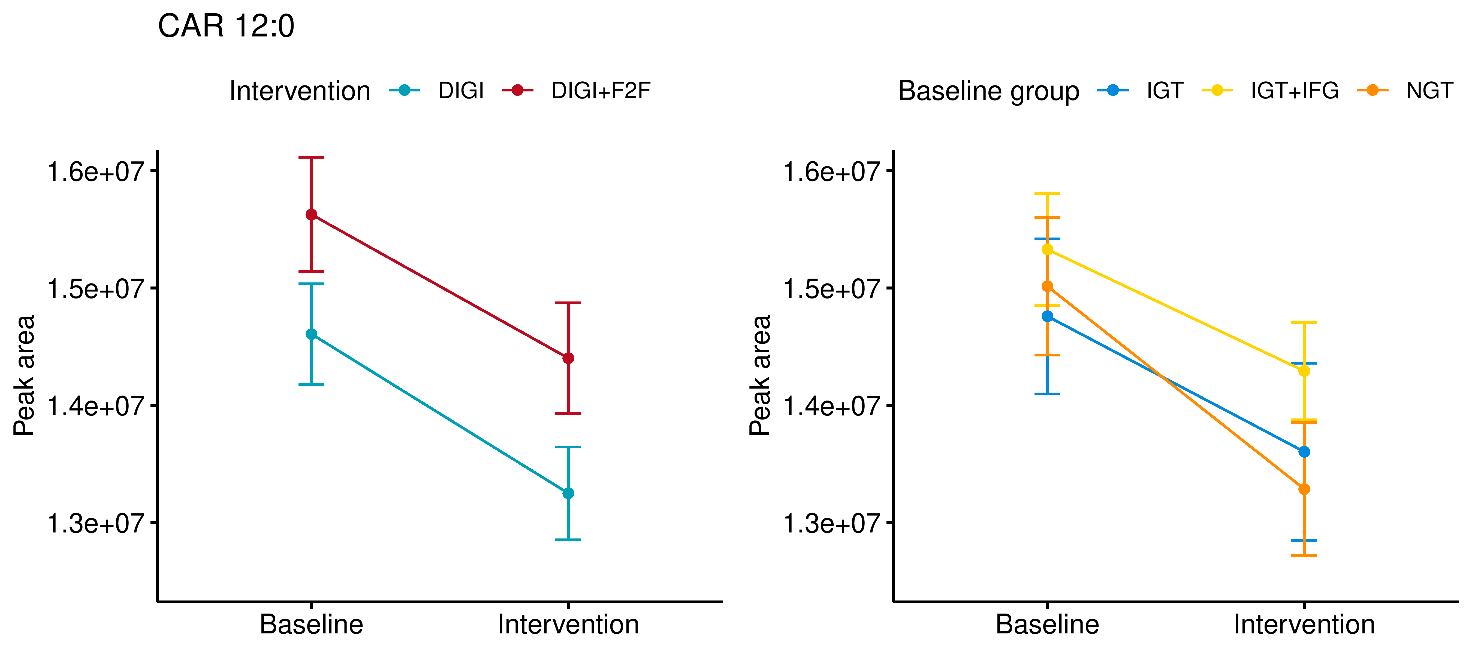

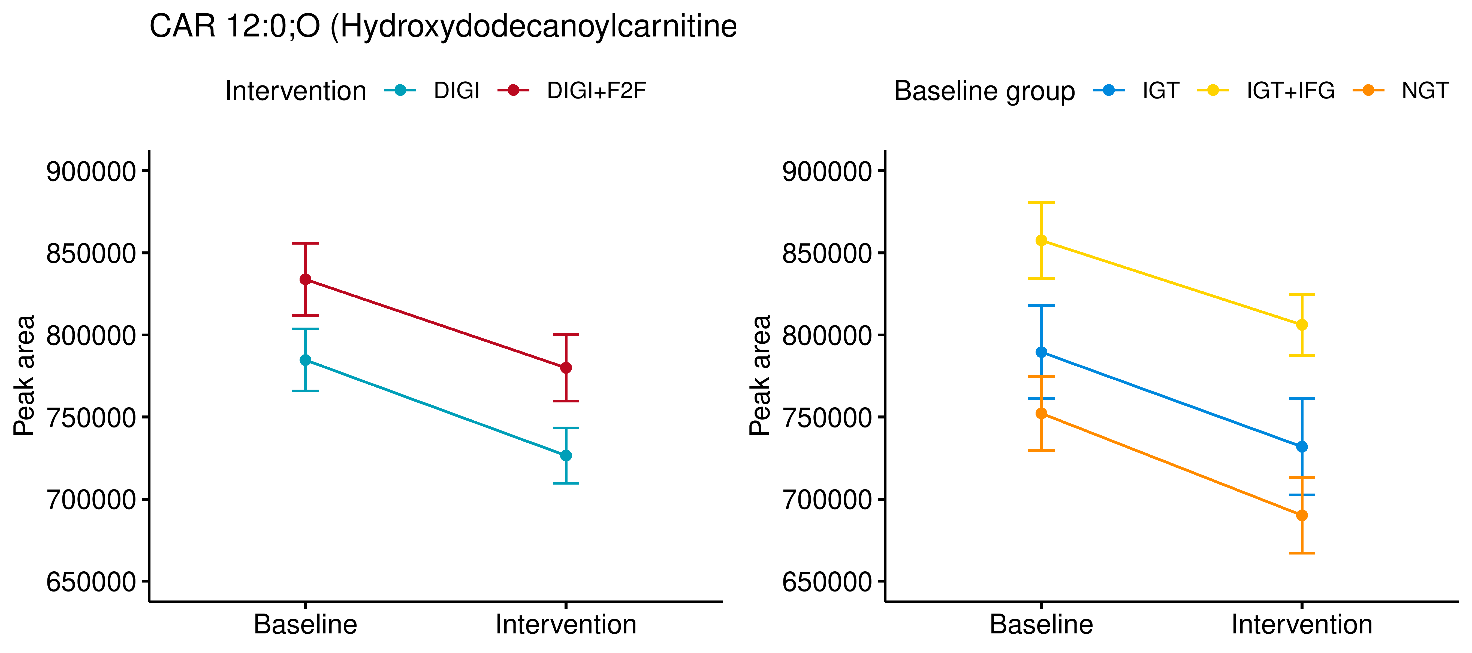

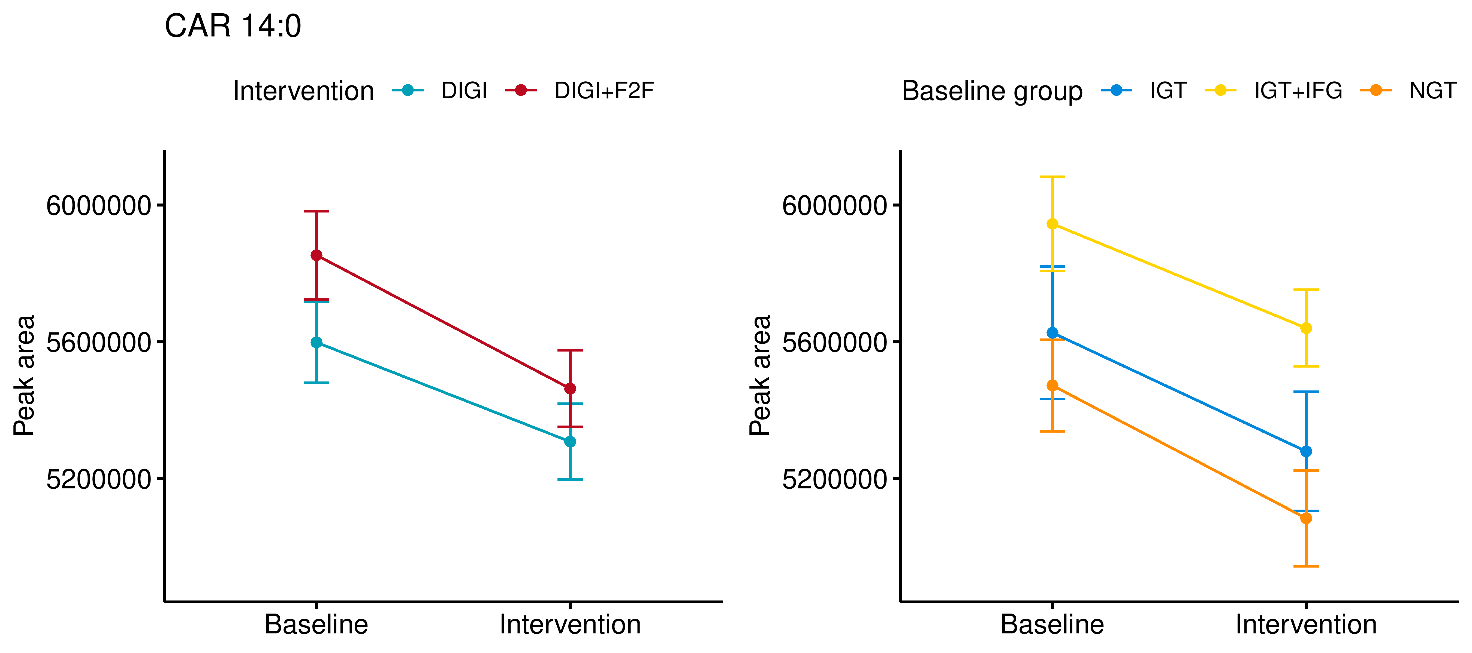

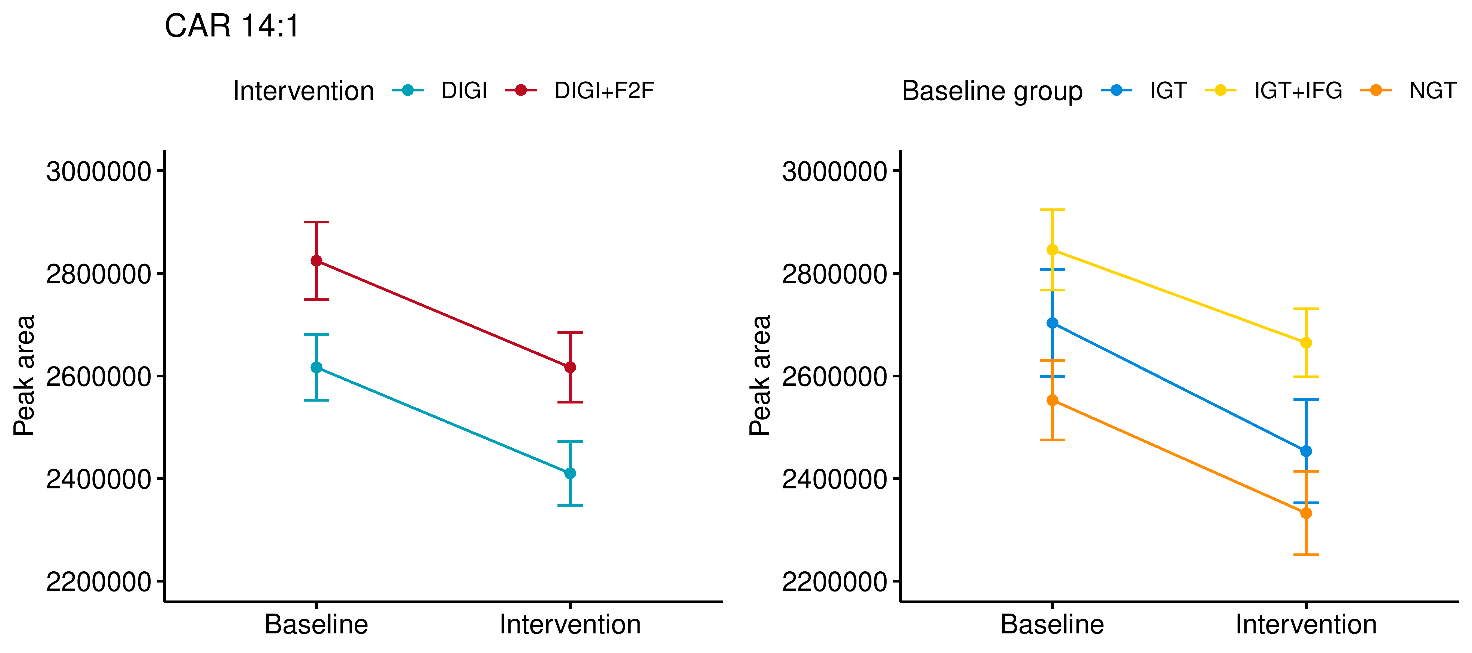

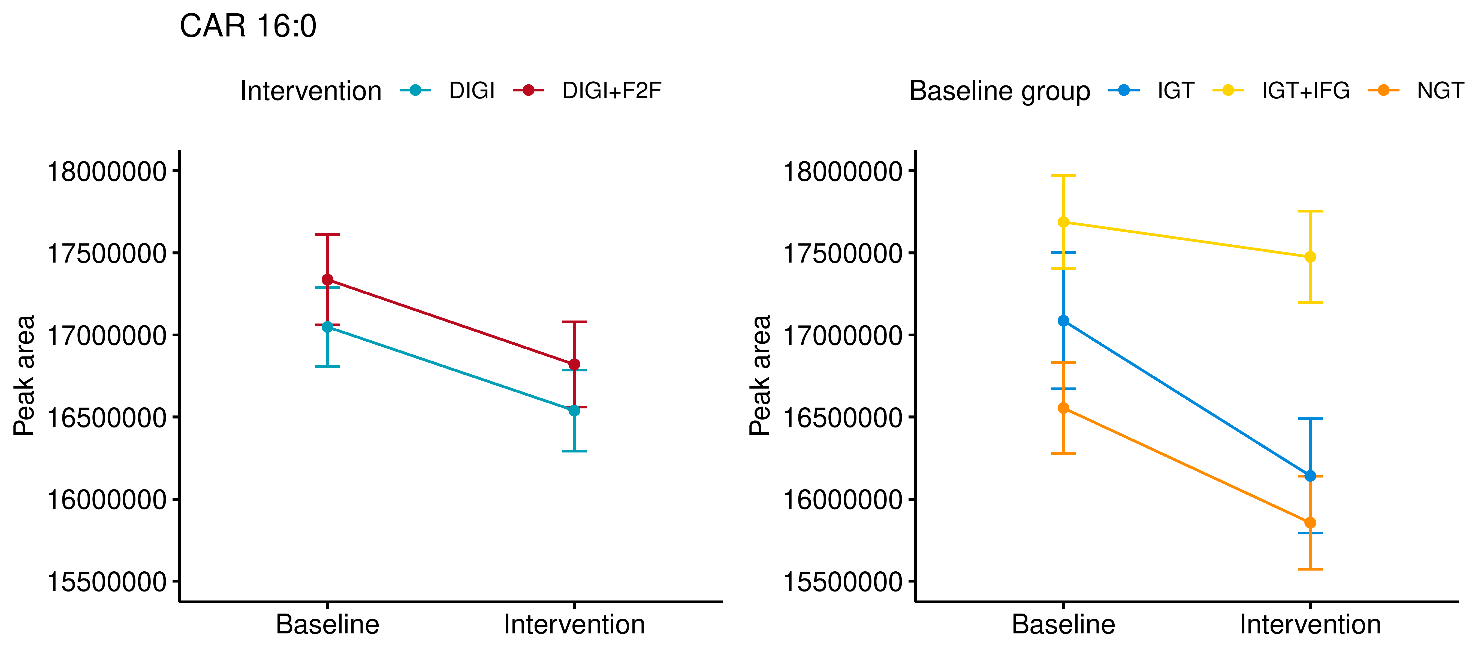

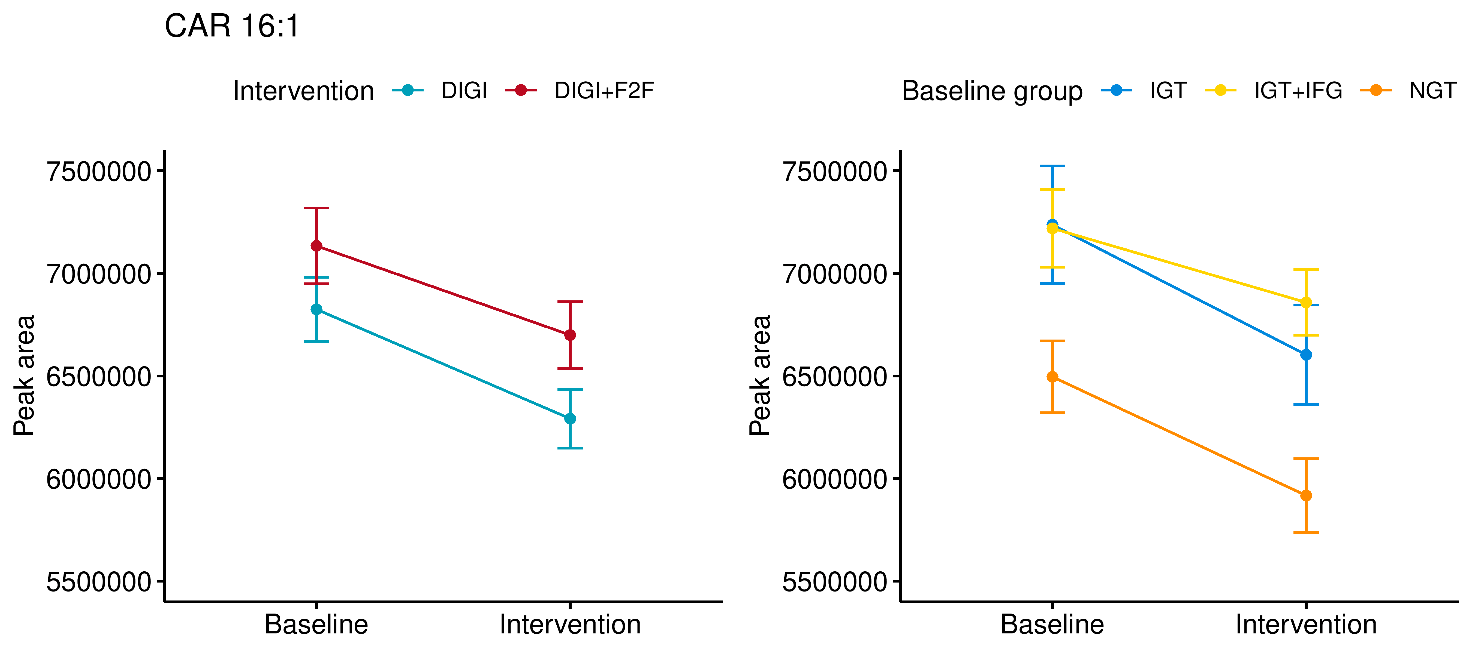

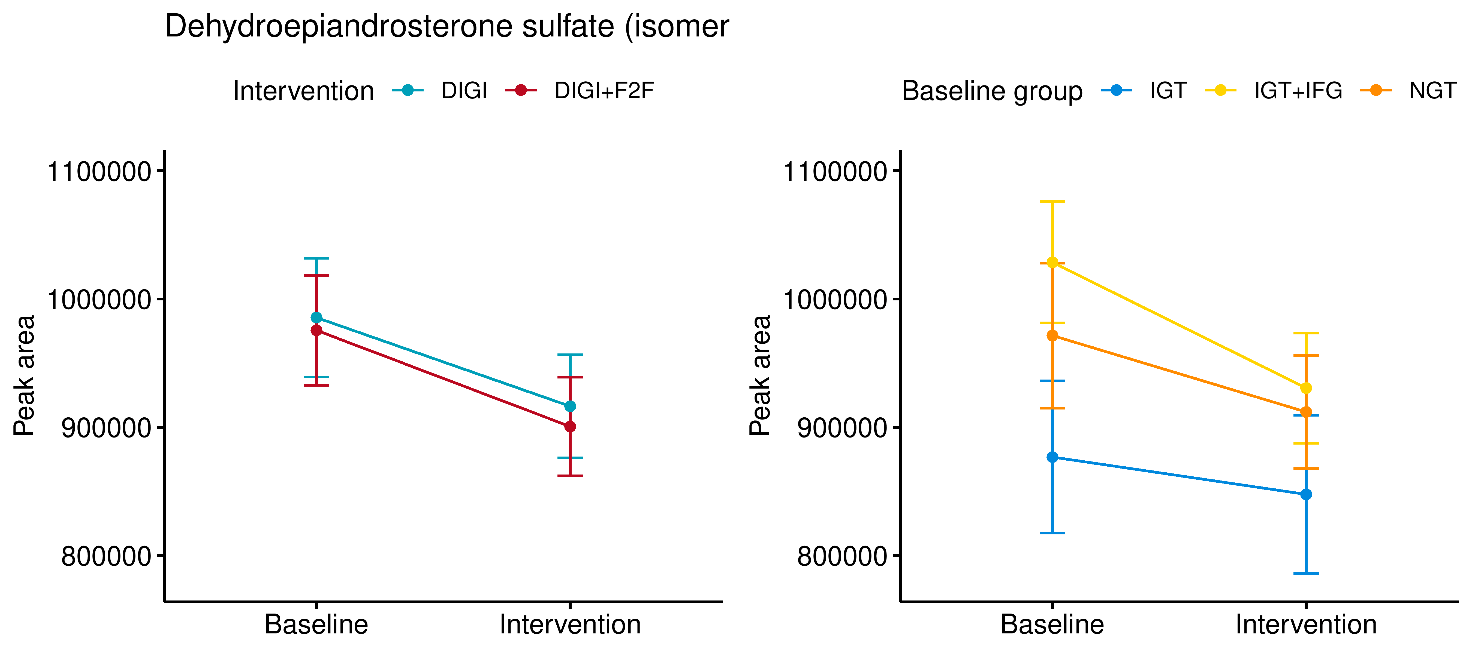

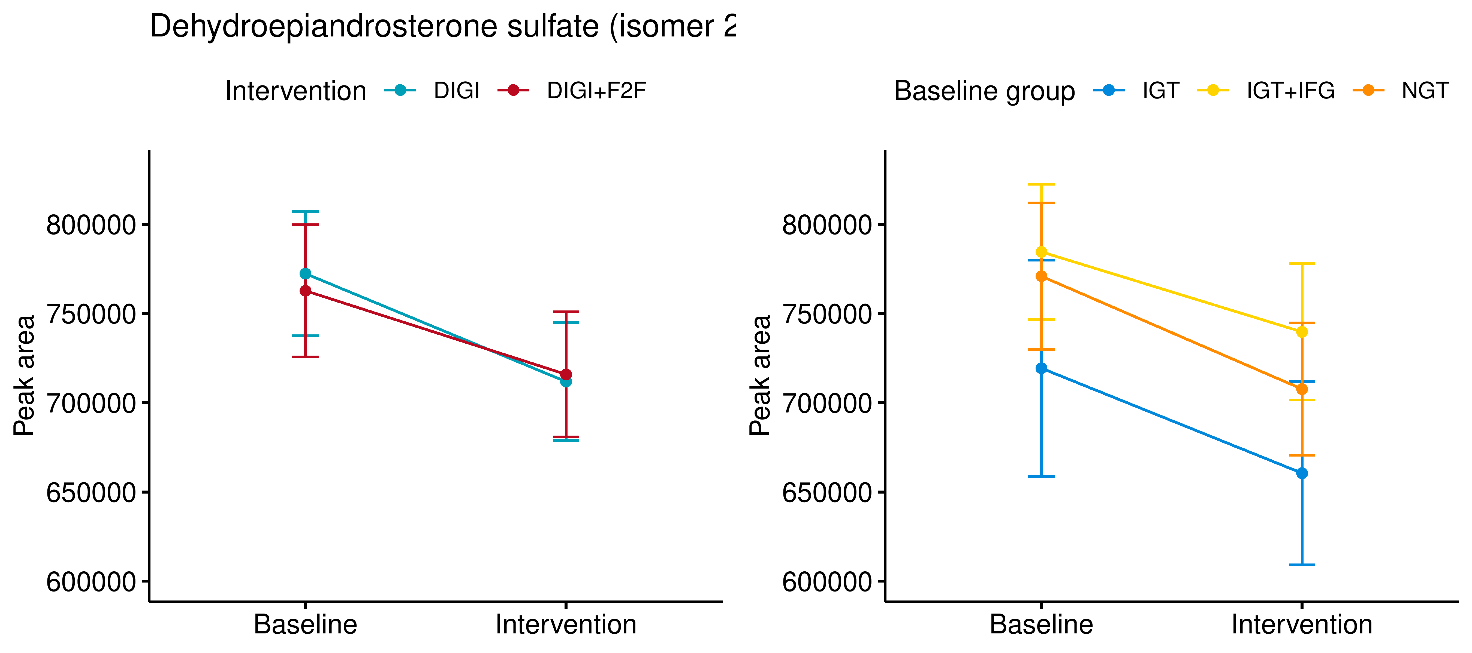

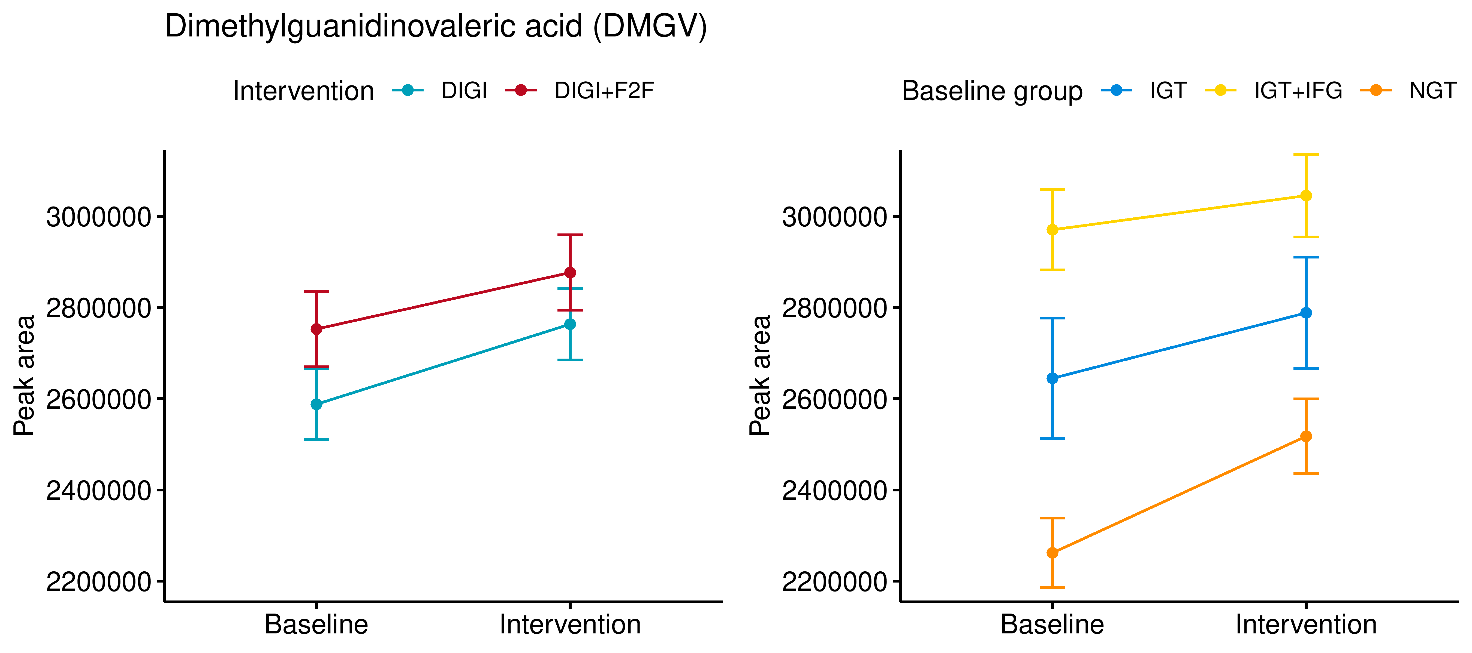

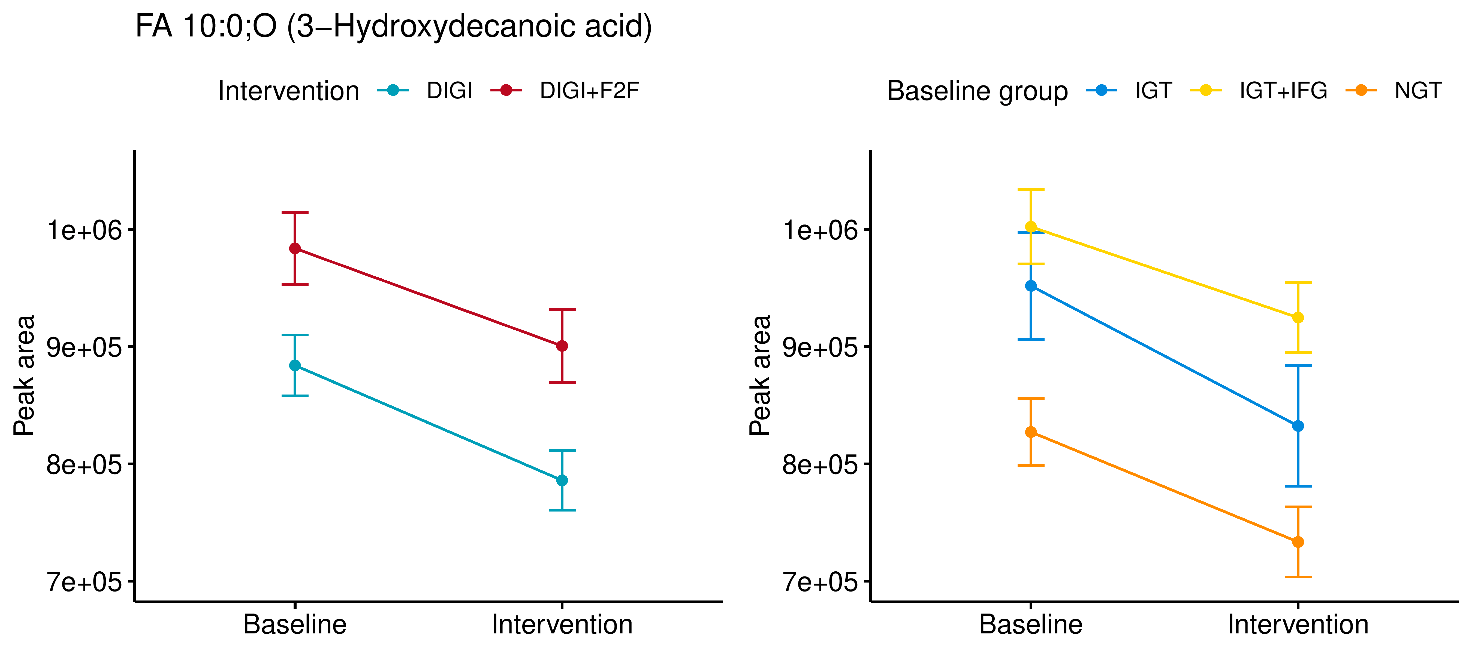

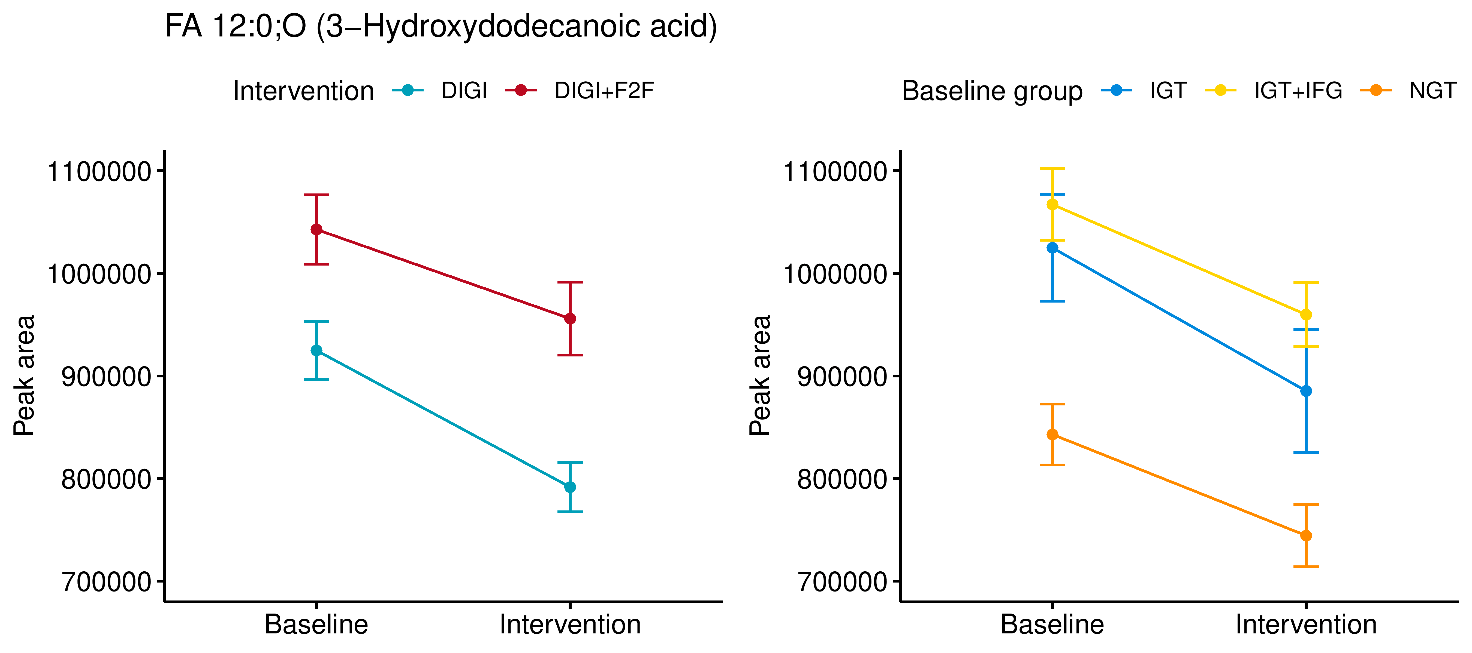

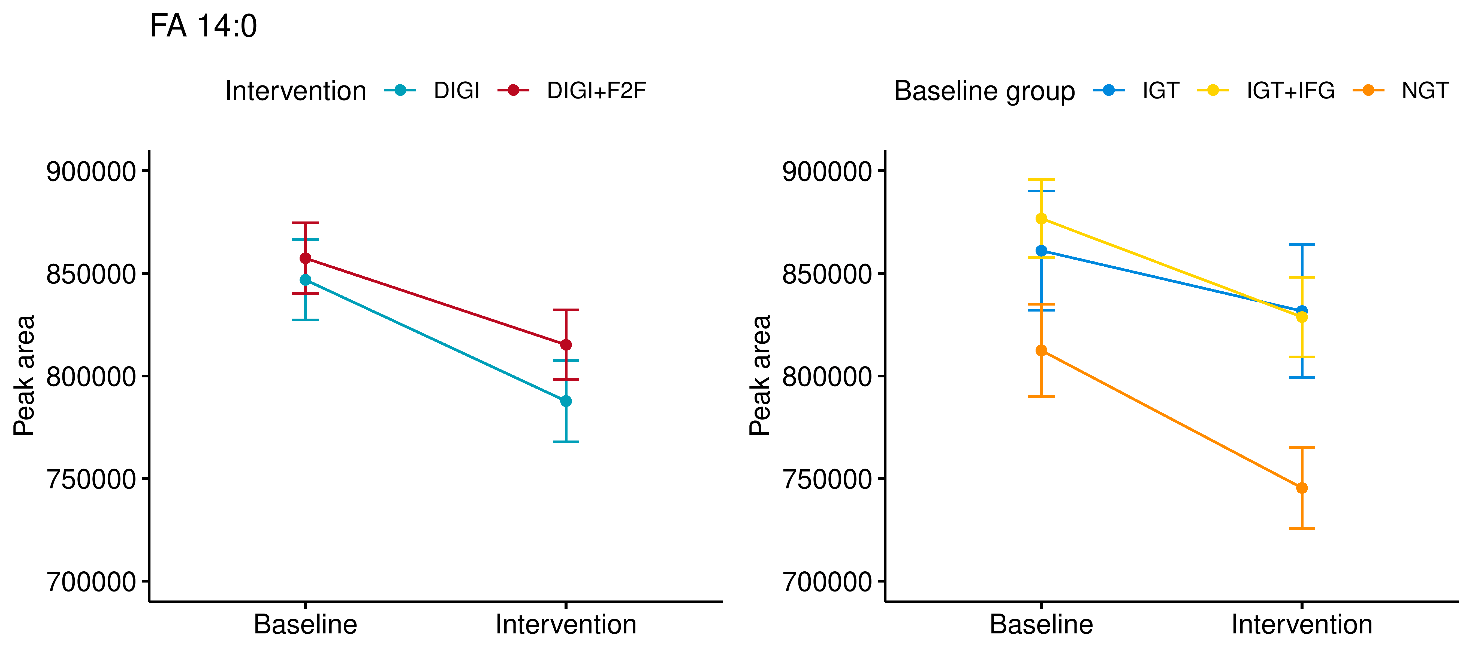

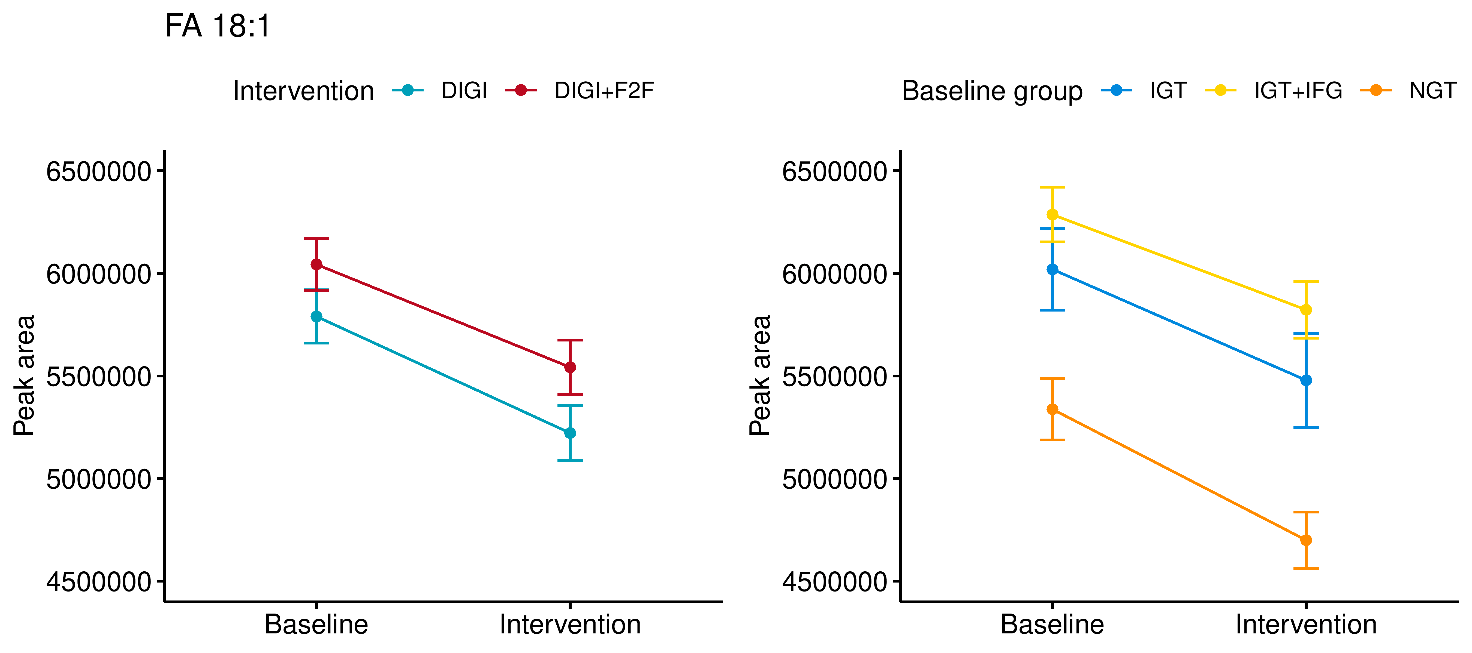

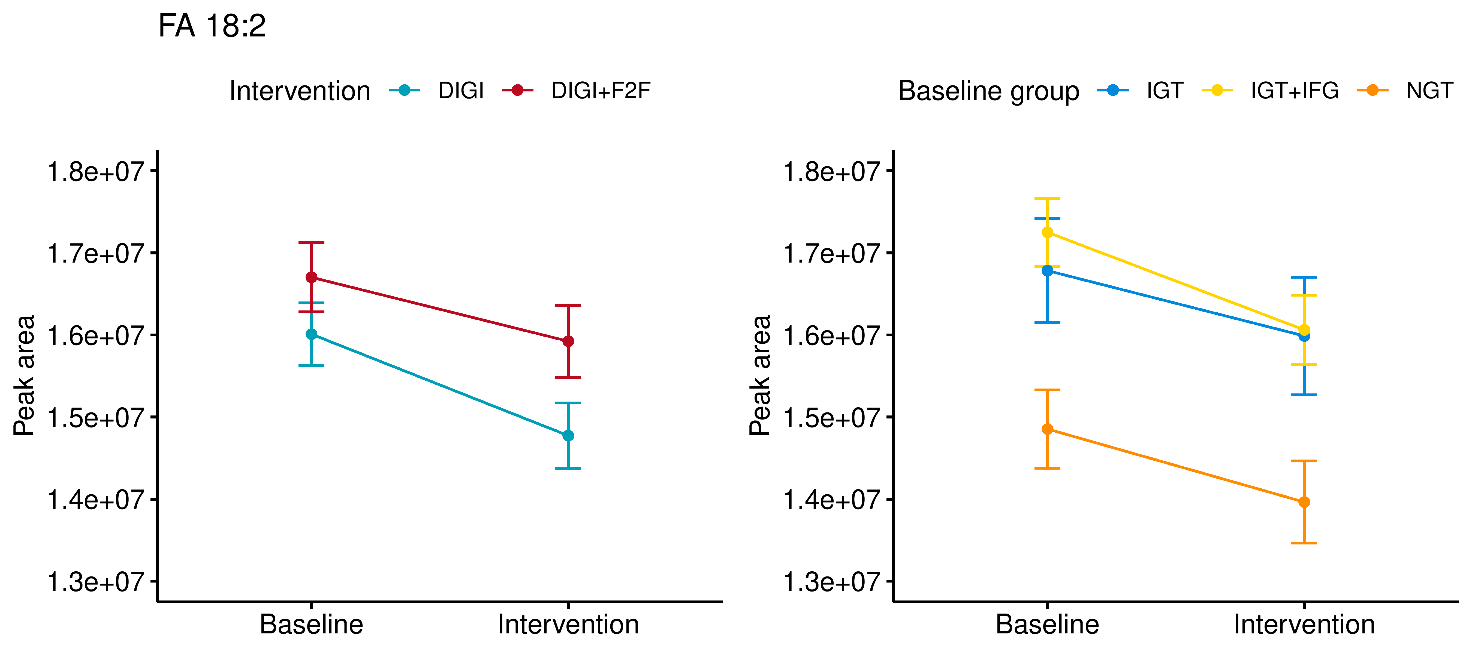

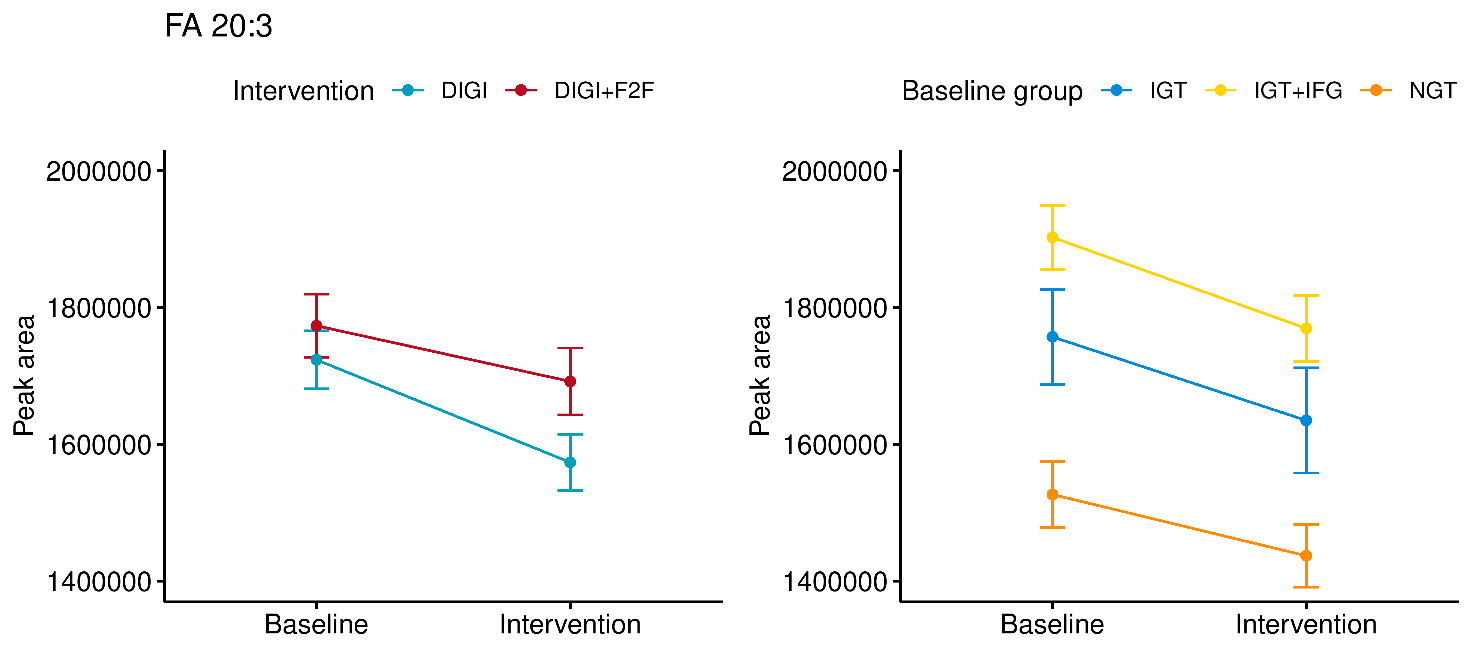

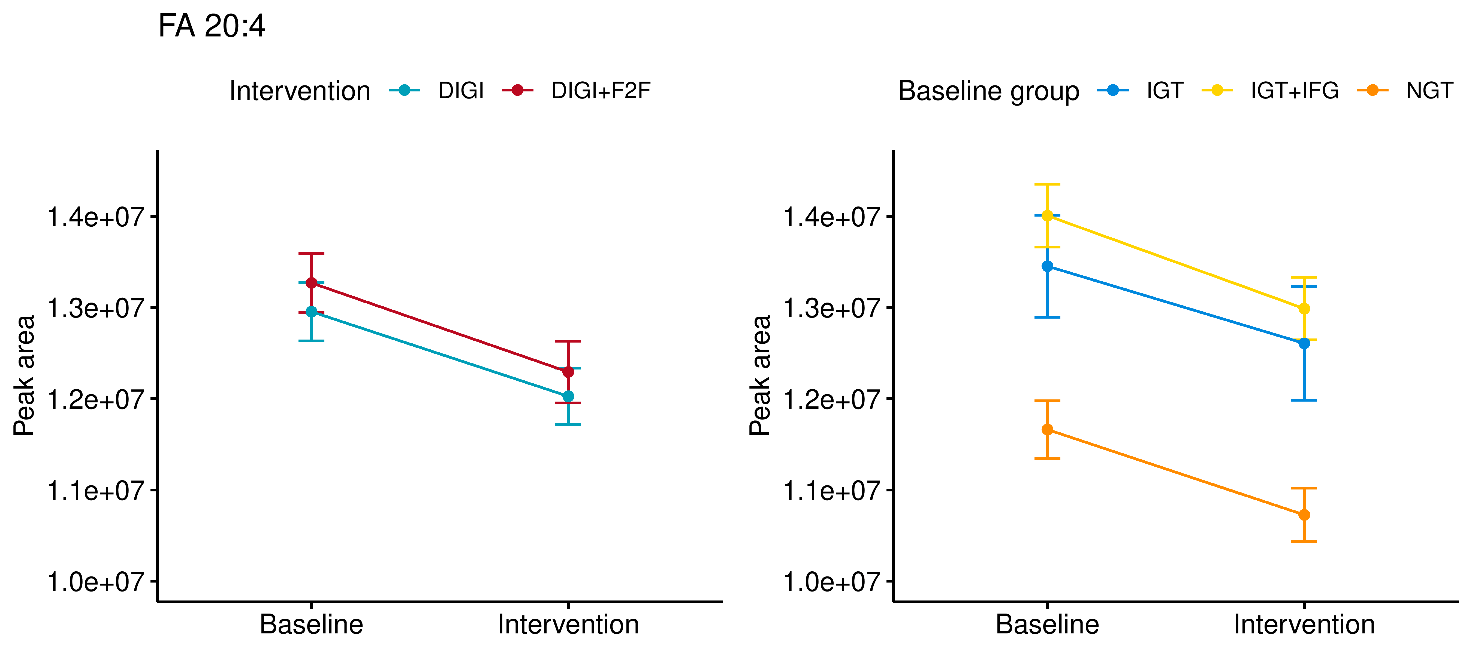

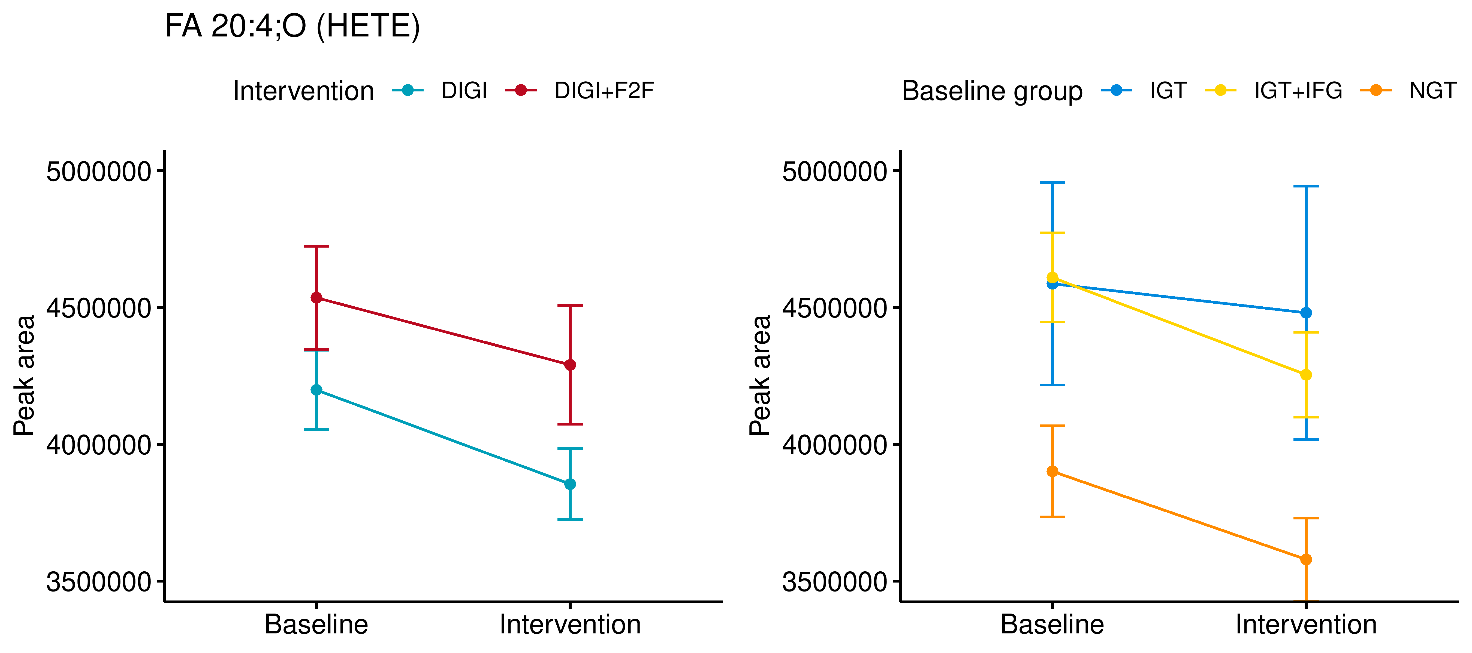

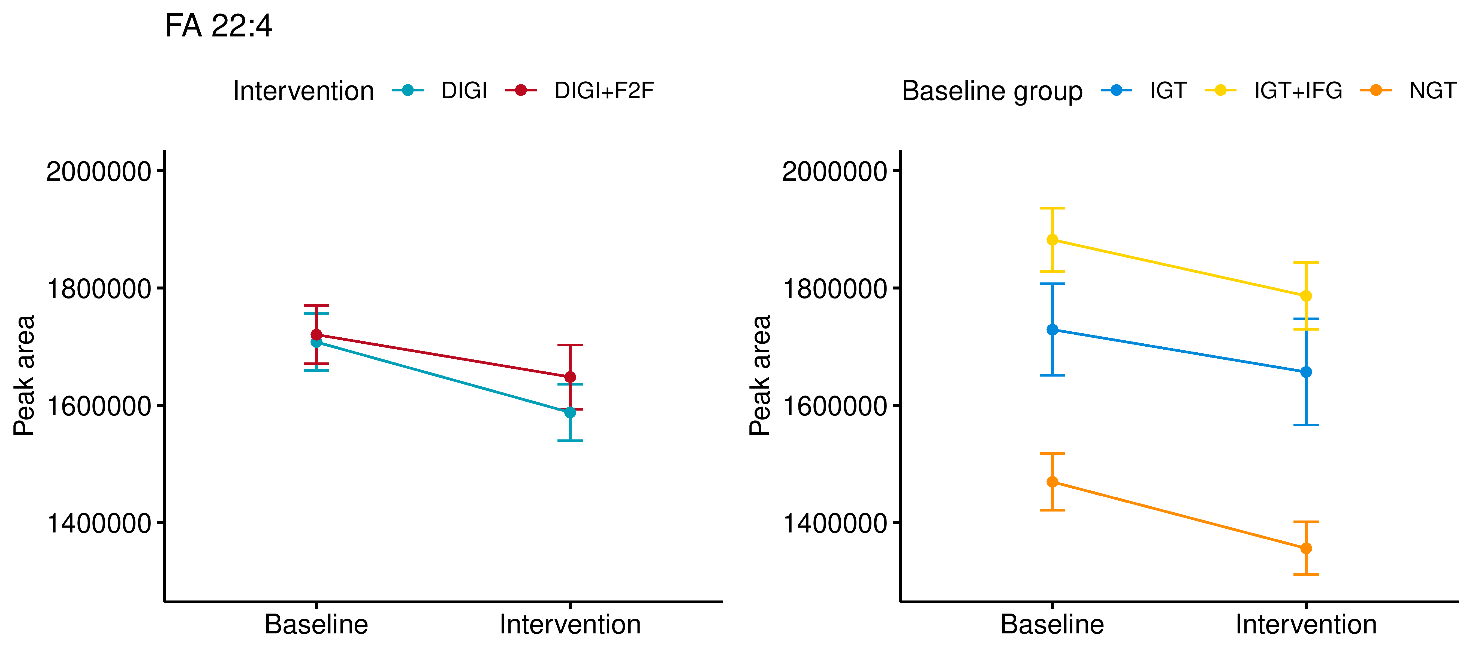

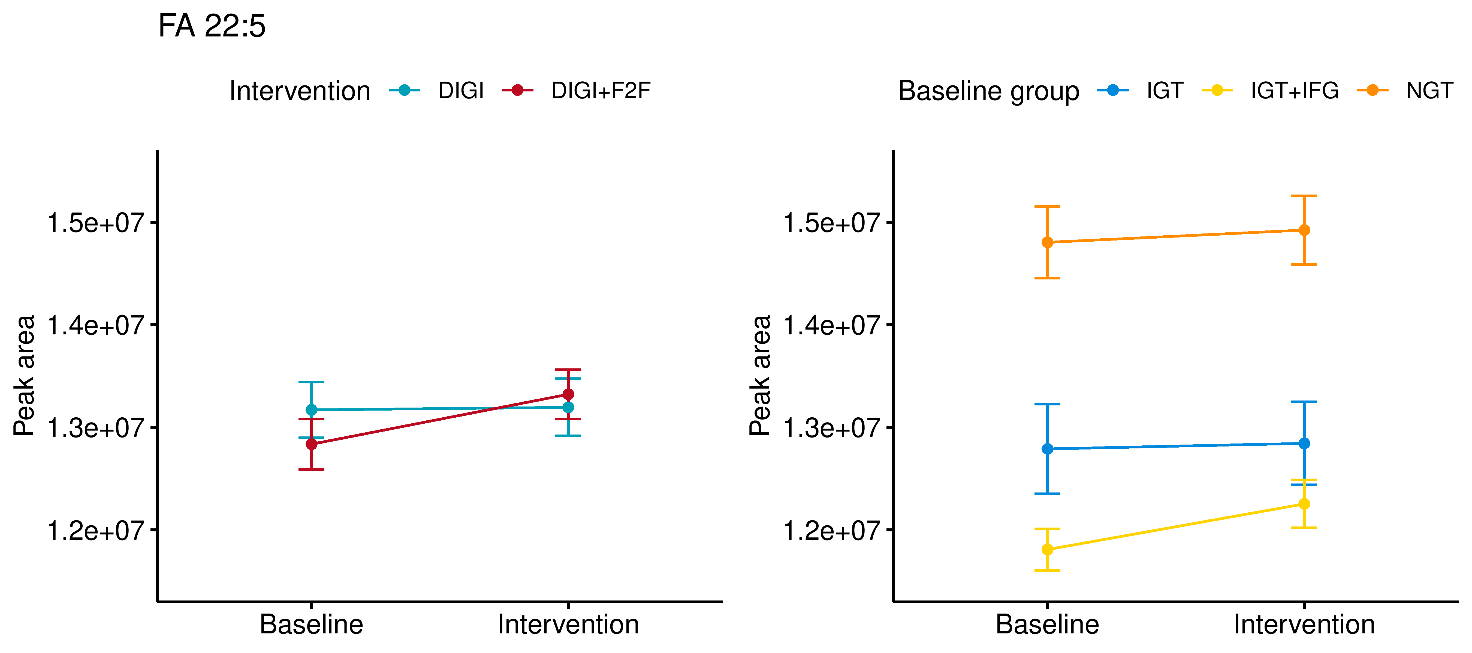

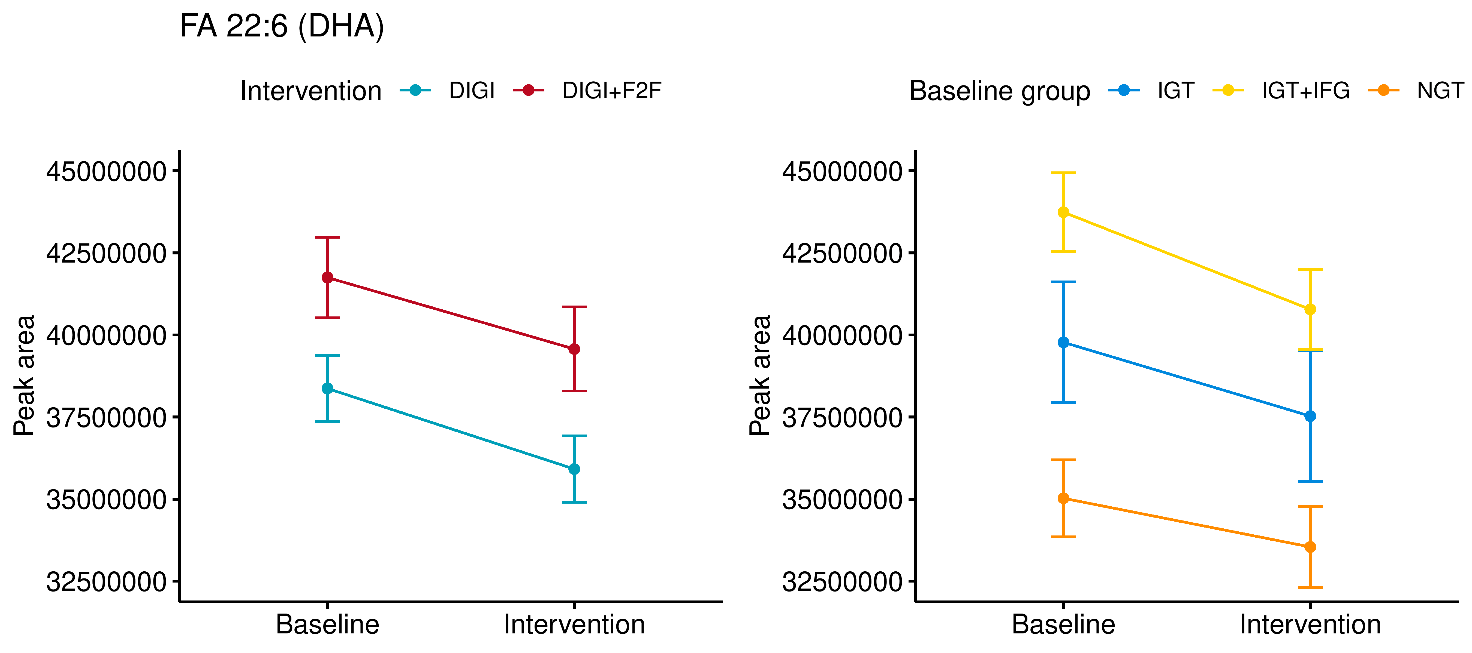

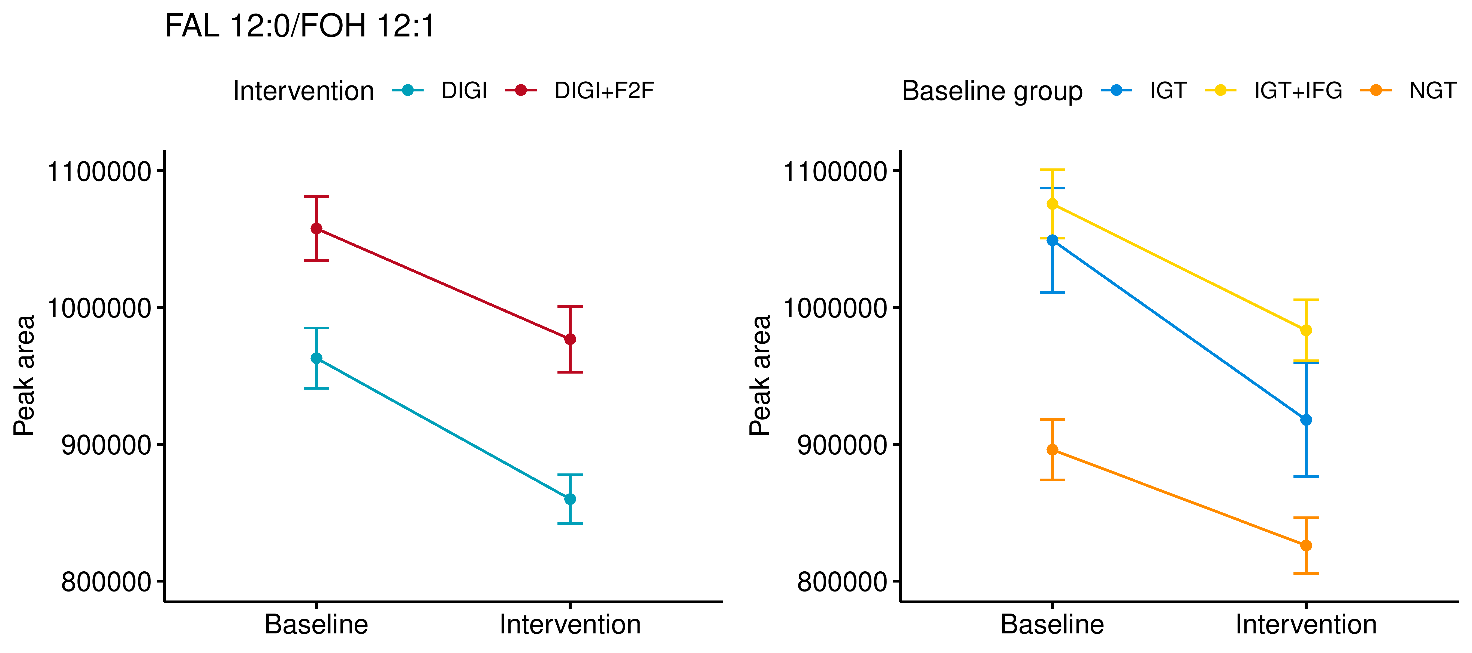

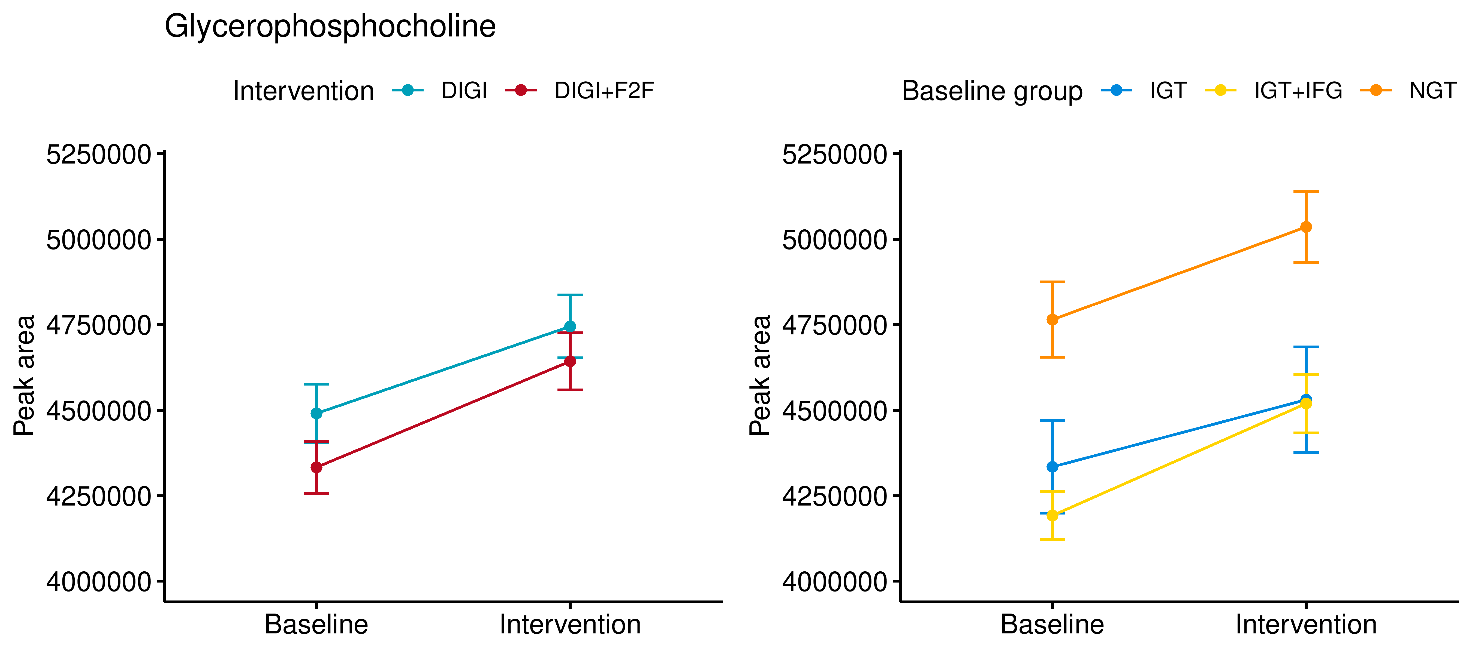

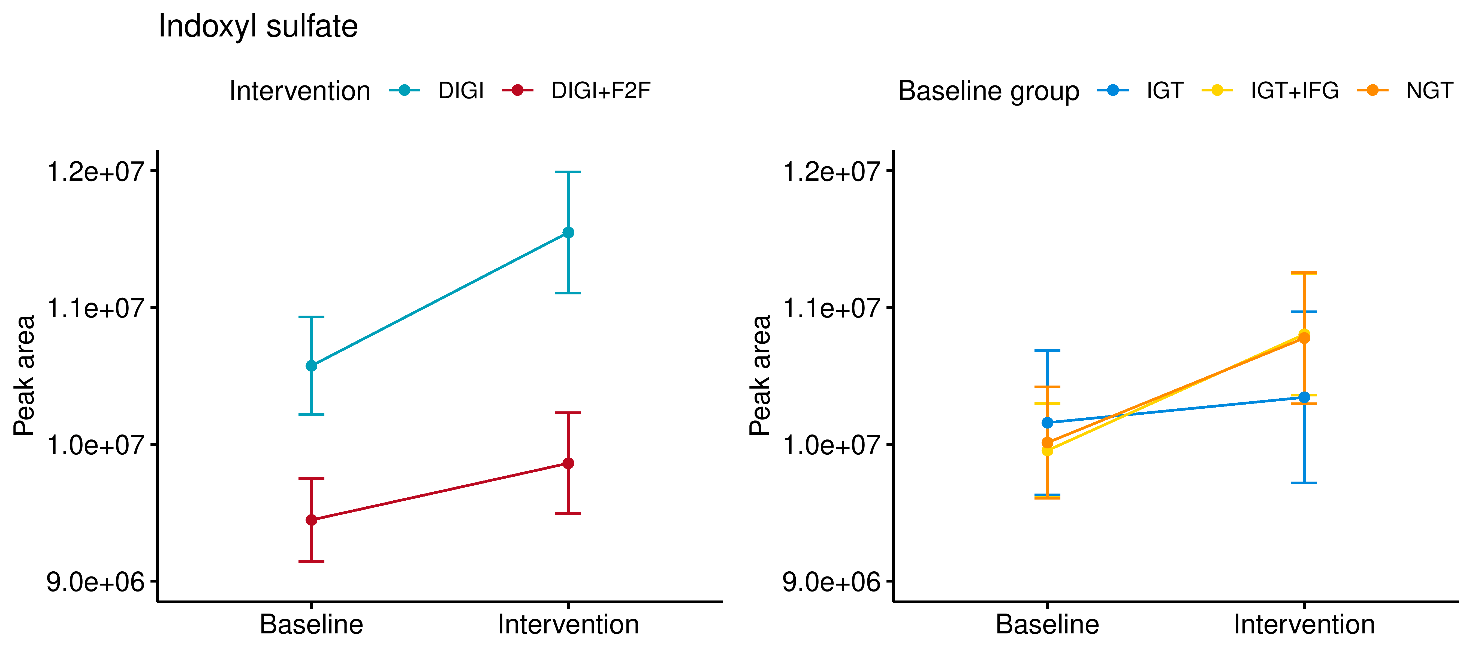

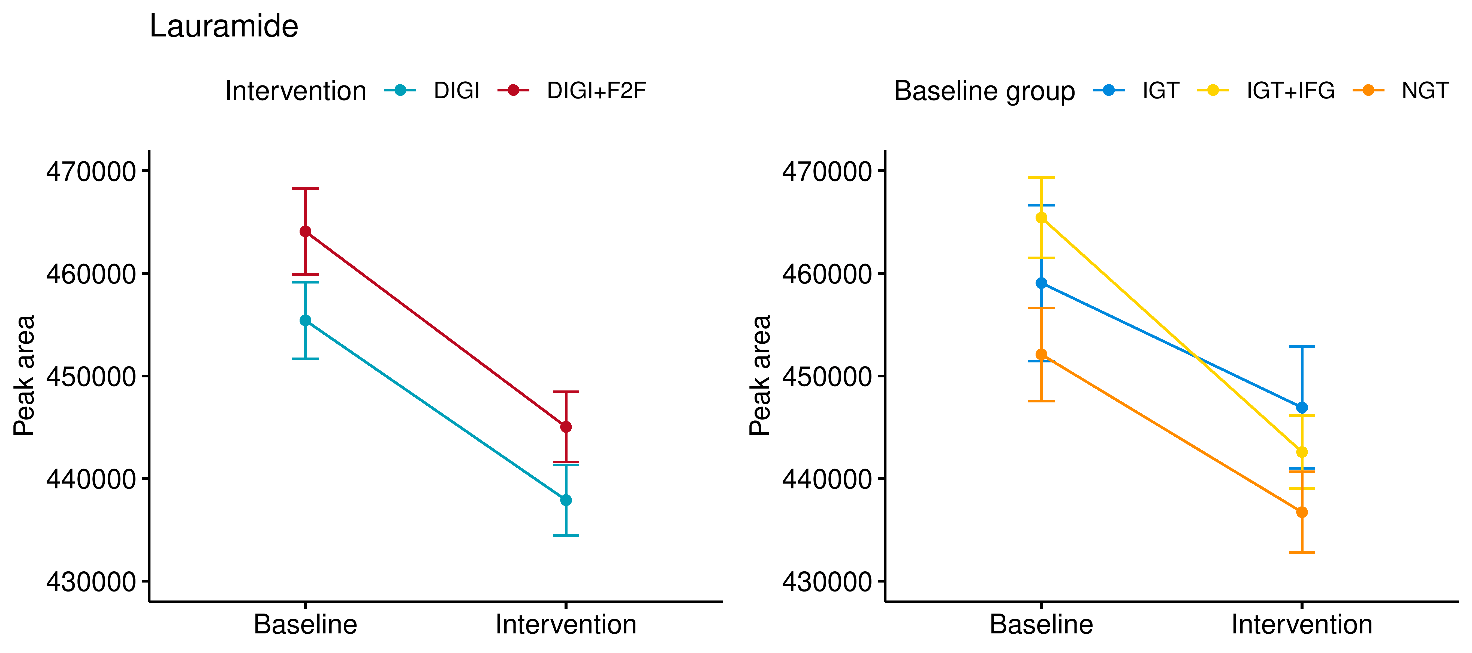

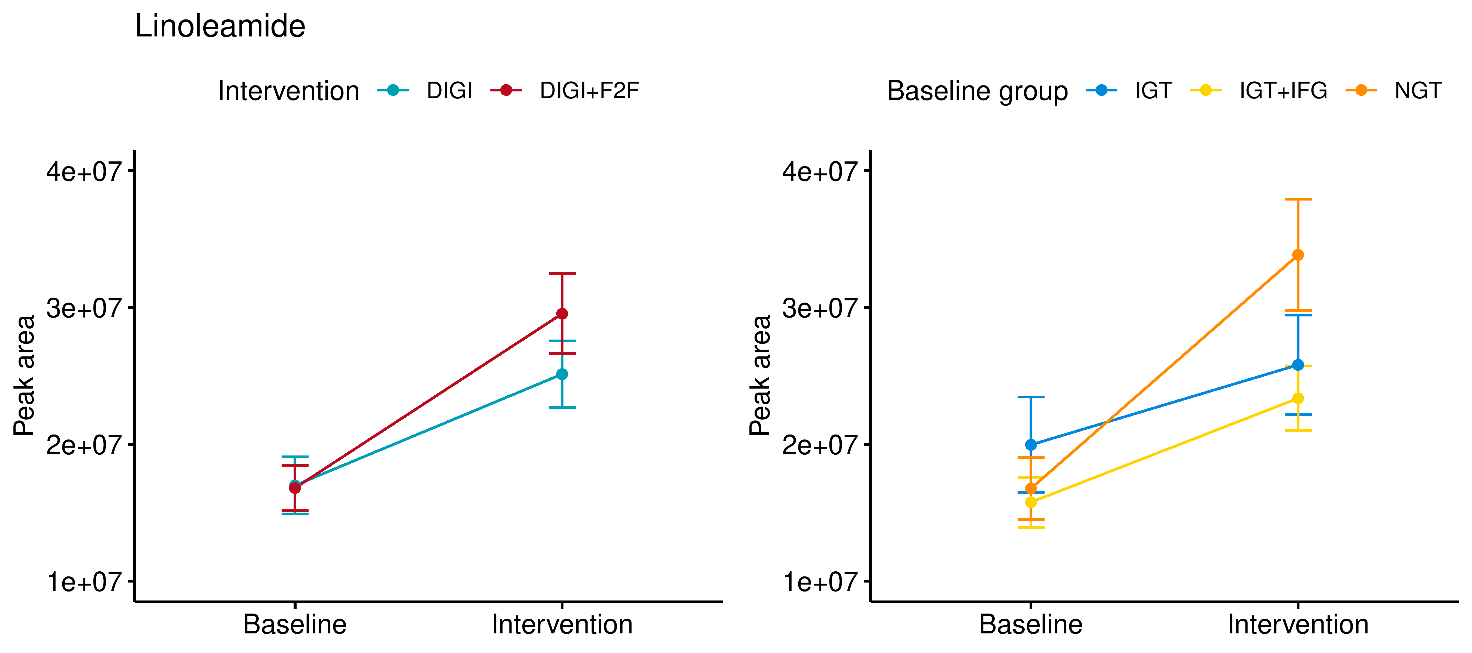

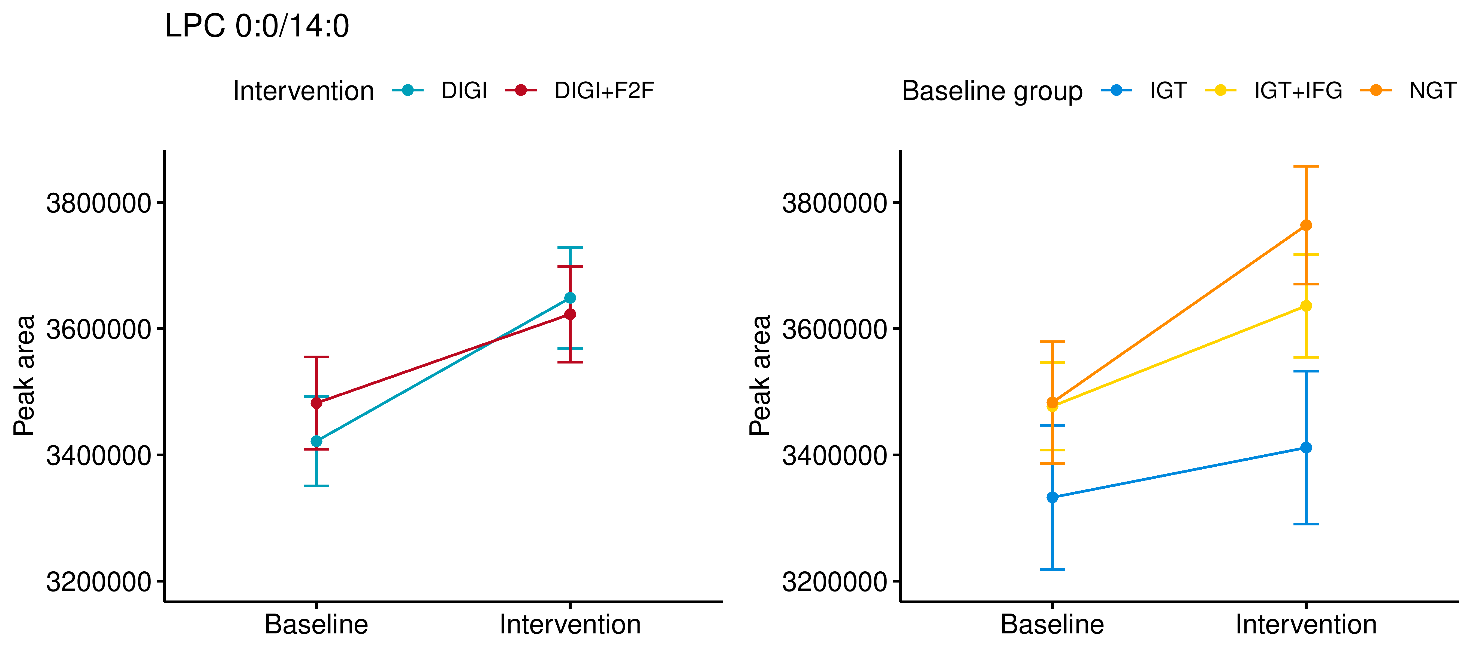

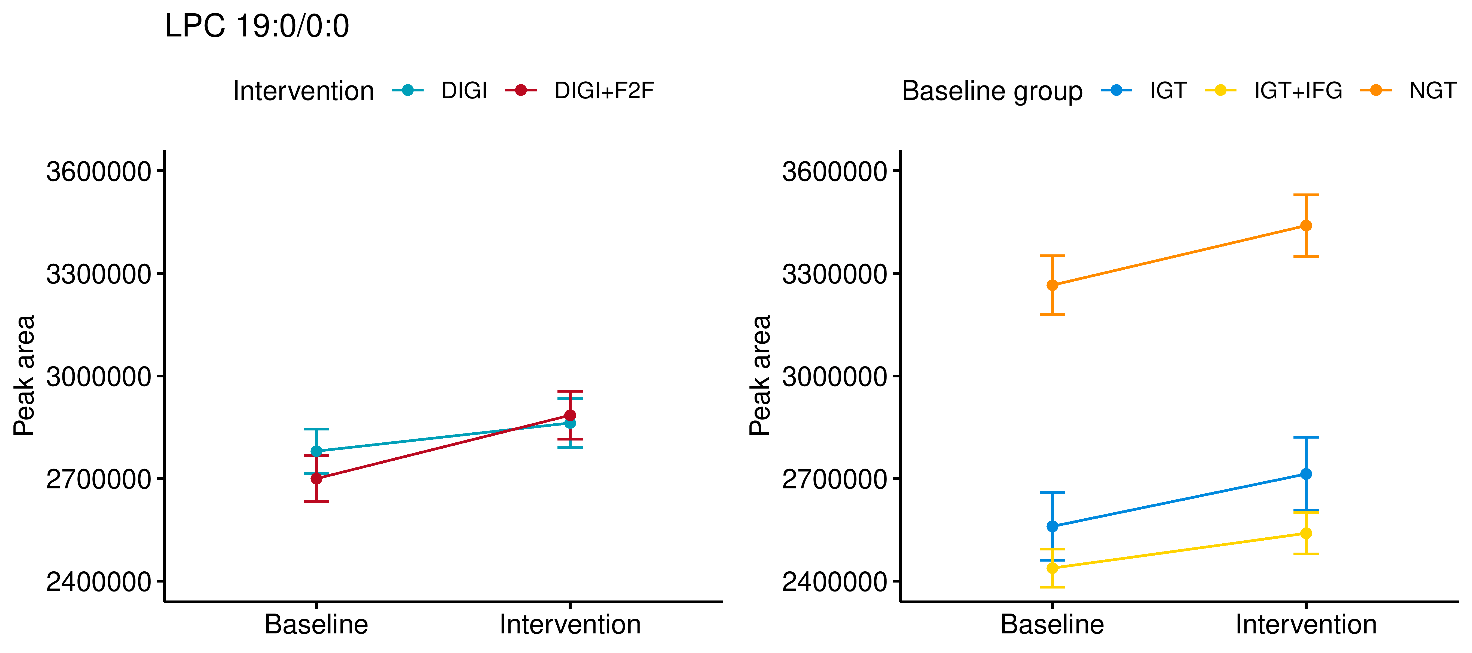

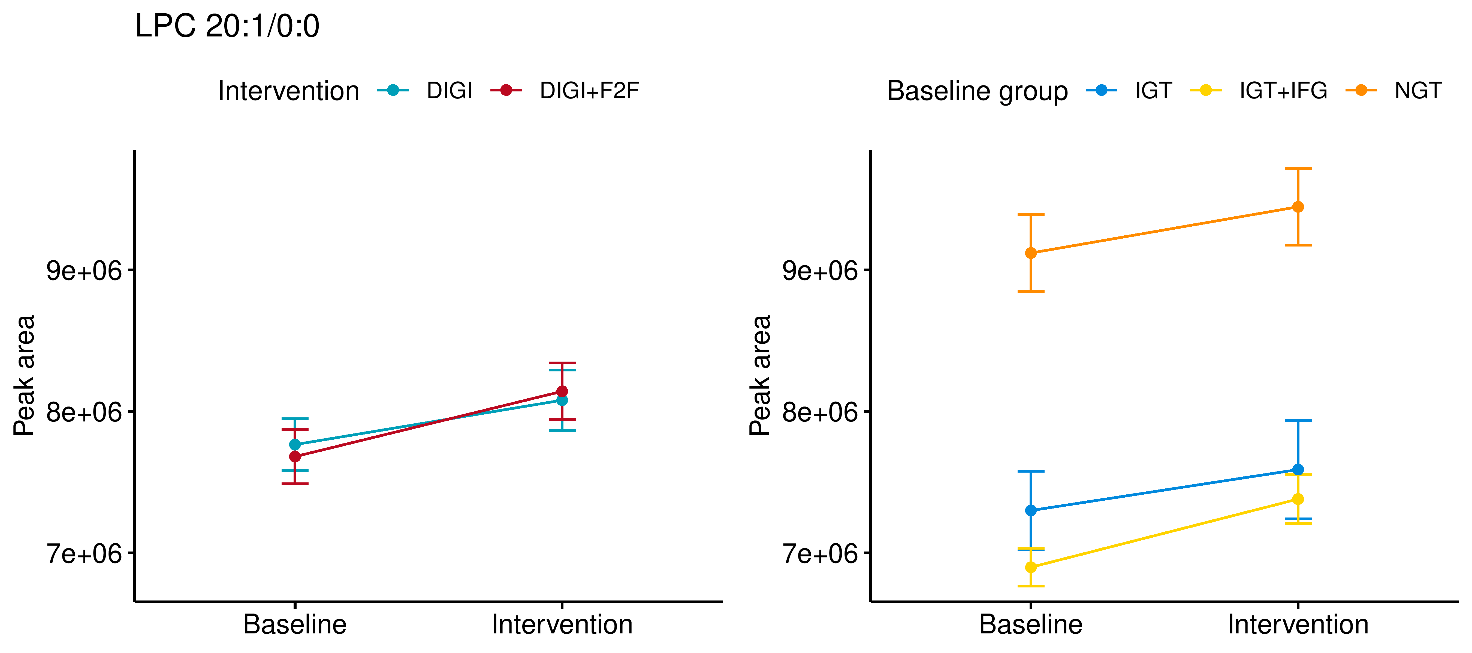

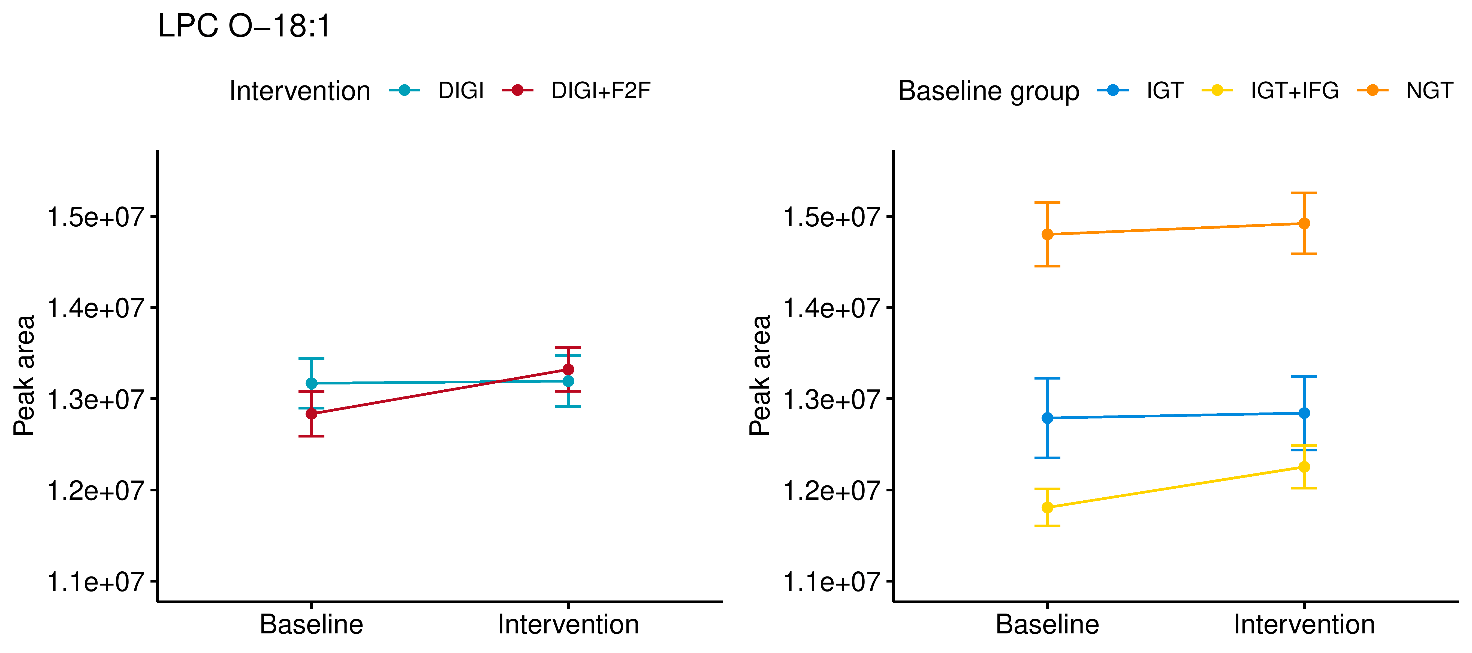

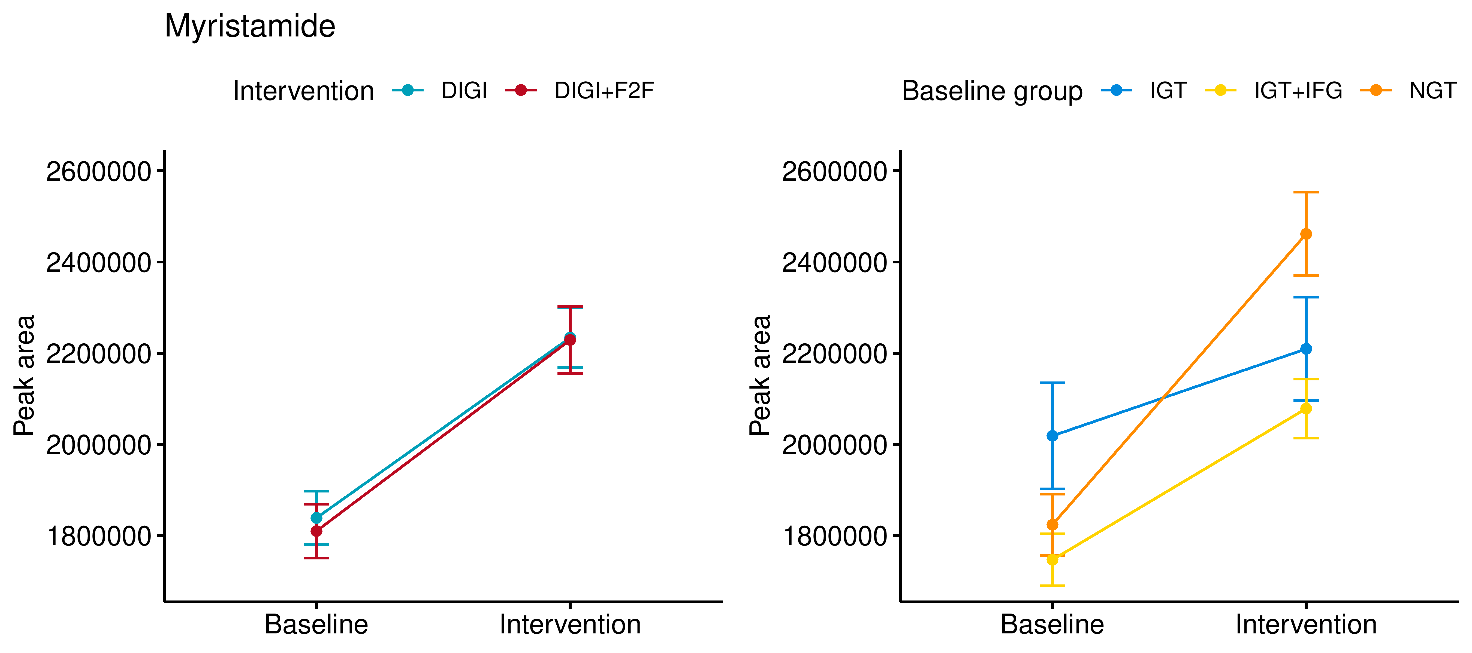

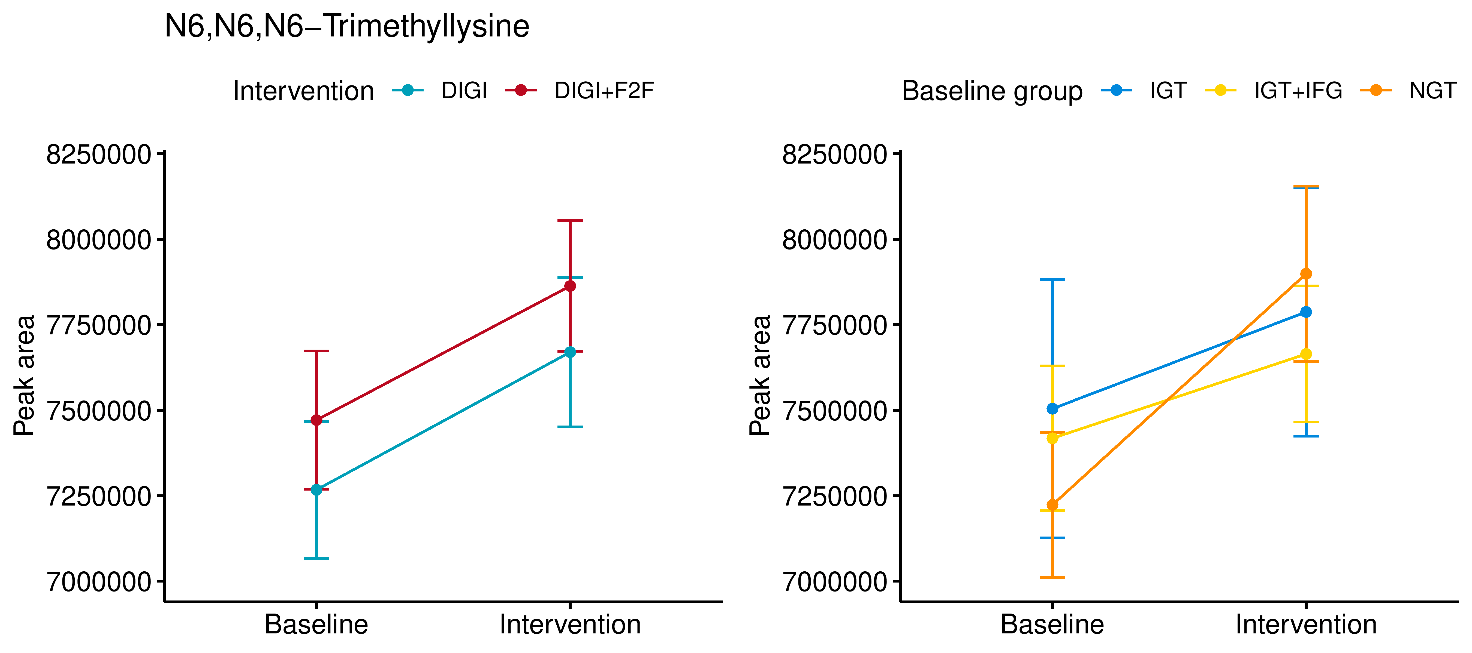

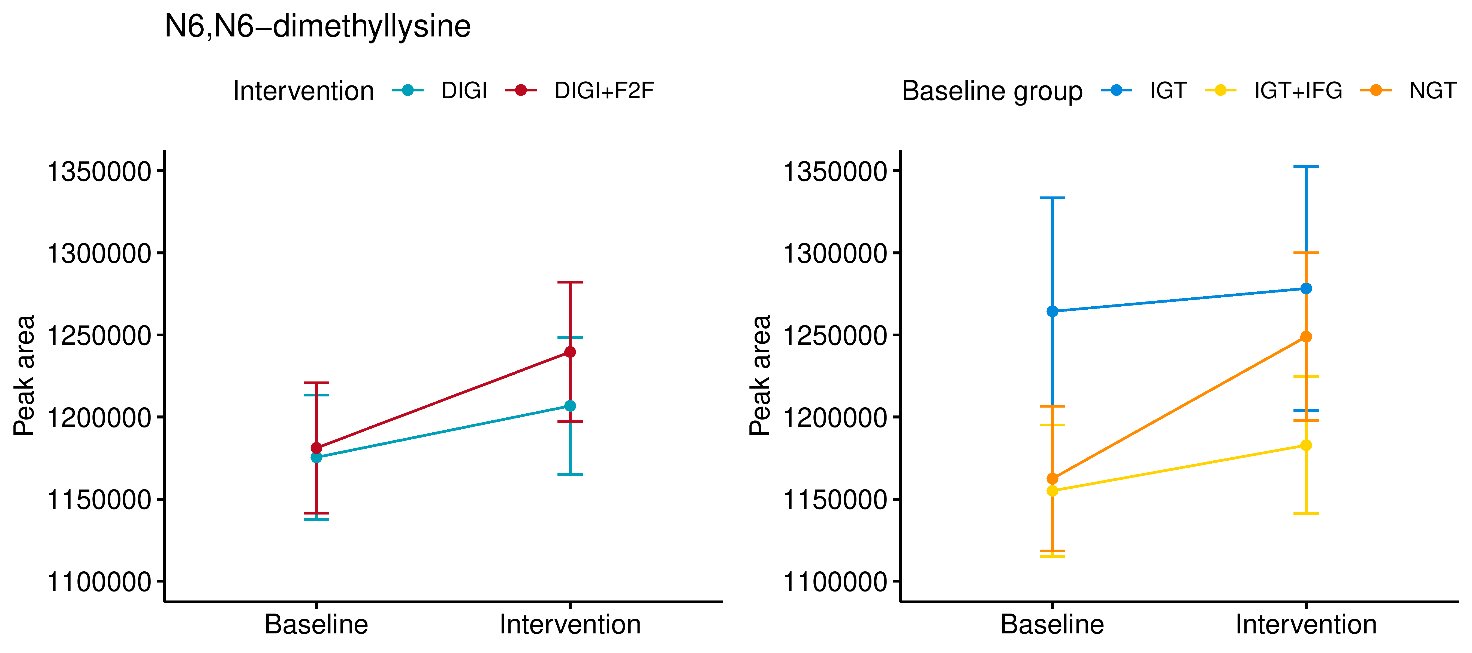

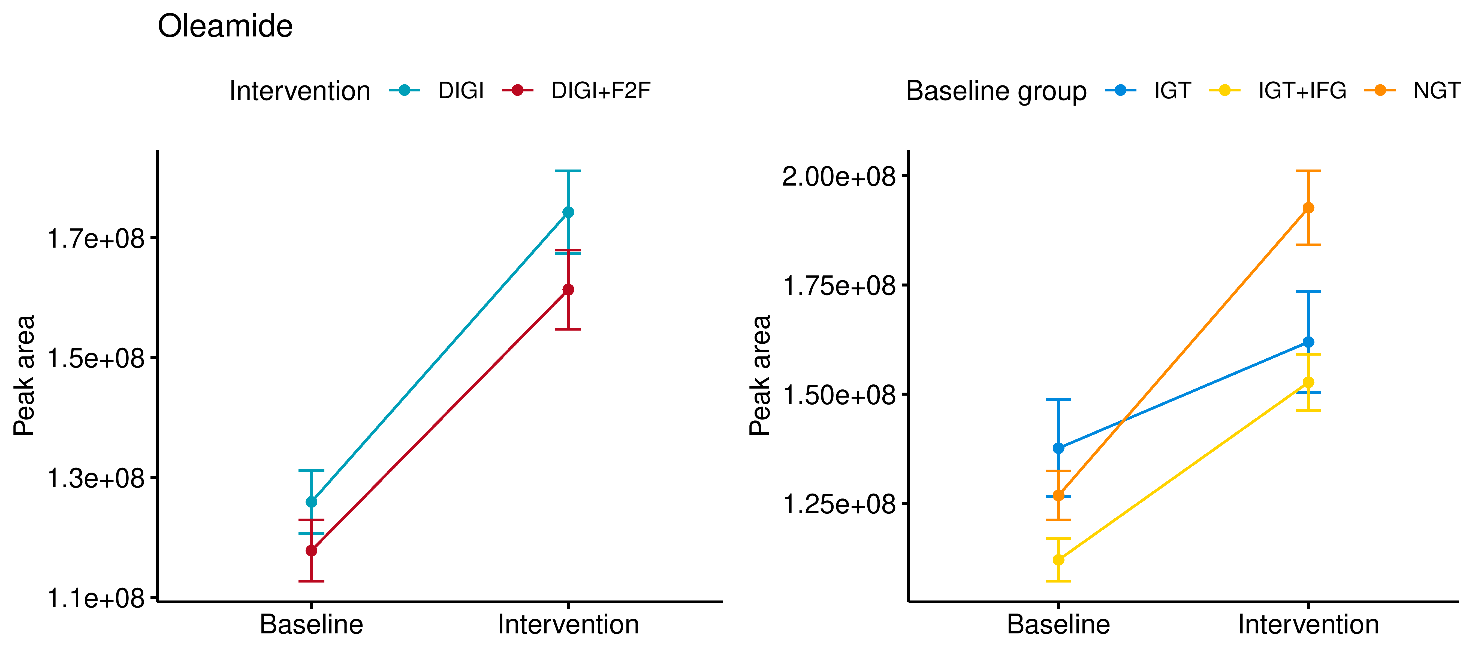

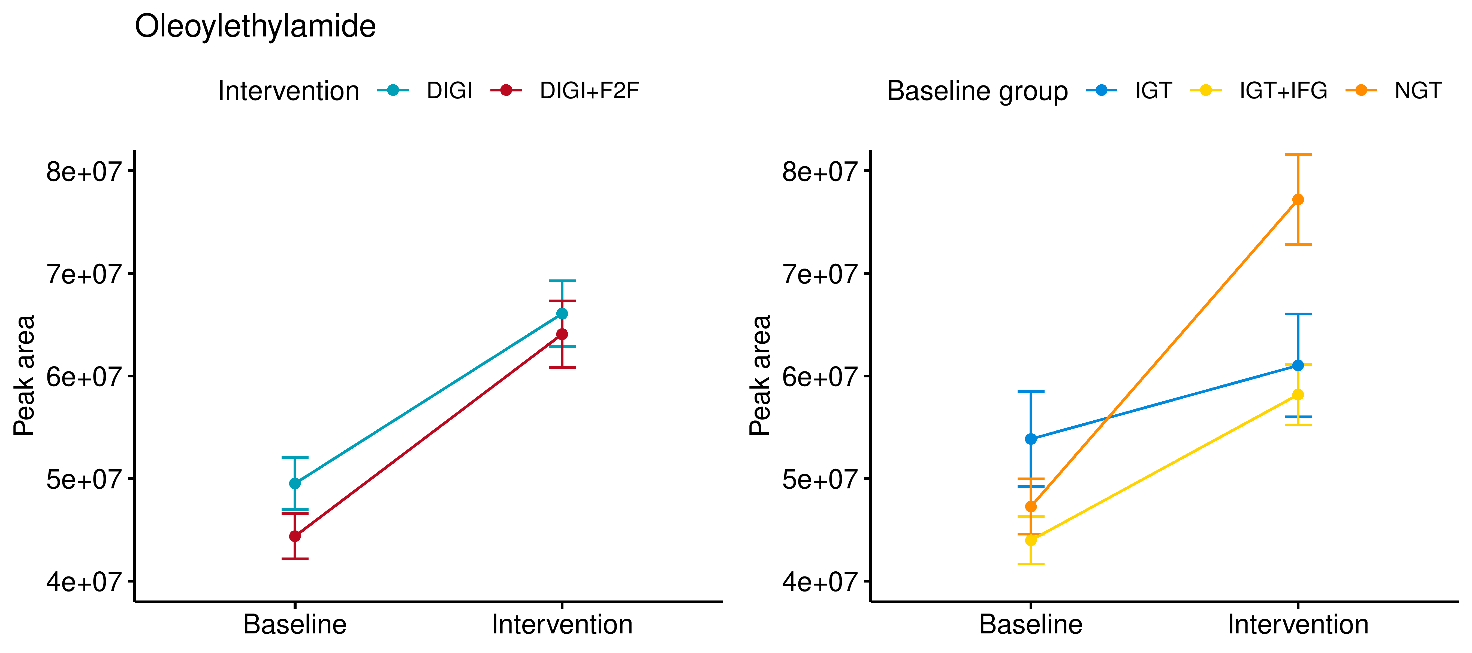

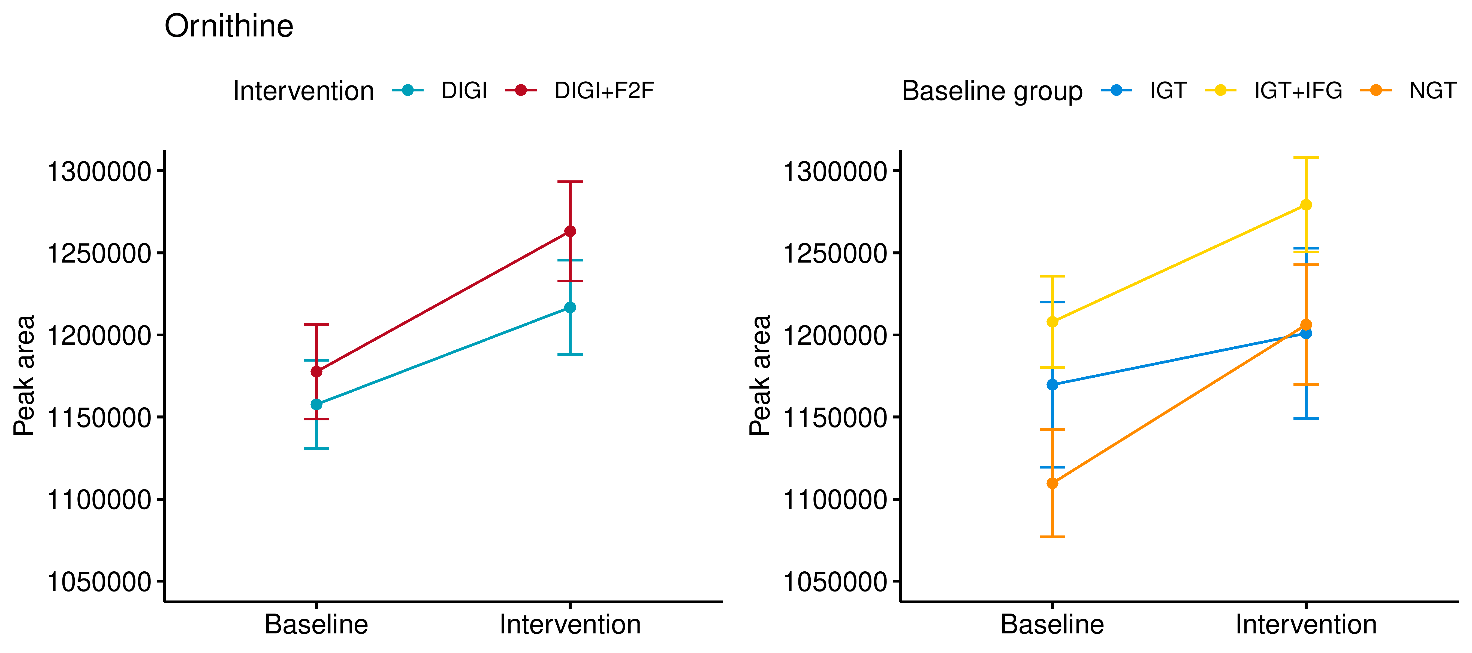

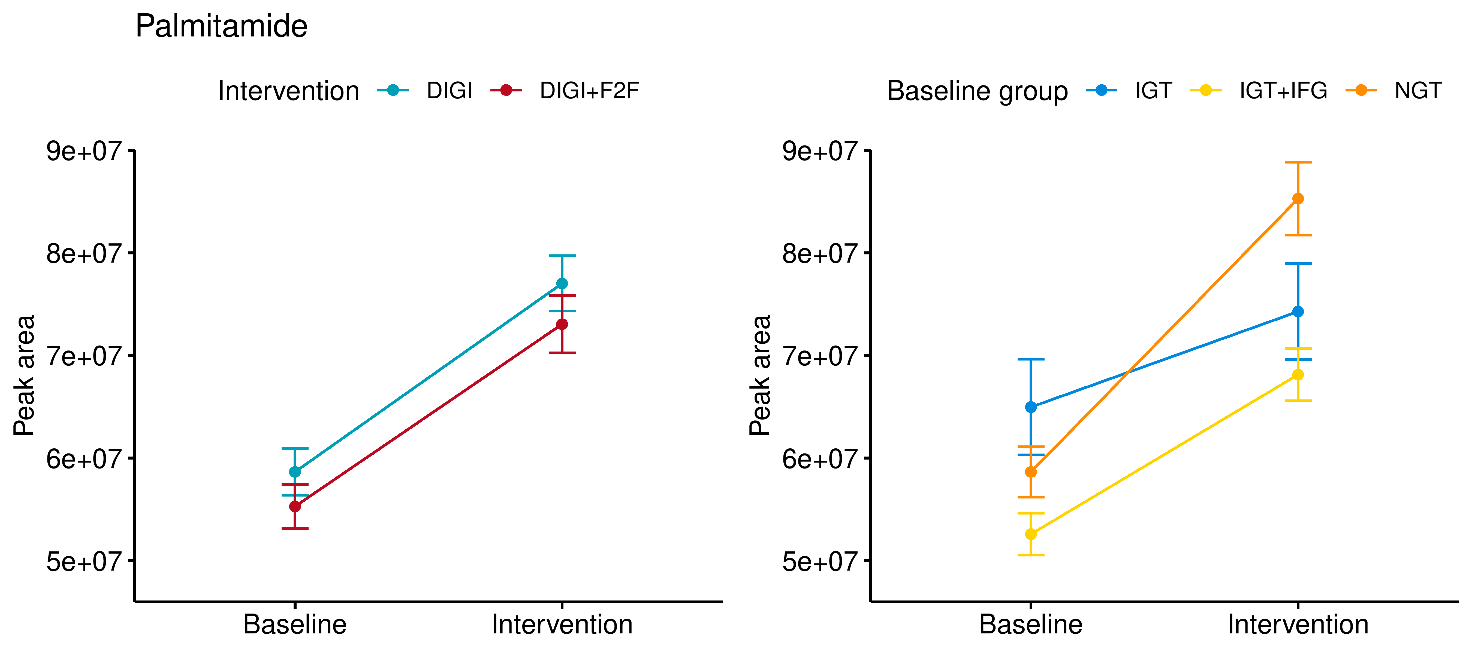

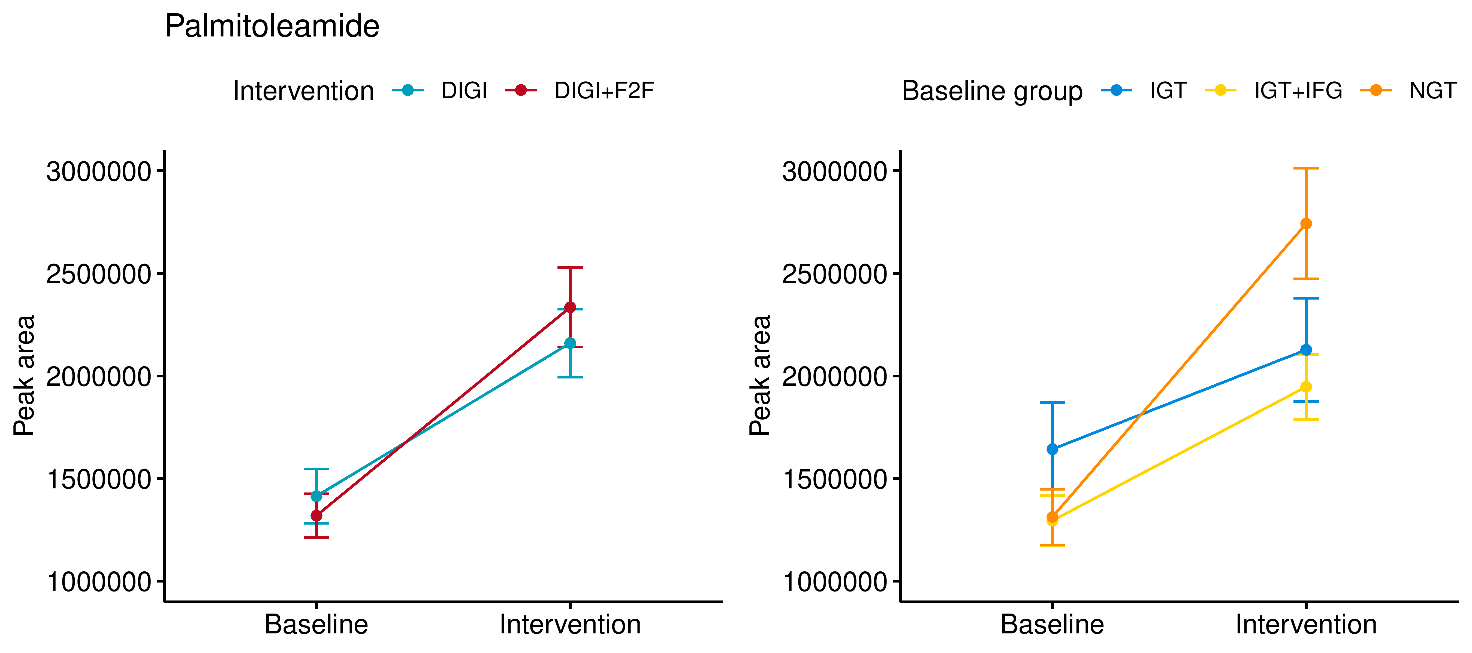

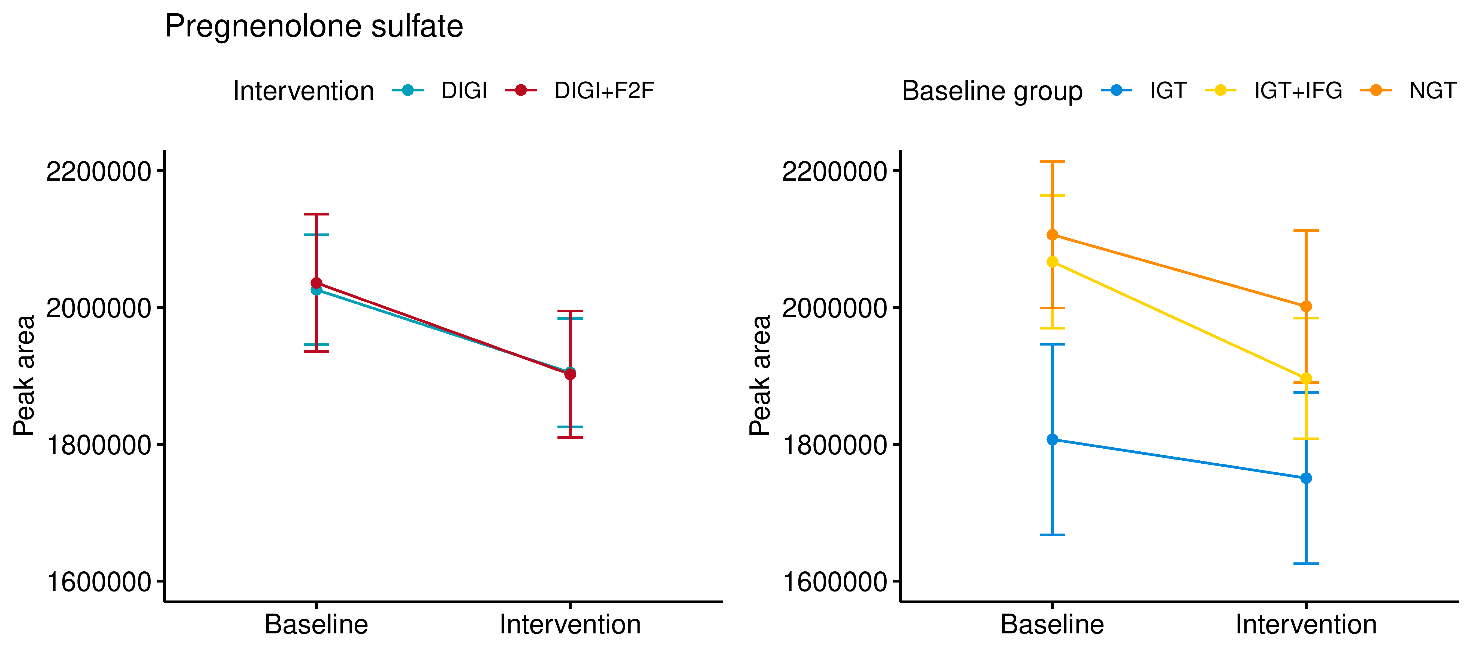

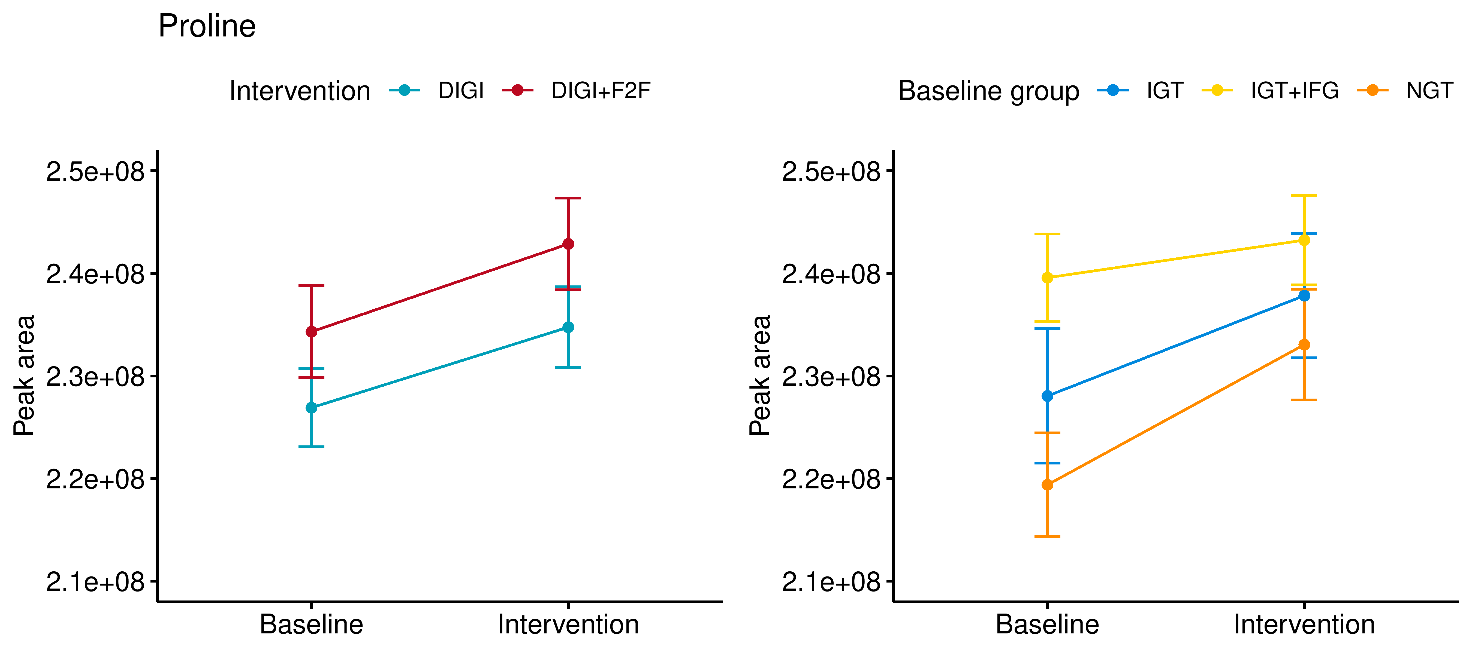

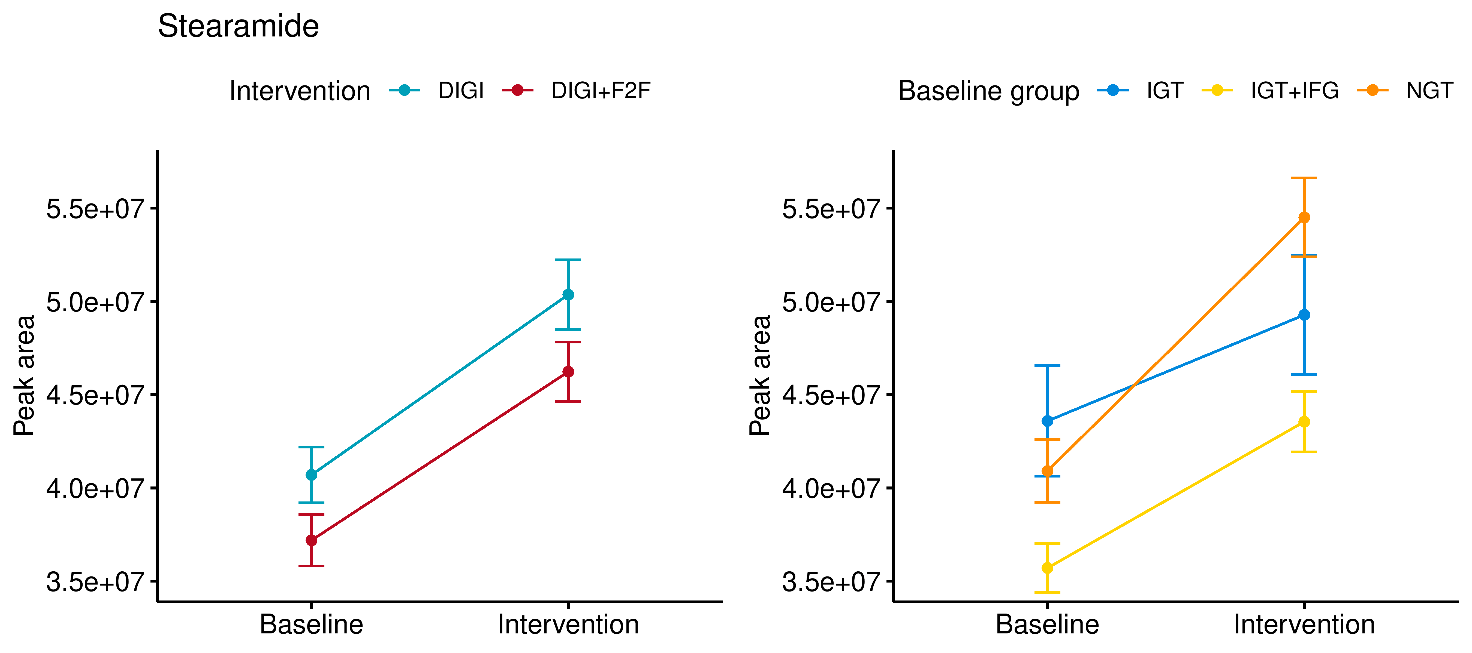

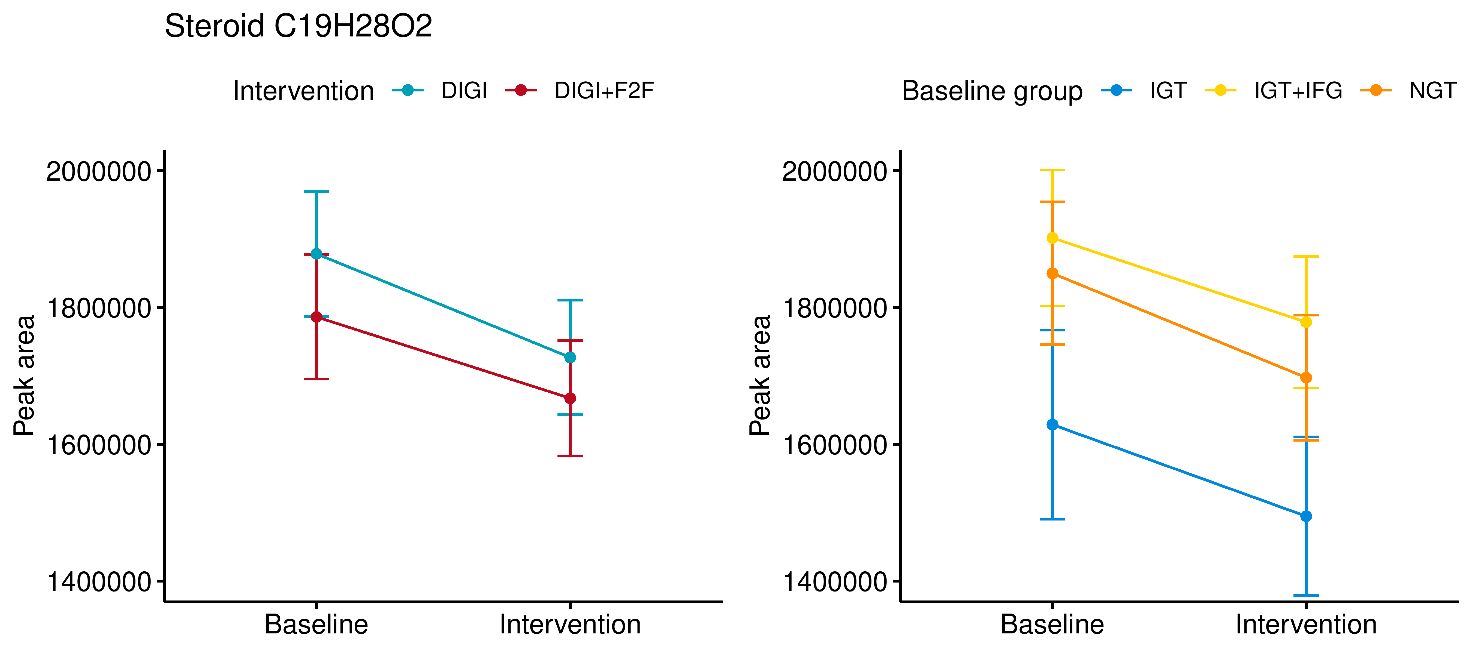

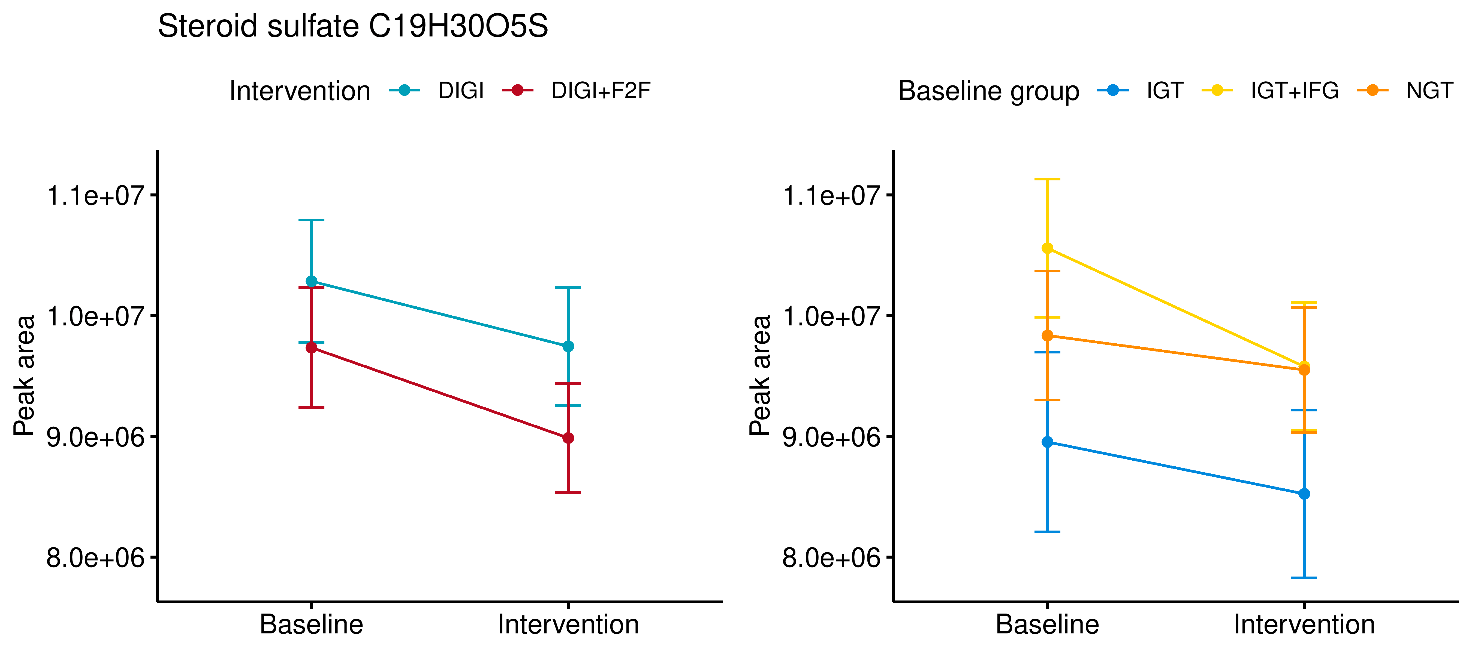

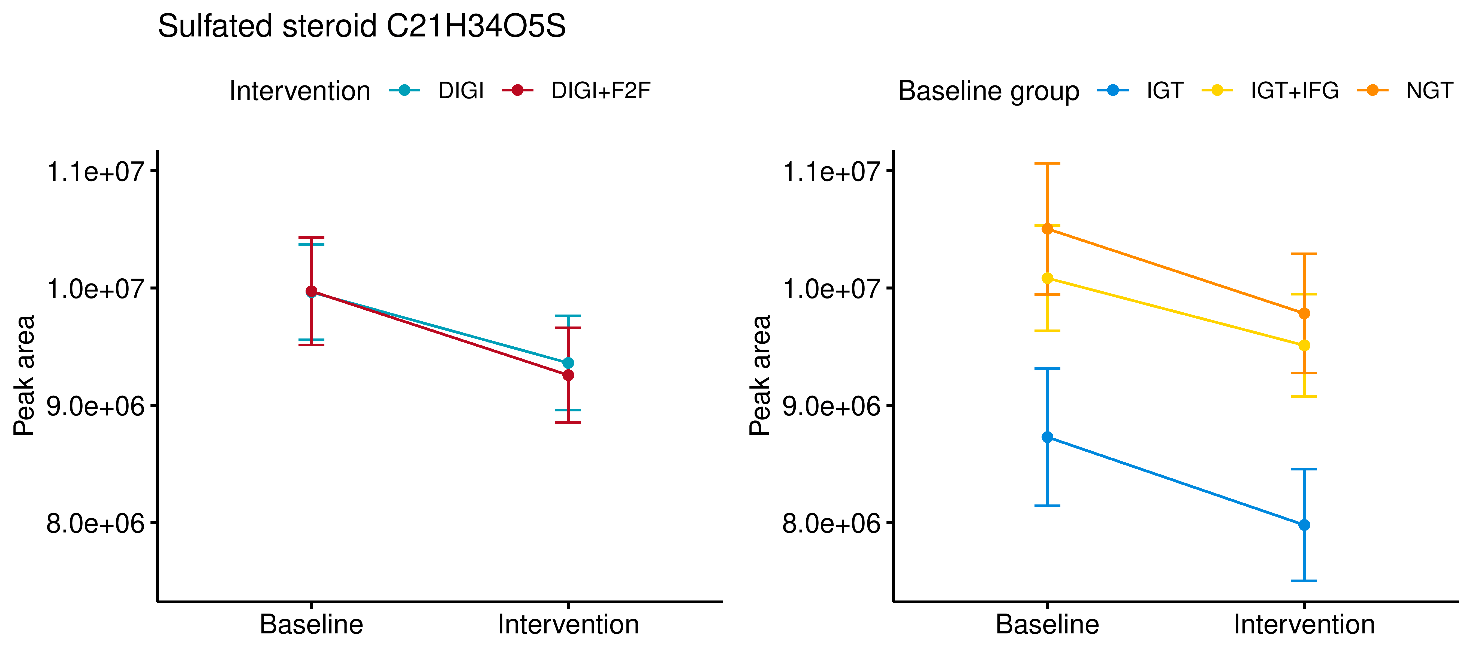

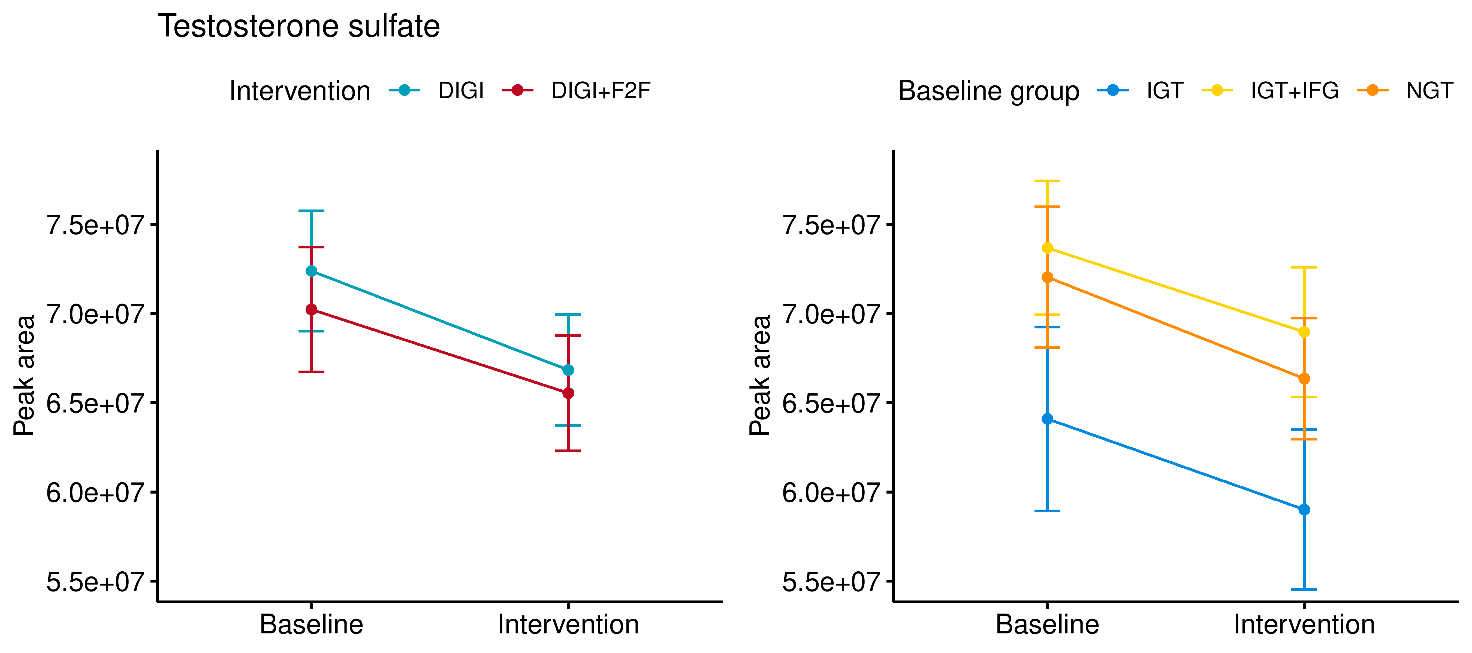

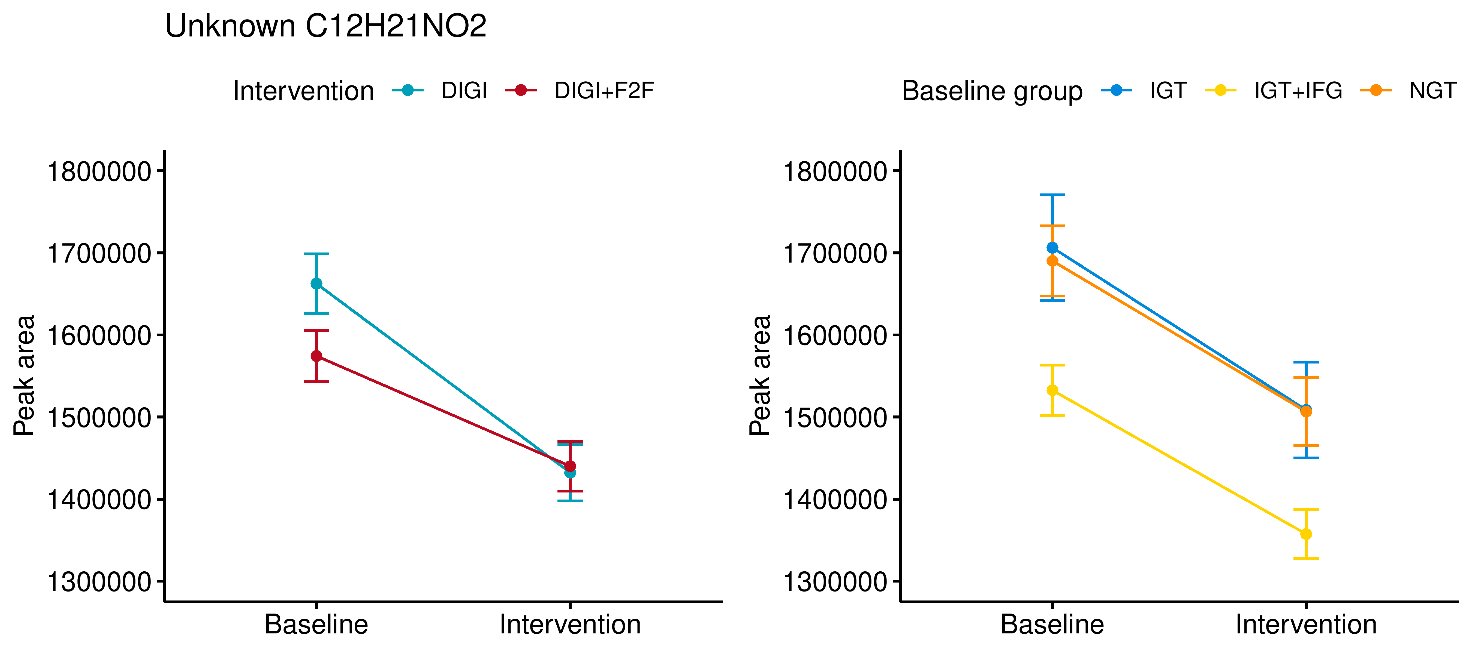

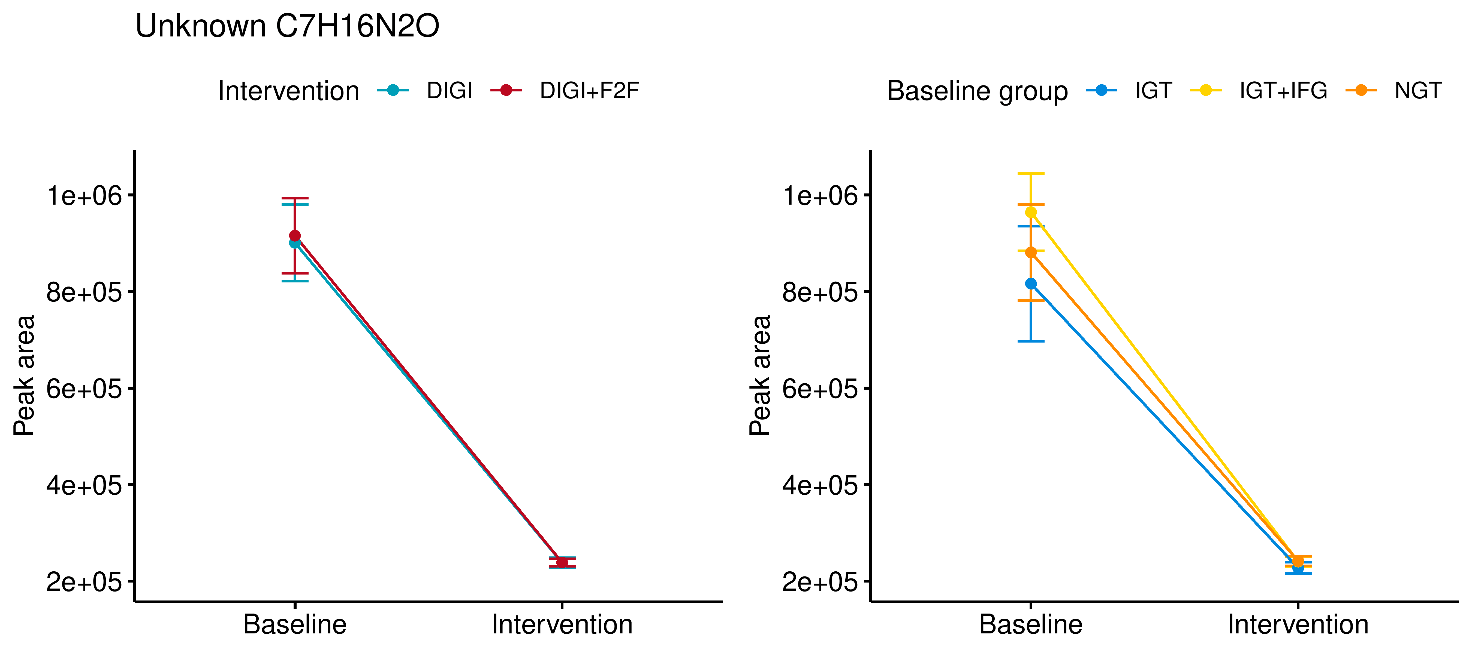

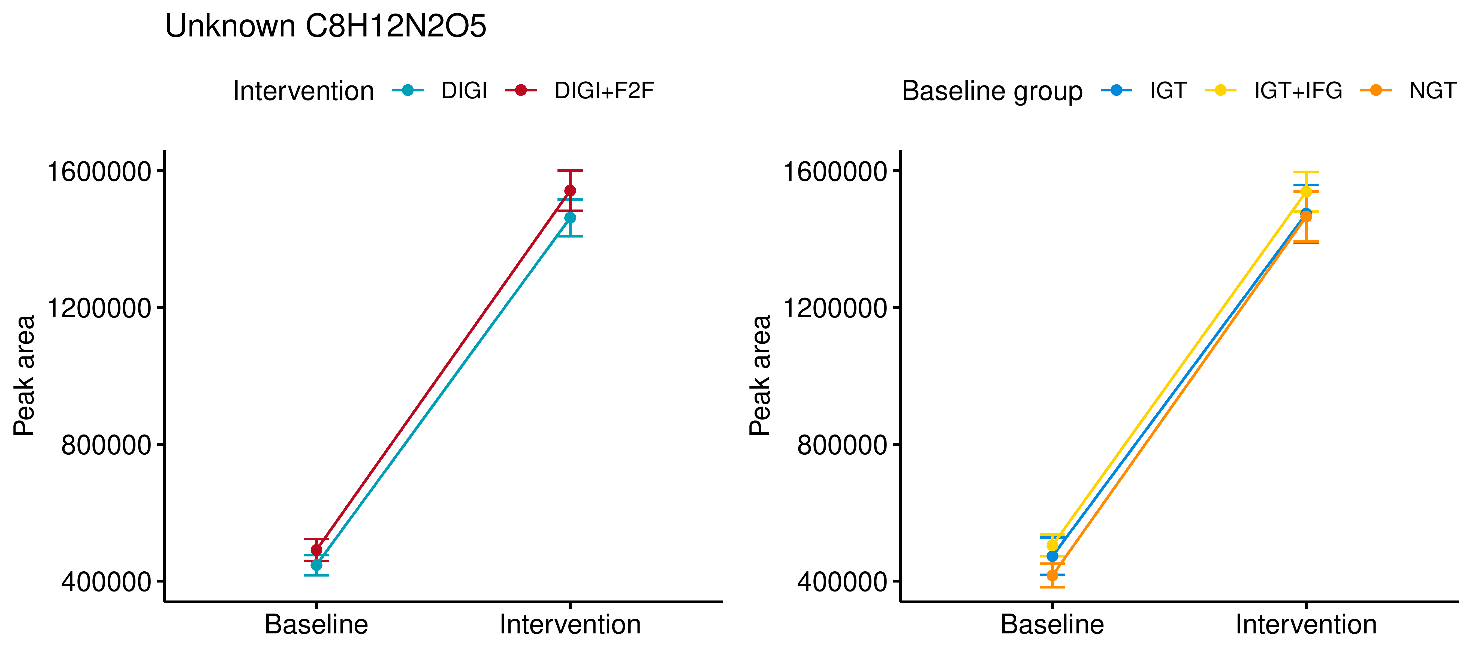

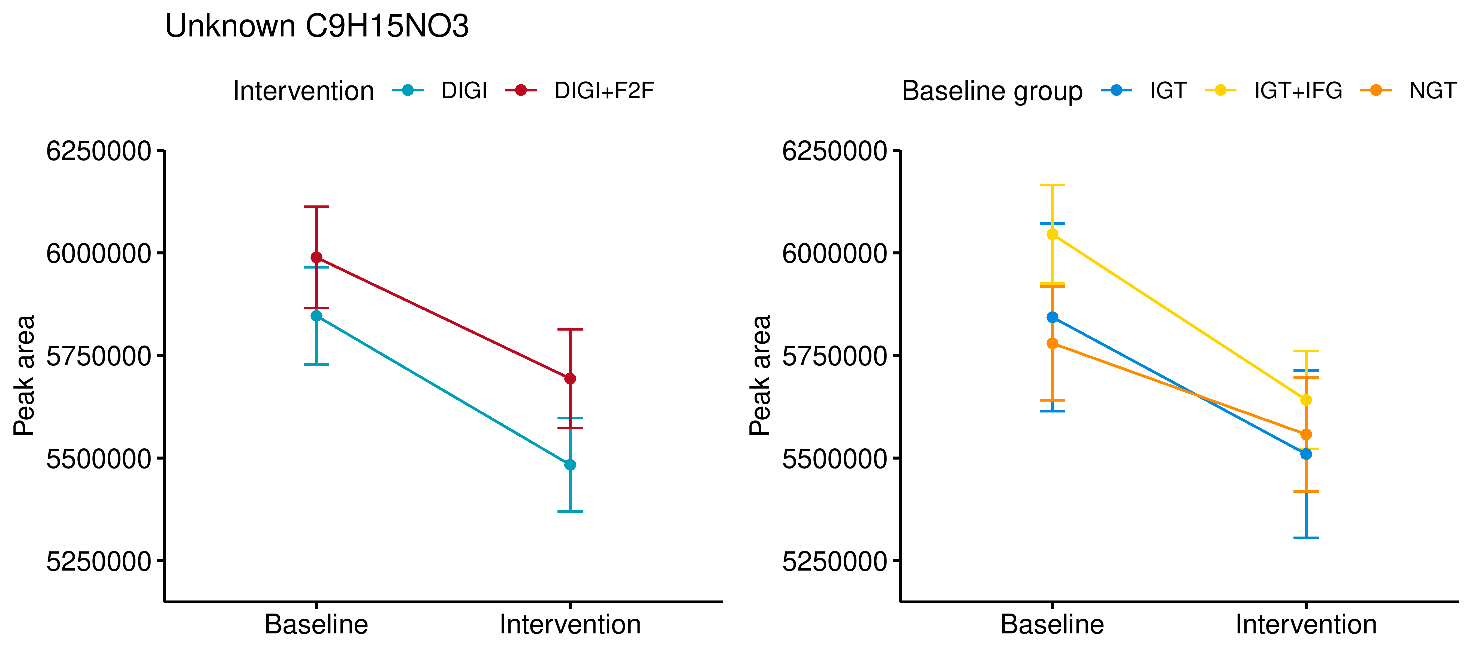

Supplement: Supplementary file 1 — Supplementary Material 1 [file 41598_2025_25749_MOESM1_ESM.docx]
